# Preferential nitrogen retention characterizes current eutrophication in United States lakes

**DOI:** 10.1038/s41467-026-75318-9

**Published:** 2026-07-09

**Authors:** Ran Zhou, Josep Peñuelas, Jordi Sardans, Yindong Tong, Jinlong Zhang, John A. Dearing, Meng Liu, Zisong Chen, Xinyi Wu, Shu Tao, Xilong Wang

**Affiliations:** 1 College of Urban and Environmental Sciences, Peking University, Beijing, China; 2 CSIC, Global Ecology Unit CREAF-CSIC-UAB, Bellaterra, Spain; 3 School of Environmental Science and Engineering, Tianjin University, Tianjin, China; 4 Palaeoecological Laboratory, Geography and Environment, University of Southampton, Southampton, UK

**Keywords:** Freshwater ecology, Biogeochemistry, Geochemistry

## Abstract

The relative retention of nitrogen (N) and phosphorus (P) in lakes and their roles in nutrient cycling remain debated. Although the United States has implemented long-term P control measures in lakes, inexplicable eutrophication exacerbation still reoccurred over the past two decades. Here we geographically track and analyze the nutrient retention changes of 121 lakes in the USA during 2012-2022 using a combination of machine learning and Bayesian hierarchical methods. We find that the preferential retention of P in 70.3% lakes in 2012 shifted to that of N in 85.1% lakes in 2017, and 92.6% lakes in 2022. Four key lake indicators exhibit flickering-like dynamics, which may help identify the nutrient metabolic disorder-like state in the aquatic ecosystem. Overall, our results show that eutrophication in U.S. lakes during 2012-2022 is advancing on the basis of preferential and net nutrient retention shift from P to N, flickering-like dynamics, and associated occurrence of the nutrient metabolic disorder-like state that are all predominantly driven by internal cycling instead of external loading. These patterns are consistent with the possibility of a regime shift rather than simple eutrophication recurrence, and provide insights relevant to lake management and water quality improvement strategies.

## Abstract (web summary)

Lake eutrophication has long been attributed to phosphorus. Tracking 121 U.S. lakes, the authors show a shift to nitrogen retention associated with internal cycling, with fluctuating nutrient dynamics suggesting a potential regime shift.

## Introduction

Eutrophication has been accepted as one of the most pressing environmental problems at global scale^1^. Phosphorus (P) has been widely viewed as a nutrient that constrains biotic production of freshwater^2^. A previous study indicates that almost 90% of global lakes show preferential retention of P^3^. This further fuels the long-standing debate for prioritization of P pollution mitigation^4^ and weakens the argument for N reduction^5–7^. Over the past seven decades, human activities have drastically altered the N/P stoichiometric ratio in water, and added more N than P to the biosphere^8^. This drives the establishment of P limitation in lakes since the mid-20th century and decreases the attention to the importance of N control^9^. However, there remain two primary controversies in the academic community.

The first core controversy centers on this question: in the context of mitigating eutrophication, which plays a more critical role—N or P? Generally, compared to N, P is more significant in terms of the contribution of internal cycling to nutrient dynamics^3^. Evidence of in-lake preferential uptake of P has also prompted efforts to reduce its inputs in the hope that eutrophication can be controlled^10^. Yet, there have been some new discoveries in nutrient dynamics with the new crisis of eutrophication in the past decade. It is widely accepted that the imbalanced nutrient load reductions lead to marked increases in TN/TP stoichiometric ratios and more significant P limitation in lakes^11,12^. However, several studies in the last decade have shown that the nutrient-limited state of phytoplankton may revert from P to N limitation^13,14^, with improvement of industrial TN removal efficiency, increase of atmospheric P deposition, and slight reduction of N deposition^15^. Meanwhile, wastewater treatment plants generally retain approximately 60% of N and 80% of P^16^, and together with a series of P control measures applied between the 1970s and 1990s, did achieve some eutrophication alleviation in some countries in the Northern Hemisphere^10^, but lake eutrophication in these countries staged a comeback again in the last 20 years (defined as a new wave of eutrophication since the 21st century^17^. This phenomenon is especially significant in countries such as the USA, where P control has become the dominant view; lake eutrophication continued to exacerbate^18,19^. Specifically, the USEPA reports show that the percentage of lakes in the USA with good chlorophyll-a condition (Chla <7 µg·L^−1^ roughly) decreased from 46% in 2012 to 34% in 2017, and further to 31% in 2022^20^. No consensus on the underlying causes responsible for the new wave of eutrophication has been reached in the 21st century^21,22^. These findings challenge the current predominant strategy of solely controlling P.

**Fig. 1:**
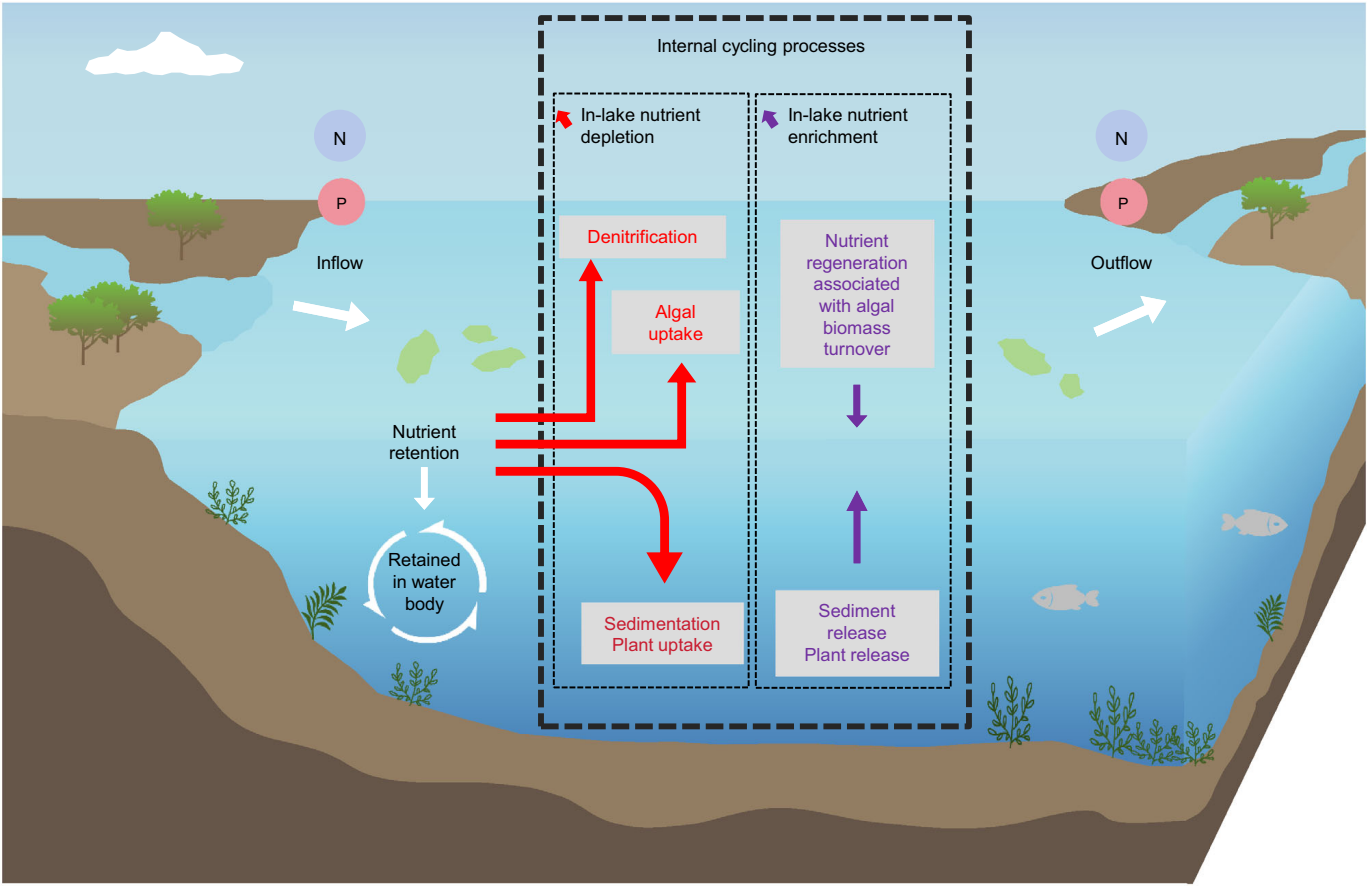
A diagram showing the nutrient (N and P) cycling processes and nutrient retention. The internal nutrient cycling processes include two processes: (1) in-lake nutrient enrichment, which mainly refers to input of nutrients from sediment release and nutrient regeneration associated with algal biomass turnover, and (2) in-lake nutrient depletion, which means removal of a part of existing nutrient and that retained from inflows by denitrification, sedimentation, and algal uptake. Nutrient retention includes nutrients retained in the water body and those removed through in-lake depletion. Net nutrient retention is derived from nutrient retention by excluding the contribution of in-lake nutrient enrichment.

Another core controversy in research on eutrophication mechanisms revolves around this question: between the internal nutrient cycling and external input loading, which plays a more significant role in driving the eutrophication process? For a long time, reducing internal loading is a secondary consideration due to complexity of the internal nutrient cycling^17^. However, recent studies show that internal cycling contributes more than external loading to lake N and P changes^23,24^. Additionally, whether the nutrient in lakes is predominantly limited by N or P depends on the in-lake nutrient dynamics^24,25^. Thus, understanding of internal nutrient cycling that includes in-lake nutrient enrichment and depletion^3,26^ and nutrient retention which includes natural internal retention within—and natural losses from—lakes^27^ is also considered essential to eutrophication mitigation (Fig. 1).

The lakes in the USA have been polluted and since many have been remediated, they are at the forefront of the development history of lake pollution remediation in the world. Given that prioritization of P mitigation has been recognized by policy makers, managers have to rely on traditional means such as reducing excess external nutrient loading, sediment dredging, aeration of the hypolimnetic water, and in situ chemical inactivation to control P^28–30^ based on the cognition of the old paradigm of eutrophication. Notably, the old paradigm is conventionally derived from statistical analysis but lacks continuous monitoring of the same lakes^31,32^, which is problematic or even misleading and subsequently may lead to formulation of non-tailored and ineffectively targeted restoration strategies. The problem is that the statistical analysis methodologies typically involve the selection of distinct sample groups during diverse sampling years^33^. However, such an old paradigm has ingrained in researchers a rigid perception regarding P control in U.S. lakes^34^. Definitely, a more preferable yet currently scarce approach is to conduct one-to-one tracking of the nutrient status of lakes on a large scale, since the geophysical, nutrient, and landscape indicators among different lakes exhibit substantial and pronounced variations^35^. Here we systematically screen 121 lakes in the USA and compile a panel dataset named United States Lakes (USL) dataset, and analyze their nutrient dynamics across three sampling years (2012, 2017, and 2022). We employ a cohort-style tracking study aiming to address the following knowledge gaps. 1) To what extent has preferential retention of P shifted to N over the past decade, thereby influencing the effectiveness of P control strategies? 2) Given that the external loading that may influence eutrophication of these lakes did not change significantly over the past decade^20^, have substantial changes in net N and P retention occurred? If so, has the net nutrient retention change in these lakes been dominated by external loading or internal cycling? and 3) Has a new wave of eutrophication occurred in the lakes, and are they undergoing a regime shift, rather than just an ordinary recurrence of eutrophication?

To systematically address these knowledge gaps based upon the above mentioned datasets, we employ a progressive approach by combining machine learning and Bayesian hierarchical method to track changes in nutrient retention in 121 lakes from 2012 to 2022. This framework leverages the high predictive accuracy of machine learning for estimating net anthropogenic nutrient inputs (*f*_NANI_ and *f*_NAPI_)^36,37^, and the strong ability of Bayesian hierarchical models to handle uncertainties and internal nutrient cycling processes^38,39^. The integration of machine learning with dynamic nutrient-driven models based on Bayesian hierarchical method represents a methodological innovation that effectively combines data-driven pattern recognition with process-based biogeochemical modeling, enhancing the accuracy of simulating nutrient dynamics across spatiotemporal scales.

## Results and discussion

### Shift in lake preferential and net nutrient retention

**Fig. 2:**
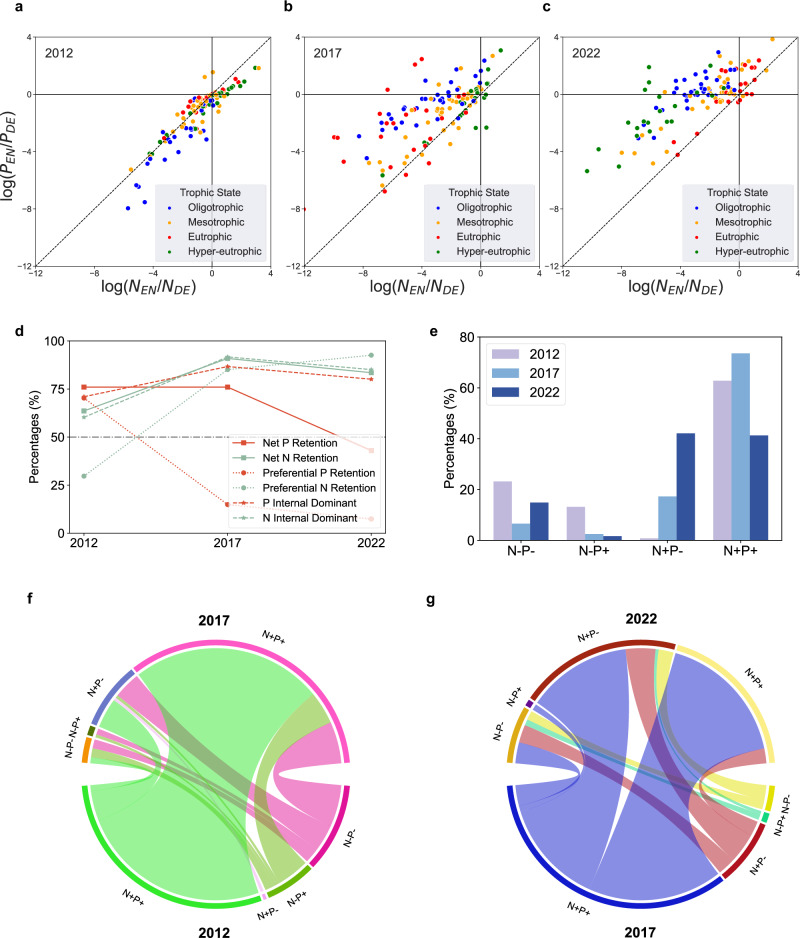
Pattern of nutrient retention. **a** Significant dominance of preferential P retention in 2012 (two-tailed t(120) = −4.85, *p* < 0.001, Cohen’s d = −0.44, 95% CI [−0.57, −0.24]); and **b** significant dominance of preferential N retention in 2017 (two-tailed t(120) = 10.81, *p* < 0.001, Cohen’s d = 0.98, 95% CI [0.57, 0.83]); and **c** significant dominance of preferential N retention in 2022 (two-tailed t(120) = 17.77, *p* < 0.001, Cohen’s d = 1.62, 95% CI [0.76, 0.95]). *P*_EN_ and *N*_EN_ mean in-lake enrichment of P and N, respectively. *P*_DE_ and *N*_DE_ mean in-lake depletion of P and N, respectively. Lakes retain nutrients through in-lake depletion processes; while in-lake enrichment supplies nutrients in excess of those depleted, lakes can no longer be considered net nutrient sinks. Accordingly, log(*N*_EN_/*N*_DE_) < 0 and log(*N*_EN_/*N*_DE_) ≥ 0 are defined as positive and negative net retention of N, respectively, and the same criteria are applicable for P^3^. Furthermore, preferential N retention is defined^3^ as *P*_EN_/*P*_DE_ > *N*_EN_/*N*_DE_, while preferential P retention is defined as *P*_EN_/*P*_DE_ < *N*_EN_/*N*_DE_. Lakes located above (below) the black dashed y = x line show a pattern of preferential N (P) retention. **d** Changes in the proportion of different types of lakes from 2012 to 2022. **e** Percentages of four types of lakes in 2012 and 2017. N+ means positive net N retention while N- means negative net N retention. P+ means positive net P retention while P- means negative net P retention. **f** Conversion of four types of net nutrient retention from 2012 to 2017; and **g** from 2017 to 2022. Source data are provided as a Source Data file.

The nutrient retention pattern in 121 lakes that were screened for tracking in the USA was verified using their datasets (see Methods, USL data). On the basis of the concept of nutrient retention, Wu et al.^3^ further defined the preferential and net nutrient retention in lakes (Fig. 2). Based on the in-lake enrichment (*P*_EN_ for P or *N*_EN_ for N) and depletion (*P*_DE_ for P or *N*_DE_ for N) of each lake output by the model (Supplementary Note 1 & Supplementary Figs. S1–S7), we analyzed the preferential retention and net retention of each lake. Among the studied lakes, most exhibited preferential P retention in 2012 (70.3% below the dashed line; Fig. 2a), consistent with previous findings^3^. From 2012 to 2022, this pattern progressively shifted toward preferential N retention. The proportion of lakes exhibiting preferential N retention increased to 85.1% in 2017 and further to 92.6% in 2022 (above the dashed line; Fig. 2b, 2c), accompanied by a sharp decline in preferential P retention from 14.9% in 2017 to 7.4% in 2022 (below the dashed line; Fig. 2b, 2c). Collectively, these results reveal a major shift in preferential nutrient retention across U.S. lakes between 2012 and 2022. This transition toward preferential N retention, largely unanticipated under historically P-dominated conditions, suggests that lakes experiencing ongoing eutrophication can undergo a substantive reorientation in nutrient retention, shifting from a long-standing preference for P toward increasing N retention.

Significant changes have also occurred in the net retention of both nutrients defined by the equations of *N*_EN_/*N*_DE_ < 1 for N or *P*_EN_/*P*_DE_ < 1 for P. From 2012 to 2017, the lake number with net P retention remained relatively stable at 76.0%, while that of lakes with net N retention surged from 63.6% to 90.9% (Fig. 2d). This trend is consistent with previous studies^3,40^ and suggests the transition in the preferential nutrient retention from P to N in lakes was accompanied by a unilateral increase in the trend of net N retention. However, from 2017 to 2022, the number of lakes with net P retention sharply declined to 43.0%, while that of lakes with net retention of N only slightly decreased to 83.5% (Fig. 2d). Further analysis shows that the performance of lakes in each ecological region in the preferential and net retention of N and P are consistent with the overall pattern (Supplementary Note 2 & Supplementary Figs. S8–S10).

Long-term studies on the relationship between preferential and net retention of N and P are divergent. For instance, it is reported that enrichment of P during cyanobacterial blooms can fuel nitrification activities in sediment which in turn may lead to N deficiency^41^. However, Finlay et al.^42^ found that steep declines of P availability and productivity made N concentrations sharply rise. By tracking the nutrient dynamics of 121 lakes in the USA, we find that accompanying the shift in dominance of the preferential nutrient retention from P to N, the number of the N + P- group lakes significantly increased while that of the N-P+ group lakes significantly decreased (Fig. 2e–g). This pattern is consistent with the inhibitory effect of preferential N retention on net P retention. To further test this interpretation, we conducted a causal inference analysis using a two-way fixed-effects Logit model, which provides additional support for a negative effect of preferential N retention on net P retention (Supplementary Note 3 & Supplementary Table S1).

**Fig. 3:**
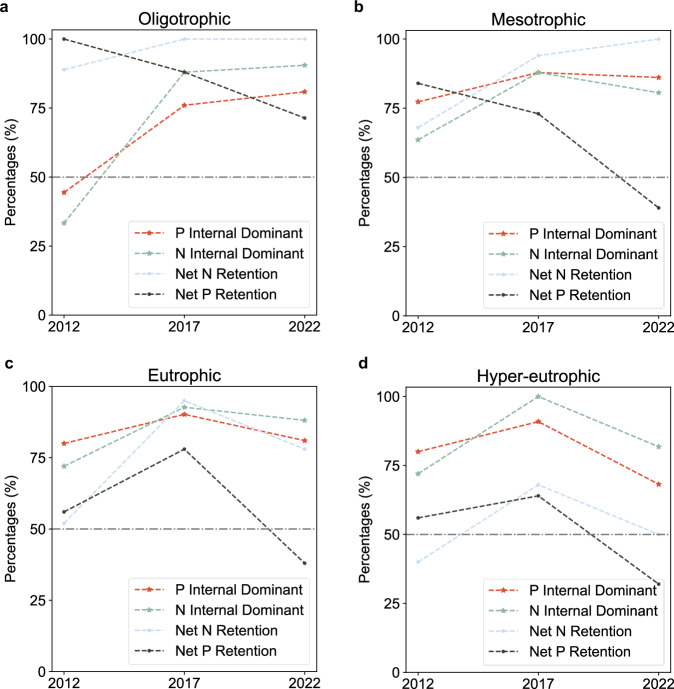
The percentage changes of the lakes that exhibited net N (P) retention and internal dominant model. **a** Oligotrophic group lakes; **b** Mesotrophic group lakes; **c** Eutrophic group lakes; **d** Hyper-eutrophic group lakes. INCI is calculated as follow, see Methods for details of parameters. Here, ${N}_{{{{\rm{INCI}}}}}=(|{N}_{{{{\rm{EN}}}}}|+|{N}_{{{{\rm{DE}}}}}|)/({L}_{{{{\rm{N}}}}}/{{{\rm{V}}}})$; ${P}_{{{{\rm{INCI}}}}}=(|{P}_{{{{\rm{EN}}}}}|+|{P}_{{{{\rm{DE}}}}}|)/({L}_{{{{\rm{P}}}}}/{{{\rm{V}}}})$. Source data are provided as a Source Data file.

We find that the lakes as nutrient sinks are experiencing a weakening tendency to net retention of nutrients (Fig. 3). The proportion of lakes with positive net P and N retention generally rapidly decreases as trophic levels increase (Fig. 3), indicating a negative feedback of these two nutrients and the lakes with more severe pollution are more unfavorable to their net retention. The logistic regression analysis we performed as a supplementary analysis also consistently showed the same tendency (Supplementary Note 4 & Supplementary Fig. S11). This also reflects that the retention capacity of lakes is by no means limitless. When eutrophication of lakes exacerbates to a certain threshold, they have contained a significant amount of nutrients, which may strongly constrain their ability to retain nutrients.

### The contribution of internal nutrient cycling to nutrient retention

Nutrient retention could either be dominated by external loading or internal nutrient cycling. Due to the complexity of internal cycling processes^3,24^, it is often challenging to take effective measures before clarifying the roles of the internal cycling in nutrient retention and associated eutrophication. Over the past two decades, an increasing body of evidence has suggested that internal cycling of N and P in shallow lakes may be one of the main driving forces responsible for eutrophication^43,44^. To find out whether nutrient retention is predominantly driven by internal cycling or external loading, an index of Internal Nutrient Cycling Intensity (INCI) proposed by Wu. et al.^3^ was used. INCI is calculated based on model-derived estimates of in-lake nutrient enrichment, depletion, and external nutrient loading (Fig. 3 caption, Supplementary Notes 1, 4 & 5). We find that, in the three surveyed years (2012, 2017, and 2022), the net retention of both N and P in the lakes in the USA was primarily driven by internal cycling rather than external loading (Fig. 3 red dashed line and green dashed line, & Supplementary Figs. S12–S15), aligning with the USEPA report showing no significant impact of anthropogenic factors on lake nutrient condition during 2012-2022^20^. The number of lakes at a given trophic level in which their net N and P retention was predominantly regulated by internal cycling generally showed an initial increase followed by a decrease from 2012 to 2022 (Fig. 3). Furthermore, the proportion of lakes where net N and P retention was predominantly regulated by internal cycling increased with trophic level in oligotrophic systems, and this tendency became non-monotonic in mesotrophic and more eutrophic lakes, first increasing and then declining at higher trophic levels (Fig. 3). This non-monotonic response may suggest a weakening of internal cycling dominance at advanced stages of eutrophication.

### Flickering-like dynamics and nutrient metabolic disorder like state

**Fig. 4:**
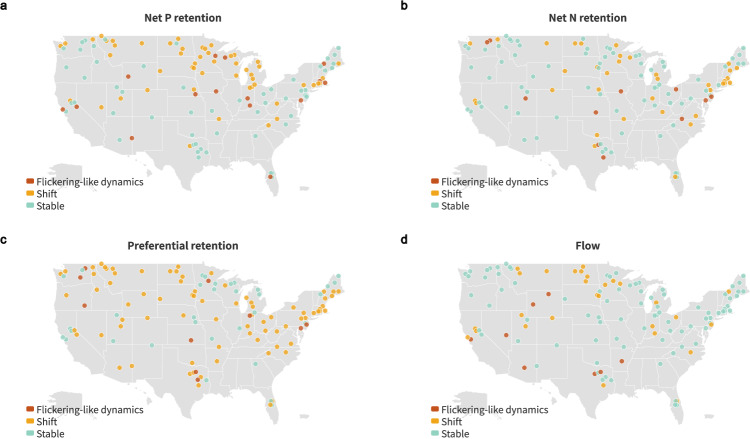
Spatial distribution of the flickering-like dynamics of key metrics. The types can be categorized based on the number of shifts as: 2 shifts (flickering-like dynamics), 1 shift (shift), and no shift (stable). Four graphs show flickering-like pattern of **a** net P retention switching between positive and negative, **b** net N retention switching between positive and negative, **c** preferential nutrient retention switching between N and P, **d** flow N/P switching between inflow N/P over outflow N/P and outflow N/P over inflow N/P. Source data are provided as a Source Data file.

By tracking the nutrient dynamics changes of 121 lakes, shifts and fluctuations in four key indicators (i.e., net N retention, net P retention, preferential nutrient retention and flow N/P) for many lakes are observed (Fig. 4). Such a phenomenon could be flickering, since it has been proposed to describe the phenomenon that causes the system to switch back and forth between alternative states under relatively great influence^45^. This phenomenon has been shown in models of trophic cascades and eutrophication. These shifts align with the suite of characteristics of flickering, rather than with interannual variability, diffusion of nutrients, and data distortion (Supplementary Notes 6 & 7). The occurrence of flickering-like dynamics suggests patterns consistent with approaching a critical point in nutrient cycling^22,45^, at which qualitative changes in nutrient processing are theoretically expected.

**Fig. 5:**
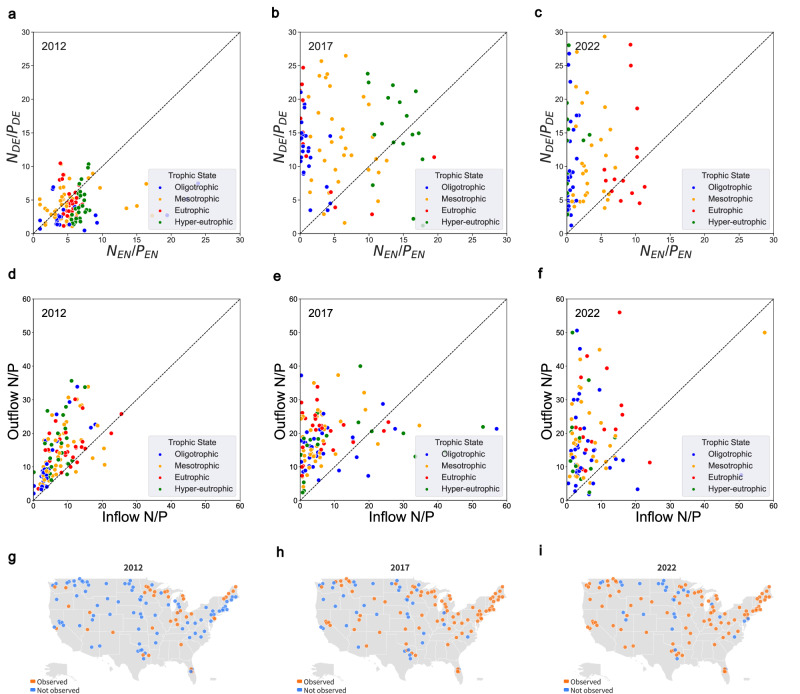
Stoichiometry of nutrient cycling in 2012 and 2017. The N/P ratios of in-lake enrichment and depletion **a** in 2012, **b** 2017, and **c** 2022. These panels replicate the preferential retention pattern shown in Fig. 2a-c, and the corresponding statistical tests yielded identical results. The outflow N/P ratios exceeded inflow ratios **d** in 2012 (t(120) = 17.77, *p* < 0.001, Cohen’s d = 1.62, 95% CI [0.76, 0.95]), **e** 2017 (t(120) = 9.04, *p* < 0.001, Cohen’s d = 0.82, 95% CI [0.50, 0.78]) and **f** 2022 (t(120) = 9.87, *p* < 0.001, Cohen’s d = 0.90, 95% CI [0.54, 0.80]). The Eqs. 11–14 in the Methods section are the details for inflow and outflow derivation. The proportions of lakes in NMD-like paradoxical state **g** count for 38% in 2012, **h** 69% in 2017, and **i** 76% in 2022. Source data are provided as a Source Data file.

Consistent with the theoretical expectation associated with flickering, our results further reveal that between 2012 and 2017 the nutrient dynamics of lakes across the USA underwent a pronounced and system-wide shift, exhibiting anomalous characteristics consistent with a nutrient metabolic disorder-like (NMD-like) pattern (Fig. 5g–i). In 2012, P was more preferentially retained than N (Fig. 5a), and the model-derived outflow N/P ratios exceeded inflow ratios (Fig. 5d), consistent with theoretical expectations and previous studies^3^. However, most lakes exhibit preferential retention of N after 2017 (Fig. 5b, c), while paradoxically maintaining substantially elevated outflow N/P ratios relative to inflow (Fig. 5e, f). The emergence of this counterintuitive and widespread pattern across a majority of lakes suggests a potential transition to a distinct nutrient processing regime. The defining features of this regime conceptually resemble metabolic disorder observed in humans (Supplementary Note 8, Supplementary Table S2). Previous studies have primarily focused on changes in absolute nutrient quantities, which may not fully capture imbalances between N and P. Such imbalances may therefore have been less readily detected. As a result, this peculiar phenomenon remained undetected for a long time. It reflects the contradiction of the mass balance of N and P determined by the internal cycling process occurring in lakes, challenging the status of lakes as nutrient sinks^46^.

### The new wave of eutrophication in U.S. lakes since the 21^st^ century

**Fig. 6:**
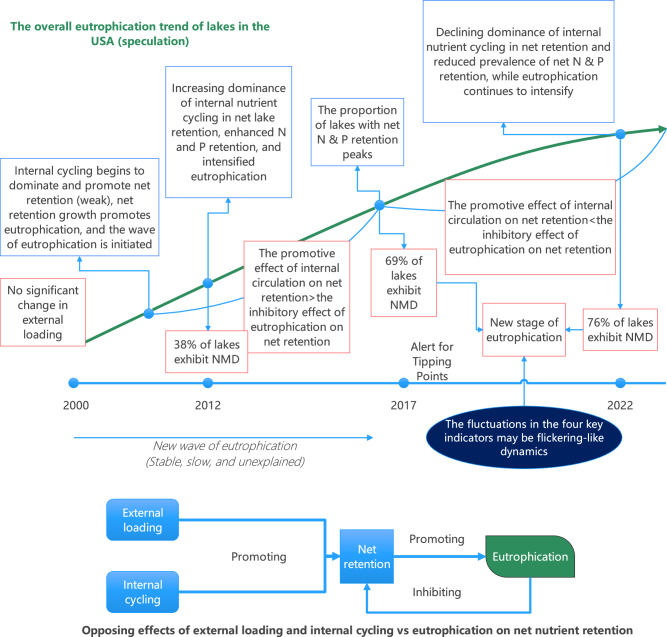
The potential overall eutrophication trajectory of lakes in the USA. This conceptual diagram presents a hypothesized eutrophication trajectory of lakes in the USA based on the findings of this study. The lower section is a schematic representation included to facilitate understanding of the opposing influences of external loading and internal cycling, and eutrophication, on net nutrient retention.

U.S. lakes have been at the forefront of the new wave of eutrophication since the 21st century, demonstrating a slow, yet steady, growth of eutrophication trend during 2012-2022 (Supplementary Note 9 & Supplementary Figs. S16–S18). Yet, there has been no significant change in external loading of U.S. lakes (Supplementary Note 4). Based on the integrated analysis of this study and the existing literature, we propose a conceptual framework to systematically explain the nutrient evolution of U.S. lakes in the new wave of eutrophication since the early 21st century. Over the past two decades, the dominance of internal cycling has led to sustained net retention, reigniting eutrophication, indicated by the fact that the number of lakes in the USA with net retention of N and P continued to increase until 2017, and the net nutrient retention was primarily driven by internal cycling (Fig. 6). After the new wave of eutrophication reaches a certain stage, the inhibitory effect of nutrient on its net retention has exceeded the promotive effect from internal cycling, since the trend of net nutrient retention weakens with the progression of eutrophication. Most lakes have reached their net retention capacity, as evidenced by the net retention of N and P beginning to decline during 2017–2022.

The seemingly slowly-increasing eutrophication trend is consistent with the lakes facing conditions that may precede a regime shift. This interpretation is supported by the repeated switching of four key indicators, indicative of flickering-like dynamics and the emergence of an associated NMD-like state, rather than by a P-control-induced shift in the TN/TP ratio (Supplementary Fig. S19). One should not regard it as an ordinary wave of eutrophication, thus seriously underestimating its potential ecological risks. Analogous to the irreversible tipping points projected to occur once the 1.5 °C global warming threshold is surpassed, the NMD-like state may pose a substantial risk of inflicting severe ecological and socioeconomic consequence if not mitigated in a timely manner.

### Model uncertainty and sensitivity

The uncertainties of the machine learning outputs for *f*_NANI_ and *f*_NAPI_ were quantified using R² (coefficient of determination) and MSE (mean squared error). Based on the data-driven principles, we selected the Random Forest (R² = 0.71) with the smallest prediction error for *f*_NANI_ and the Extra Tree (R² = 0.53) for *f*_NAPI_, respectively (Supplementary Fig. S20 & Supplementary Tables S3 & S4). We conducted additional uncertainty and sensitivity analyses to assess the influence of *f*_NAPI_ uncertainty on the main conclusions (Supplementary Note 10, Supplementary Figs. S21 & S22).

The uncertainties of the sub-parameter estimates and model-derived results from the in-lake nutrient budget model were incorporated into the analysis. We quantified uncertainties of the output sub-parameter estimates using MCSE (Monte Carlo standard error), STD (standard deviation), 5%-95% confidence intervals, and ESS (effective sample size) (Supplementary Note 10, Supplementary Tables S5 & S6). Comprehensive evaluation results indicate that the uncertainties of all output sub-parameter estimates remain at a low level. The uncertainty of the model-derived results can be quantified using misclassification percentage error (Supplementary Note 10, Supplementary Tables S7 & S8). The error margins for all results do not exceed: net N retention ( ± 0.80 pp, percentage point), net P retention ( ± 0.40 pp), preferential retention ( ± 0.53 pp), N internal dominant model ( ± 6.4×10^−5^ pp), P internal dominant model ( ± 0.014 pp). The uncertainty associated with the model-derived results is tightly constrained, supporting the robustness of using percentage calculations of categorical variables over calculations of traditional quantitative variables.

Moreover, due to advantages of the Bayesian hierarchical method in handling implicit assumptions, we even obtained full posterior distributions for sub-parameters in each group to characterize the sensitivity of output sub-parameter estimates to 1000 sets of input parameters through Hamiltonian Monte Carlo (Supplementary Figs. S23 & S24). Based on the posterior distributions, the sensitivity of model-derived results to input parameter variability was further evaluated (Supplementary Table S9). The results indicate that both model-derived results and sub-parameter estimates displayed low sensitivity to variations of the input parameters.

### Lake management policies and global implications regarding eutrophication

In summary, our findings indicate that the current eutrophication in U.S. lakes can no longer be solved solely by loading reduction strategies. We strongly recommend implementing emergency nutrient removal projects that employ direct physical or chemical methods to segregate nutrients. We believe that such efforts should currently prioritize N removal. Although this could face high costs for lake ecosystem restoration, it is imperative to prevent lakes from transitioning into an irreversible state after undergoing flickering-like dynamics, thereby potentially yielding superior long-term cost-effectiveness in terms of remediation.

In addition, this study on U.S. lake eutrophication provides important insights for addressing global waterbody nutrient dynamics and eutrophication-related water quality exacerbation. First, the USA is among the first countries to experience water eutrophication (in the 1960s) and to implement systematic eutrophication management frameworks. Second, the USA has established globally recognized lake ecological monitoring frameworks, including the Long-Term Ecological Research Network (LTER) and the National Lakes Assessment (NLA) which has been conducted every five years since 2007, providing significant support and scientific basis for large-scale studies on patterns of lake nutrient dynamics. Third, the diverse lake ecosystems across the contiguous United States span eight climate zones and are delineated into Level IV Ecoregions, which offer a natural laboratory for investigating eutrophication mechanisms across climatic, geographical, and anthropogenic gradients. Such diversity enhances the relevance of U.S.-based findings to broader global contexts.

Based upon the above, a deeper exploration of new mechanisms of N and P cycling dynamics in U.S. lakes and their relationship with eutrophication trends not only advances beyond traditional management strategies, but also provides critical insights for addressing global climate change challenges and developing scientifically effective management policies. Therefore, this study provides a theoretical support for developing comprehensive and effective global strategies for protecting and managing water quality and combating eutrophication in lakes and associated aquatic systems.

Employing a progressive framework, this study systematically addresses the following scientific voids by integrating a fully-developed nutrient budget model based on Bayesian hierarchical method and machine learning, and applying it to a multi-party measured data-based panel dataset. During 2012–2022, the preferential nutrient retention in lakes in the USA generally shifted from P to N, and the nutrient with a stronger tendency of preferential retention may enhance its own net retention while inhibit the other. The number of lakes with net N retention has increased by more than 30%, while that with net P retention sharply decreased by 40%. The shift in net and preferential retention indicates that the U.S. lake ecosystems by no means adhere to an unchanging paradigm. Instead, they are fully capable of spontaneously adjusting their nutrient preferences. In scenarios where P is regulated, these ecosystems will automatically transit to prioritize the retention of N. Above 70% of lakes with both net N and P retention in the USA under study are dominantly contributed by internal cycling, highlighting its significance in controlling nutrient condition. Nutrients exert a negative feedback effect on net retention, contrary to the promoting effect of internal cycling on net retention. A flickering-like phenomenon indicated by switching back and forth between two states of four key indicators was observed in many lakes in the USA. As a critical point predicted by flickering-like dynamics, a new NMD-like state in lakes was identified on a large scale, showing that the lakes exhibit both preferential retention and preferential release of the same nutrient. This hitherto undetected phenomenon is consistent with the possibility that the new eutrophication wave of lakes is undergoing a regime shift. It could be that an increase in net retention of N and P in lakes has initiated the new wave of eutrophication. After 2017, the retention capacity of lake ecosystems reached their limit, leading to the widespread emergence of the NMD-like state. The NMD-like state may be one of the concomitant phenomena of the regime shift occurring in lake eutrophication, warning that eutrophication will reach an irreversible catastrophic state. Overall, this work shatters the long-established paradigm of prioritizing P control, offering an innovative perspective on lake governance. To prevent eutrophication of lakes in the USA from reaching an irreversible state, it is imperative to take immediate and effective measures to control the sources of internal N, even if the cost is high.

## Methods

### USL data

United States Lakes (USL) datasets were compiled based on the National Lakes Assessment (NLA) datasets and the datasets of Wu et al.^3^. The lake samples that we investigate are primarily sourced from the NLA datasets. Here, one challenge is how to achieve a one-to-one correspondence between lakes, because the USEPA does not assign a same ID to a specific lake in different years. To address this challenge, we labeled HUC8 (eight-digit Hydrologic Unit Codes) for each lake from NLA site datasets. HUC8 is a hierarchical, numeric code that uniquely identifies hydrologic units used by USEPA. Three datasets of 121 lakes were screened out on the basis of the intersection of the NLA datasets of 2012, 2017 and 2022 according to HUC8.

The following parameters were employed to construct three datasets for 121 lakes including: latitude and longitude from NLA site datasets, total nitrogen content (TN [mg L^−1^]), total phosphorus content (TP [mg L^−1^]) and chlorophyll content (Chla [µg L^−1^]) from NLA chemistry datasets, percentage of the basin area classified as forest (*LU*_forest_ [%]) and wetland (*LU*_wetland_ [%]) from NLA landscape datasets, as well as discharge rate [m^3^ day^−1^], lake volume [m^3^], water residence time (WRT [day]), watershed area [ha] from the datasets of Wu et al^3^. All these data are measured. The details for field sampling, including method, sampling time, and descriptions of parameters are described in Supplementary Note 11.

### ***f***_**NANI**_**&*f***_**NAPI**_**data**

Net anthropogenic nitrogen input (NANI [kg N km^−2^ yr^−1^]) and phosphorus input (NAPI [kg P km^−2^ yr^−1^]) were used in in-lake nutrient budget model. Generally, NANI is defined as the sum of five components: atmospheric deposition, fertilizer nitrogen input, agricultural nitrogen fixation, net food and feed import, and non-food crop export. NAPI is defined as the sum of three components: fertilizer phosphorus input, net food and feed import, and non-food crop export. We performed a transformation on NANI and NAPI based on Eqs. (1) and (2). Both *f*_NANI_ and *f*_NAPI_ were constructed to replace NANI and NAPI mainly for three reasons: The function asinh (inverse hyperbolic sine function), as a monotonic function, is only intended for easier application in the model and does not change the essence of the parameters NANI and NAPI. Both *f*_NANI_ and *f*_NAPI_ are the required terms in the nutrient budget model, and this simplification has already been used in previous studies^47,48^. Both perform well on the USL2012 data with small losses. The *f*_NANI_ and *f*_NAPI_ for 2012 were from the datasets of Wu et al^3^. and the *f*_NANI_ and *f*_NAPI_ for the years 2017 and 2022 were predicted using machine learning.

1 $${f}_{{{{\rm{NANI}}}}}={asinh}(\frac{{{{\rm{NANI}}}}}{2})$$

2 $${f}_{{{{\rm{NAPI}}}}}={asinh}(\frac{{{{\rm{NAPI}}}}}{2})$$

### Machine learning

Modern machine learning demonstrates substantial potential in simulating nonlinear and complex natural systems^49,50^. This study employs some parameters from USL2012 as independent variables while using *f*_NANI_ and *f*_NAPI_ for 2012 as dependent variables in the training set. Through optimization of various parameter combinations and a review of the existing literatures, we identified TN (TP), latitude, longitude and ecoregion as independent variables (Supplementary Fig. S25). To evaluate model performance, we used a range of established machine learning methods on the test set to compare their predictive accuracy. Ultimately, the random forest regression was selected to predict *f*_NANI_ and ExtraTree Regression was utilized to predict *f*_NAPI_.

### In-lake nutrient budget model

In-lake nutrient budget model is a well-known model developed by Carpenter et al.^2,32^. Although the initial model developed in 2003 is relatively simple, it represents the process of eutrophication in lakes and a common mitigation strategy, making it popular and developed by other scientists. The nutrient budget model used in the current study was slightly modified to describe the internal nutrient cycling processes. The input data of the model included TN, TP, Chla, lake area, discharge rates, lake volume, WRT, watershed area, *LU*_forest_, *LU*_wetland_, from USL datasets and *f*_NANI_ and *f*_NAPI_ from *f*_NANI_ & *f*_NAPI_ data. The output data of the model included the posterior distribution of TN (TP), in-lake N (P) enrichment, in-lake N (P) depletion, N (P) loading, N (P) inflow and outflow. Significantly, in-lake N (P) enrichment represents the internal input of nutrients such as sediment release, in-lake N (P) depletion represents the internal removal of nutrients such as sedimentation and denitrifcation^3^. N (P) inflow refers to the nutrients that flow into the lake through various external pathways, and N (P) outflow refers to the nutrients in discharge^3^. It is worth noting that inflow and outflow are generally not obtainable through experiments, primarily because there are numerous external sources of nutrients. Experiments can only sample lake water during specific periods but cannot measure nutrient migration in the atmosphere and groundwater. Virtually all studies depend on model monitoring. Although there is no measured data to validate the output data of the model, the model’s fine simulation of in-lake nutrient dynamics, along with the constraints of Bayesian hierarchical methods on uncertainty, enhanced its reliability^2,38^. Previous studies have shown that Chla can represent the intensity of in-lake enrichment and algal biomass^19^. Given that the relationship between driving factors of eutrophication and biological reactions is usually non-linear, we followed the classic work to describe in-lake nutrient enrichment as a modified Michaelis–Menten function of Chla^2^. The in-lake N enrichment (*N*_*E*N_) and in-lake P enrichment (*P*_EN_) of a lake were calculated using Eq. (3) and Eq. (4); in-lake N depletion (*N*_DE_) and in-lake P depletion (*P*_DE_) of a lake were calculated using Eq. (5) and Eq. (6). *A*_N_ and *A*_P_ are the coefficient of in-lake N and P enrichment (g·day^–1^ N; g·day^–1^ P), respectively. *M*_N_ and *M*_P_ are half saturation constant of Chla for in-lake N and P enrichment (mg·m^–3^ Chla), respectively. *B*_N_ and *B*_P_ are shape parameter of Chla for in-lake N and P enrichment, respectively. *S*_N_ and *S*_P_ are rate of in-lake N and P depletion (day^–1^), respectively.

3 $${N}_{{{{\rm{E}}}}{{{\rm{N}}}}}=\frac{{A}_{{{{\rm{N}}}}}}{V}\times \frac{{{Chla}}^{{B}_{{{{\rm{N}}}}}}}{{{M}_{{{{\rm{N}}}}}}^{{B}_{{{{\rm{N}}}}}}+{{Chla}}^{{B}_{{{{\rm{N}}}}}}}$$

4 $${P}_{{{{\rm{EN}}}}}=\frac{{A}_{{{{\rm{P}}}}}}{V}\times \frac{{{Chla}}^{{B}_{{{{\rm{P}}}}}}}{{{M}_{{{{\rm{P}}}}}}^{{B}_{{{{\rm{P}}}}}}+{{Chla}}^{{B}_{{{{\rm{P}}}}}}}$$

5 $${N}_{{{{\rm{DE}}}}}={S}_{{{{\rm{N}}}}}\times {{{\rm{TN}}}}$$

6 $${P}_{{{{\rm{DE}}}}}={S}_{{{{\rm{P}}}}}\times {{{\rm{TP}}}}$$

The solution to the nutrient budget model (Eqs. (7) and (8)) was slightly modified (Supplementary Note 12). Although mass balance equations are described as first order differential equations with time as the independent variable, the part of the solution function that contains time can be replaced by the parameter *θ*_N_ or *θ*_P_. The parameters represent shocks caused by time and natural loss. Wu et al^3^. set this parameter to 0 considering only one annual data NLA2012. We improved this parameter to a normally distributed random variable and searched for suitable means and variances through model operation. Such an improvement by incorporating shocks in models significantly benefits capture of the lake nutrient evolution over time (Supplementary Note 12 & Supplementary Table S8).

7 $${{{\rm{TN}}}}={\theta }_{{{{\rm{N}}}}}+\frac{\frac{{L}_{{{{\rm{N}}}}}}{V}\,+{N}_{{{{\rm{EN}}}}}}{h+{S}_{{{{\rm{N}}}}}}$$

8 $${{{\rm{TP}}}}={\theta }_{{{{\rm{P}}}}}+\frac{\frac{{L}_{{{{\rm{P}}}}}}{V}\,+{P}_{{{{\rm{EN}}}}}}{h+{S}_{{{{\rm{P}}}}}}$$

We followed the equations to estimate the nutrient loading (*L*_N_ and *L*_P_) for each lake from *f*_NANI_ and *f*_NAPI_ (Eqs. (9) and (10)) using the same hierarchical structure as follows.

9 $${L}_{{{{\rm{N}}}}}=\exp (\,{\theta }_{1}^{{{{\rm{N}}}}}\times {f}_{{{{\rm{NANI}}}}}+{\theta }_{2}^{{{{\rm{N}}}}}\times {{LU}}_{{{{\rm{forest}}}}}+{\theta }_{3}^{{{{\rm{N}}}}}\times {{LU}}_{{{{\rm{wetland}}}}}+{\theta }_{4}^{{{{\rm{N}}}}})$$

10 $${L}_{{{{\rm{P}}}}}=\exp ({\theta }_{1}^{{{{\rm{P}}}}}\times {f}_{{{{\rm{NAPI}}}}}+{\theta }_{2}^{{{{\rm{P}}}}}\times {{LU}}_{{{{\rm{forest}}}}}+{\theta }_{3}^{{{{\rm{P}}}}}\times {{LU}}_{{{{\rm{wetland}}}}}+{\theta }_{4}^{{{{\rm{P}}}}})\,$$

We followed the Eqs. (11) to (14) to estimate the N (P) inflow and outflow.

11 $${{{\rm{Inflow}}}}\;\;{{{\rm{N}}}}=\frac{{L}_{{{{\rm{N}}}}}}{V}$$

12 $${{{\rm{Inflow}}}}\;\;{{{\rm{P}}}}=\frac{{L}_{{{{\rm{P}}}}}}{V}$$

13 $${{{\rm{Outflow}}}}\;\;{{{\rm{N}}}}=h\times {{TN}}_{{{{\rm{post}}}}-{{{\rm{mean}}}}}\,$$

14 $${{{\rm{Outflow}}}}\;\;{{{\rm{P}}}}=h\times {{TP}}_{{{{\rm{post}}}}-{{{\rm{mean}}}}}$$

where *L*_N_ is the external loading input of N (g·day^–1^ N); *L*_P_ is the external loading input of P (g·day^–1^ P); *S*_P_ is the rate of in-lake P depletion (day^–1^); *h* is the ratio of discharge rate to volume (day^–1^); *V* is the lake volume (m^3^); The *θ*_1_^P^, *θ*_2_^P^, *θ*_3_^P^ and *θ*_4_^P^ are parameters to estimate the external loading of TP. The *θ*_1_^N^, *θ*_2_^N^, *θ*_3_^N^ and *θ*_4_^N^ are parameters to estimate the external loading of TN. *LU*_forest_ is the percentage of the basin area classified as forest (%), and *LU*_wetland_ is the percentage of the catchment area classified as wetland (%). *TN*_post-mean_ is the mean value of the posterior distribution of TN (g N); TP_post-mean_ is the mean value of the posterior distribution of TP (g P).

### Bayesian inference

Bayesian methods have been widely used to identify and estimate lake eutrophication at the regional scale^51,52^. We adopted a Bayesian hierarchical framework to distinguish and identify the spatial complexity since lakes were categorized into ten regions based on USEPA Level III and IV Ecoregions classification benchmark. We classified parameters as 10 classes, assuming lakes in the same class share the same internal nutrient cycling coefficients, which means lakes in the same class share the similar patterns of nutrient cycling processes. These ten sets of parameters share identical initial values (Supplementary Tables S11-S13). However, due to the differences among the ten sets of data, the posterior distributions of the parameters for these ten sets are distinct. The reliability of this approach lies in the ability of the Bayesian iterative method to find the most suitable parameters for each category. We run Julia program for parameter inference in cmdStan. Hamilton Monte Carlo method was applied to get samples of each parameter due to its faster sampling. This method provides samples from the posterior distribution of the parameters, giving a complete picture of the uncertainty by showing the range of plausible values along with their associated probabilities (Supplementary Table S14). The mean value of samples for each parameter was used as posterior estimation. Our improvements are mainly in the better selection of prior distribution, since we make full use of USL2012 data as prior data.

### Reporting summary

Further information on research design is available in the Nature Portfolio Reporting Summary linked to this article.

## Supplementary information

Supplementary Information

Reporting Summary

Transparent Peer Review file

## Source data

Source Data

## Data Availability

The input data, including the USL dataset, the *f*_NANI_ & *f*_NAPI_ data, and INCI data, have been deposited in the Figshare database (10.6084/m9.figshare.31259455). Data files used to derive the reported outputs and figures are available at Figshare (10.6084/m9.figshare.32311506). Source data are provided with this paper.

## Code availability

The key codes to reproduce the study are available at Figshare (10.6084/m9.figshare.32297847).
