# Supplementary material for: Preferential nitrogen retention characterizes current eutrophication in United States lakes
Source: Nat Commun. 2026 Jul 9;17:8485. doi: 10.1038/s41467-026-75318-9 (PMC13478381; doi:10.1038/s41467-026-75318-9)
Supplement: Supplementary file 1 [file 41467_2026_75318_MOESM1_ESM.pdf]

Supplementary Information File

Ran Zhou[1], Josep Peñuelas[2], Jordi Sardans[2], Yindong Tong[3], Jinlong Zhang[1], John A. Dearing[4], Meng Liu[1], Zisong Chen[1], Xinyi Wu[1], Shu Tao[1], Xilong Wang[1]*

[1] College of Urban and Environmental Sciences, Peking University, Beijing, China.

[2] CSIC, Global Ecology Unit CREAF-CSIC-UAB, Bellaterra, Spain.

[3] School of Environmental Science and Engineering, Tianjin University, Tianjin, China.

[4] Palaeoecological Laboratory, Geography and Environment, University of Southampton, Southampton SO17 1BJ, UK.

*Corresponding author

E-mail: xilong@pku.edu.cn (X. Wang)

Supplementary Note 1: In-lake nutrient enrichment and depletion

In this study, to obtain the status of net nutrient retention and preferential nutrient retention, the fluxes of internal cycling processes (N_{EN}, N_{DE}, P_{EN}, and P_{DE}) were estimated for the 121 representative lakes in the United States.

-Calculation details

The calculations of N_{EN} and P_{EN} are based on equations (3) - (4) in Methods section. In the traditional model, multiple parameters of each lake are estimated through statistical methods, including: A_N ($g \cdot d^{-1}$), A_P ($g \cdot d^{-1}$), M_N ($mg \cdot m^{-3}$), M_P ($mg \cdot m^{-3}$), B_N and B_P. By calculating the mean value of each parameter for each lake and combining it with the measured chlorophyll a concentration (Chla) and water volume (V) of that lake, N_{EN} and P_{EN} ($g \cdot m^{-3} \cdot d^{-1}$) are calculated.

The calculations of N_{DE} and P_{DE} are based on equations (5) - (6) in Methods section. In the traditional model, two parameters for each lake are estimated through statistical methods, including: S_N(d^{-1}) and S_P(d^{-1}). By using mean values of the estimated parameters and combining them with the measured total nitrogen (TN) and total phosphorus (TP), N_{DE} and P_{DE} are calculated.

The traditional method has certain limitations. Firstly, the uncertainty of parameter estimation is challenging. Specifically, each parameter is estimated using statistical models or empirical formulas, but it lacks a comprehensive and standardized reference framework. Secondly, the traditional method often fails to fully utilize all information present in the original data. Especially when dealing with multiple ecological variables or complex ecosystems, important correlations and underlying patterns in the data are often overlooked.

In the Bayesian hierarchical model, not only various parameters of a lake are regarded as random variables, with each parameter following its own normal distribution N (μ, σ^2), but also the mean value (μ) and standard deviation (σ) of each parameter are considered as random variables following a normal distribution. Additionally, through continuous stratification, 10 groups of sub-parameters are constructed for each parameter based on 10 ecological regions (USEPA Level III and IV Ecoregions classification benchmark, Supplementary Fig. S1). The parameters within the same ecological region are linked by formulas to achieve maximum utilization of the correlations and inherent regularities of the data. The posterior distribution reflects the update of our understanding of these parameters based on the observed data.

In this study, we calculate the post-mean of the posterior distribution of these parameters in the ecological region to which the lake belongs, across different ecological regions, and combine it with the measured chlorophyll a concentration (Chla), total nitrogen (TN), total phosphorus (TP) and water volume (V) of that lake. N_{EN}, P_{EN}, N_{DE} and P_{DE} are thus calculated using the following

equations (1)-(4).

$$N_{EN} = \frac{A_{N\ post-mean}}{V} \times \frac{Chla^{B_{N\ post-mean}}}{M_{N\ post-mean}{}^{B_{N\ post-mean}} + Chla^{B_{N\ post-mean}}} \quad (1)$$

$$P_{EN} = \frac{A_{P\ post-mean}}{V} \times \frac{Chla^{B_{P\ post-mean}}}{M_{P\ post-mean}{}^{B_{P\ post-mean}} + Chla^{B_{P\ post-mean}}} \quad (2)$$

$$N_{DE} = S_{N\ post-mean} \times TN \quad (3)$$

$$P_{DE} = S_{P\ post-mean} \times TP \quad (4)$$

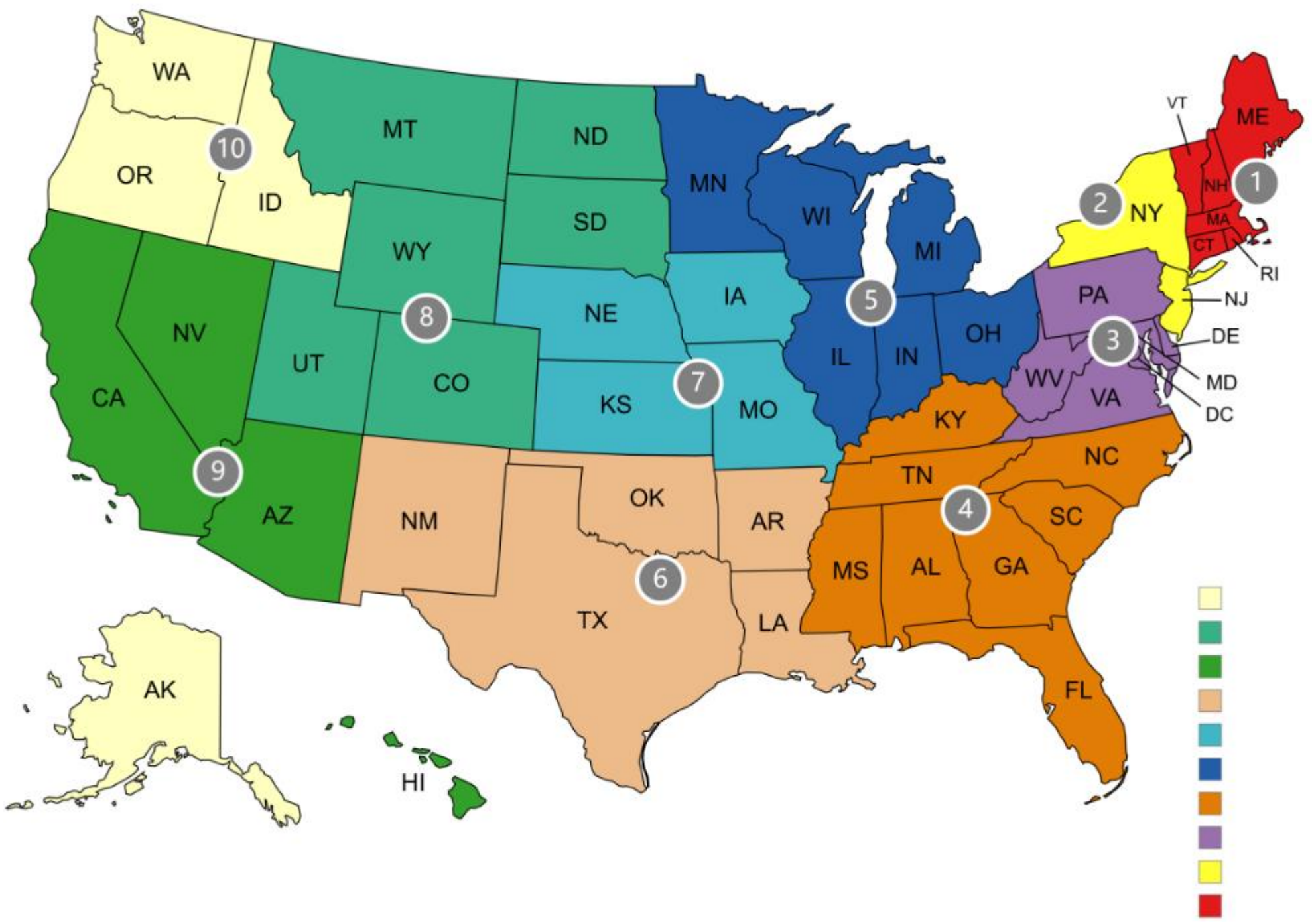

Figure S1 USEPA Level III and IV Ecoregions classification benchmark. An ecoregion is a geographic unit defined by the significant similarity of the combined characteristics of ecosystem structure, functions, and their environmental elements. The strict classification criterion for ecoregions is to identify them by analyzing the patterns and compositions of biotic and abiotic phenomena including geology, landforms, soils, vegetation, climate, land use, wildlife, and hydrology. The base map was generated using MapChart and is licensed under a Creative Commons Attribution-ShareAlike 4.0 International License (https://creativecommons.org/licenses/by-sa/4.0/). The map was further edited by the authors.

-Results of overall pattern and ecoregion pattern

From 2012 to 2022, the mean and median values of N_{EN} exhibited a continuous downward

trend (Figs. S2-S4). For P_{EN}, both the mean and median values decreased from 2012 to 2017 but showed a slight increase from 2017 to 2022 (Figs. S5-S7). Detailed quantitative descriptions are provided in each supplementary figure. It is worth noting that the processes of enrichment and depletion do not provide substantial insight; therefore, they are not described in the main text. Instead, we focused on the more informative concepts of net retention and preferential retention as the central themes of our discussion.

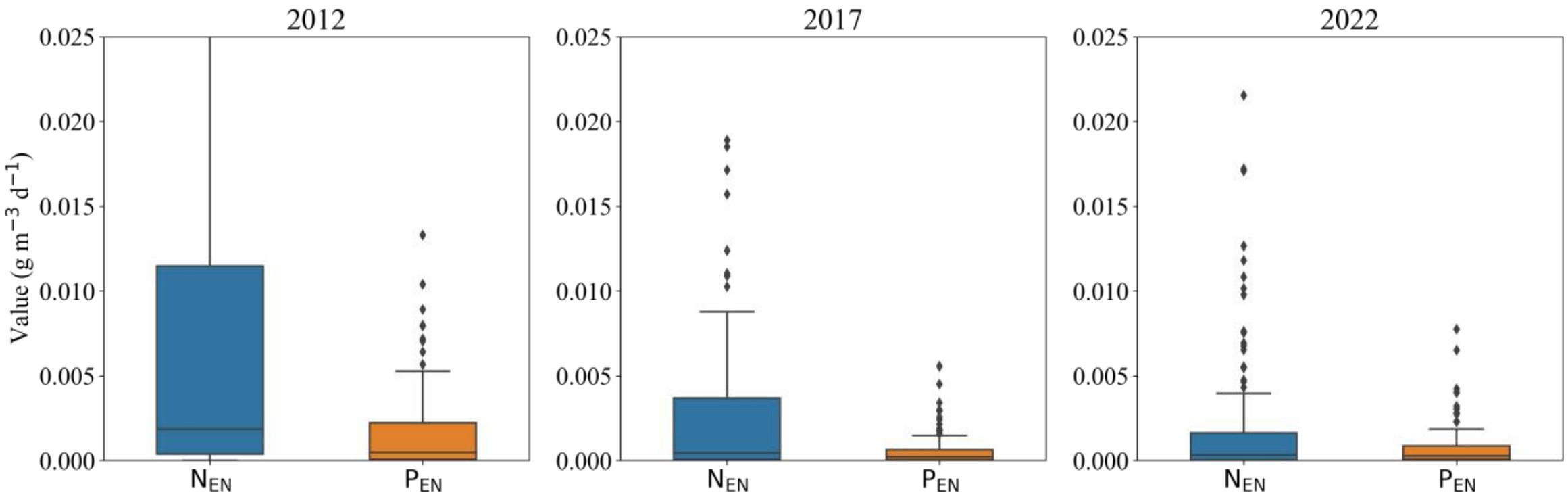

Figure S2 The quantitative statistical distribution of N_{EN} and P_{EN} in the three sampling years. Source data are provided as a Source Data file.

As shown in Fig. S2, the magnitude between N_{EN} and P_{EN} varied within a range of 5 to 10 times. In 2012, the mean value of N_{EN} was 0.0088 $g \cdot m^{-3} \cdot d^{-1}$, substantially higher than the mean P_{EN} value of 0.0012 $g \cdot m^{-3} \cdot d^{-1}$. Moreover, the standard deviation of N_{EN} (0.015 $g \cdot m^{-3} \cdot d^{-1}$) was significantly larger than that of P_{EN} (0.0023 $g \cdot m^{-3} \cdot d^{-1}$), suggesting a greater degree of dispersion in the N_{EN} data with fluctuations across a broader spectrum. The minimum and the maximum values of N_{EN} were 0.000003 $g \cdot m^{-3} \cdot d^{-1}$ and 0.0757 $g \cdot m^{-3} \cdot d^{-1}$, respectively, which were significantly higher than the corresponding values of P_{EN} (5.79×10^{-8} $g \cdot m^{-3} \cdot d^{-1}$ and 0.013 $g \cdot m^{-3} \cdot d^{-1}$).

A similar trend persisted in 2017, with the mean N_{EN} value standing at 0.0048 $g \cdot m^{-3} \cdot d^{-1}$, still higher than the P_{EN} value of 0.00057 $g \cdot m^{-3} \cdot d^{-1}$, indicating that the average in-lake enrichment level of N remained higher than that of P in 2017. The standard deviation of N_{EN} continued to surpass that of P_{EN}, and a notable gap persisted between the ranges of N_{EN} and P_{EN}. By 2022, the mean N_{EN} value was 0.0032 $g \cdot m^{-3} \cdot d^{-1}$, still above the mean P_{EN} value of 0.00071 $g \cdot m^{-3} \cdot d^{-1}$. However, both N and P enrichment exhibited a weakening trend, and the difference between them notably diminished.

(a)

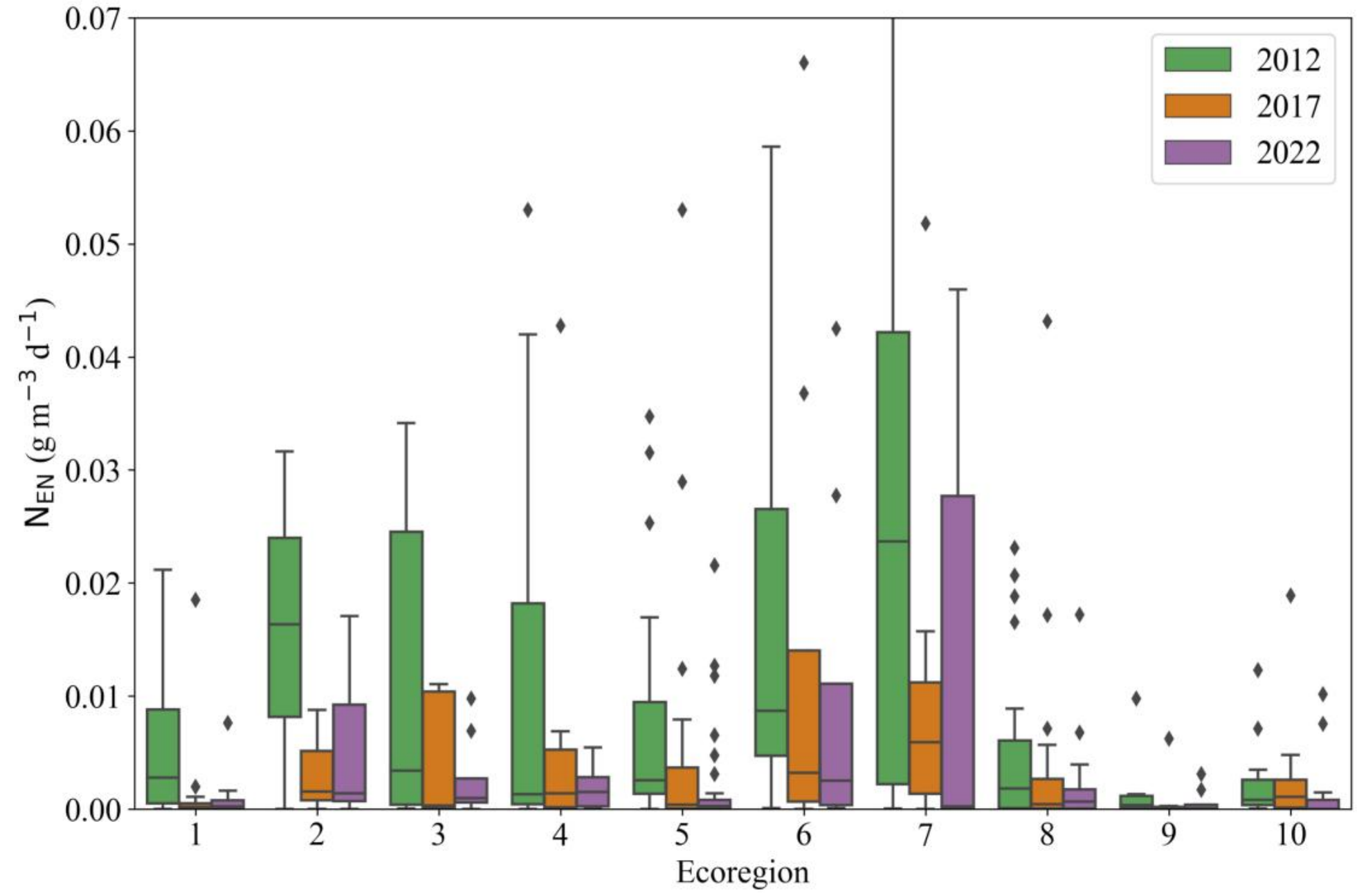

(b)

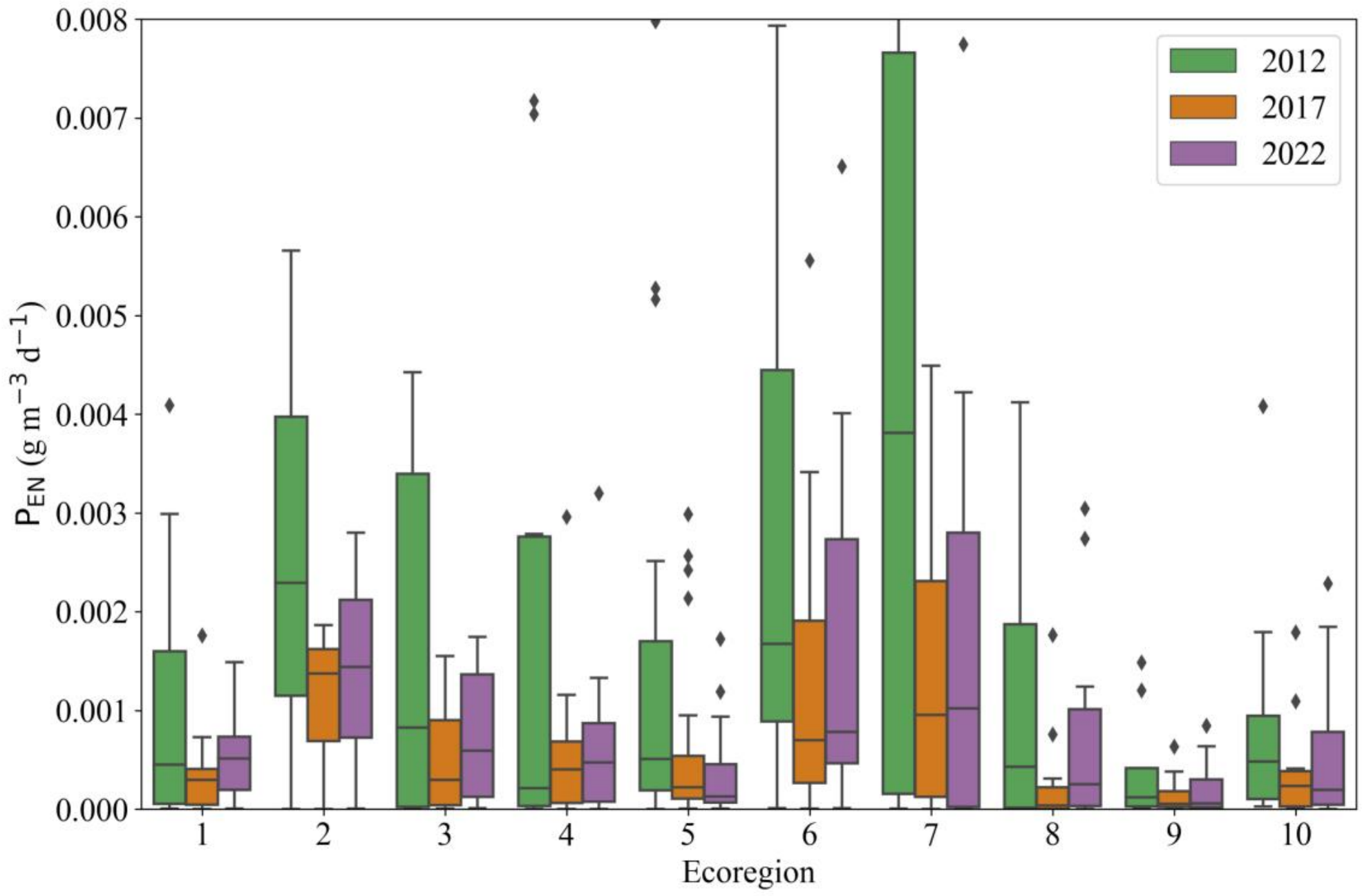

Figure S3 The quantitative statistical distribution of (a) N_{EN} and (b) P_{EN} in ten ecoregions in the three sampling years. Source data are provided as a Source Data file.

Fig. S3(a) reveals that the mean and median N_{EN} values for the majority of ecoregions remain relatively low across different years, primarily ranging from 0.00 to 0.03 $g \cdot m^{-3} \cdot d^{-1}$, suggesting limited overall in-lake N enrichment. For most ecoregions, both the mean and median N_{EN} values

exhibit a notable declining trend from 2012 to 2022, with the range gradually contracting. In 2012, ecoregion 7 displayed a higher mean N_{EN} value (0.03 $g{\cdot}m^{-3}{\cdot}d^{-1}$) compared to other ecoregions, and this value persisted at a relatively elevated level over the three-year period, implying that certain unique factors could influence in-lake N enrichment in this specific ecoregion.

Fig. S3(b) shows that the mean and median P_{EN} values for most ecoregions fluctuated between 2012 and 2022, with the majority showing a pattern of initial decrease followed by an increase. In 2012, ecoregions 6 and 7 exhibited relatively higher mean P_{EN} values, at 0.0036 $g{\cdot}m^{-3}{\cdot}d^{-1}$ and 0.0043 $g{\cdot}m^{-3}{\cdot}d^{-1}$, respectively, suggesting that some specific factors could more significantly influence in-lake P enrichment in these two ecoregions compared to others. The substantial differences in standard deviations across years among different ecoregions highlight significant variations in the spatial distribution uniformity of the indicators among the ecoregions. In 2012, ecoregion 4 had a comparatively large P_{EN} standard deviation of 0.0029 $g{\cdot}m^{-3}{\cdot}d^{-1}$, while ecoregion 1 had a relatively small standard deviation of 0.00043 $g{\cdot}m^{-3}{\cdot}d^{-1}$ in 2017.

When simply categorized by geographic regions (east, central, and west), a remarkable spatial differentiation in N_{EN} and P_{EN} becomes evident among the ecoregions:

- Ecoregions 9 and 10 consistently exhibited low levels of N_{EN} and P_{EN}; both are located in the far western United States.

- Ecoregions 6 and 7 consistently displayed relatively high N_{EN} and P_{EN} levels; both are situated in the central plains.

- Ecoregions 3 and 4, which showed similar declines in N_{EN} and P_{EN}; are both nestled in the Appalachian Mountains in the eastern region.

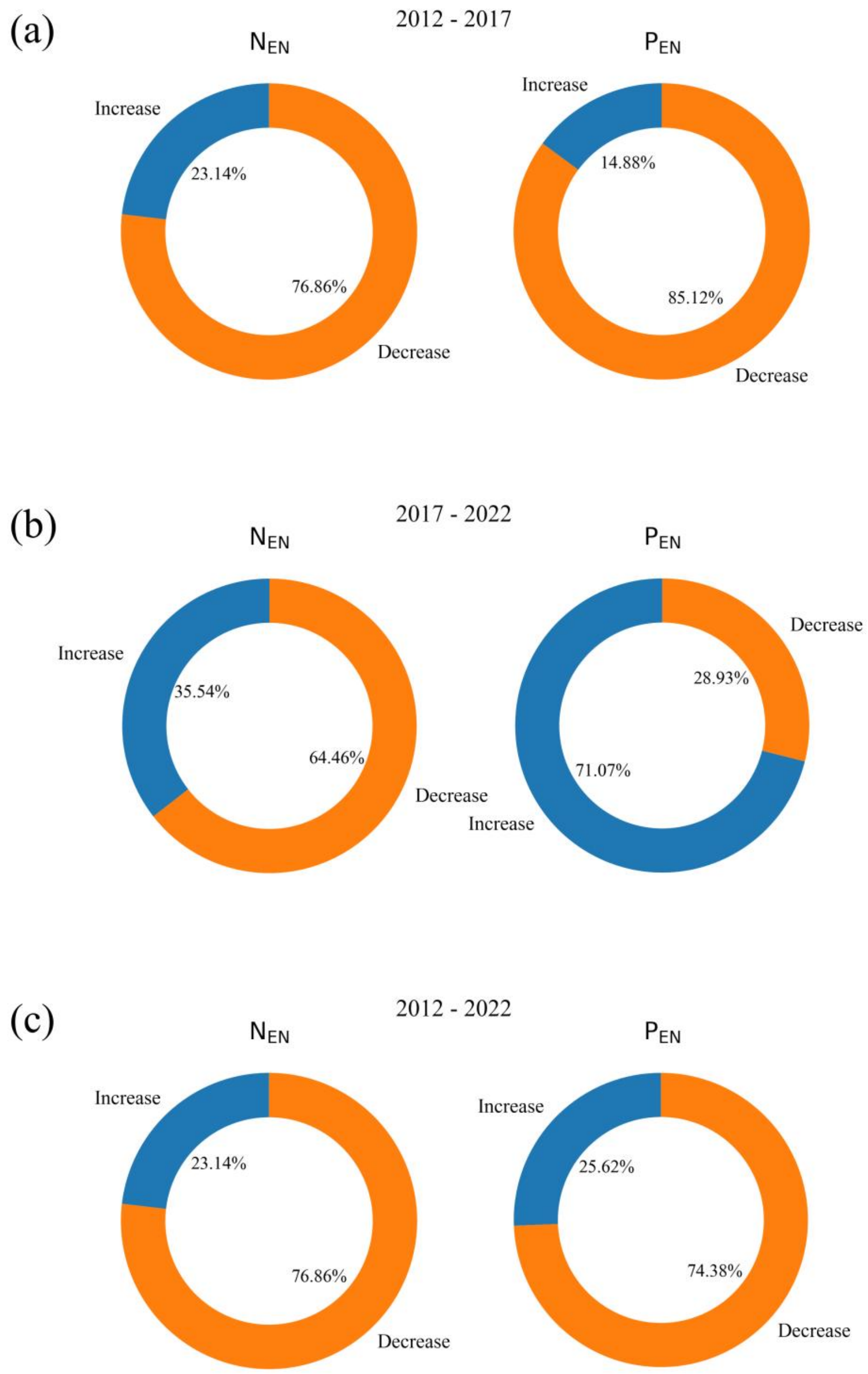

Figure S4 The proportions of lakes with increasing or decreasing trend of N_{EN} and P_{EN} from (a) 2012 to 2017, (b) 2017 to 2022, (c) 2012 to 2022. Source data are provided as a Source Data file.

In comparison with the quantitative analysis, the qualitative analysis on the proportion of lakes experiencing growth and decline in N_{EN} and P_{EN} between 2012 and 2022 shows higher fault tolerant. This approach better reflects the overall variation trends for the 121 lakes.

As displayed in Fig. S4(c), lakes with increasing N_{EN} accounted for only 23.14%, while those with decreasing N_{EN} accounted for 76.86% from 2012 to 2022. This indicates that the overall N_{EN} of lakes in the United States exhibited a decreasing trend. During 2012-2022, the proportion of lakes with increasing P_{EN} was 25.62%, which was significantly lower than that of lakes with

decreasing P_{EN}, but slightly higher than the proportion of lakes with increasing N_{EN}. This suggests that the overall P_{EN} of lakes in the United States demonstrated a decreasing trend, and the decline in N_{EN} and P_{EN} was synergistic.

It is noteworthy that, as shown in Fig. S4(a,b), the qualitative analysis revealing the proportion of lakes with increasing/decreasing trends in N_{EN} and P_{EN} over each 5-year stage aligns perfectly with the quantitative analysis of these two parameters based on their mean and median values. This suggests that the 121 lakes under our study possess good representativeness in terms of in-lake enrichment.

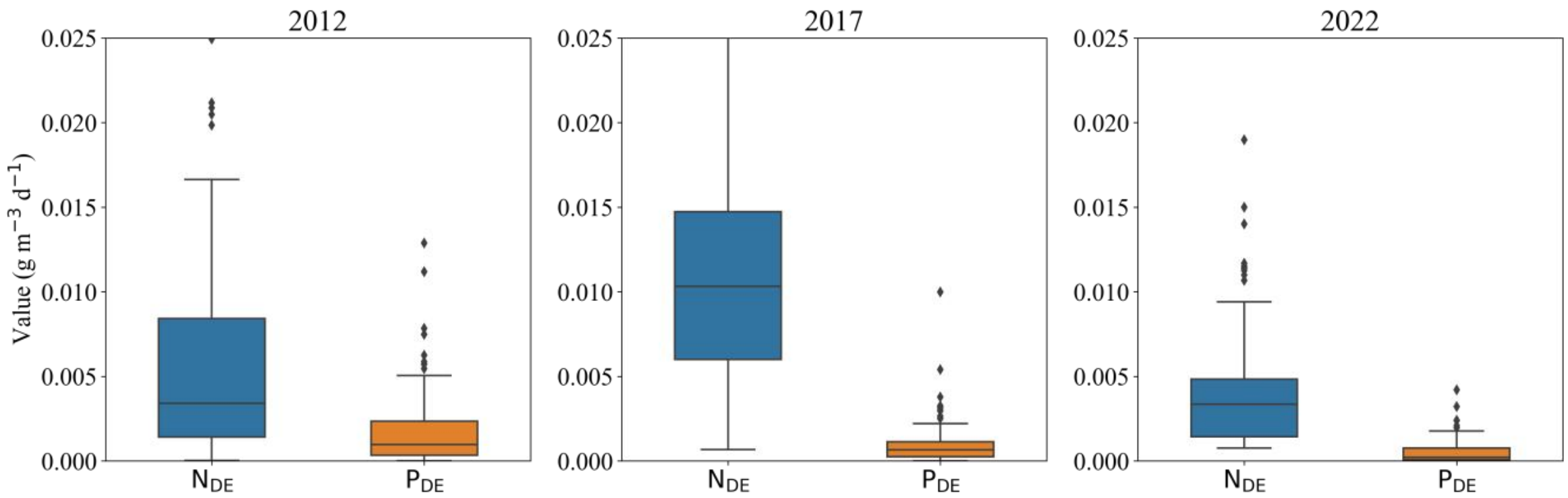

Figure S5 The quantitative statistical distribution of N_{DE} and P_{DE} in the three sampling years. Source data are provided as a Source Data file.

Fig. S5 presents the quantitative statistical distributions of N_{DE} and P_{DE} across the three sampling years. From 2012 to 2022, the overall in-lake N depletion was higher and its fluctuation was more significant than those of P, possibly due to the denitrification process. In 2012, the mean value of N_{DE} was 0.0059 $g \cdot m^{-3} \cdot d^{-1}$, surpassing the P_{DE} value of 0.0017 $g \cdot m^{-3} \cdot d^{-1}$, and N_{DE} exhibited a larger standard deviation. In 2017, the mean value of N_{DE} reached 0.011 $g \cdot m^{-3} \cdot d^{-1}$, the highest among the three years, while the P_{DE} mean value remained the same as that in 2012. By 2022, and the mean values of both N_{DE} and P_{DE} decreased compared to those in 2017. Notably, the standard deviations of N_{DE} and P_{DE} in 2022 were much lower than those in 2012 and 2017, indicating a more balanced distribution of N_{DE} and P_{DE}.

(a)

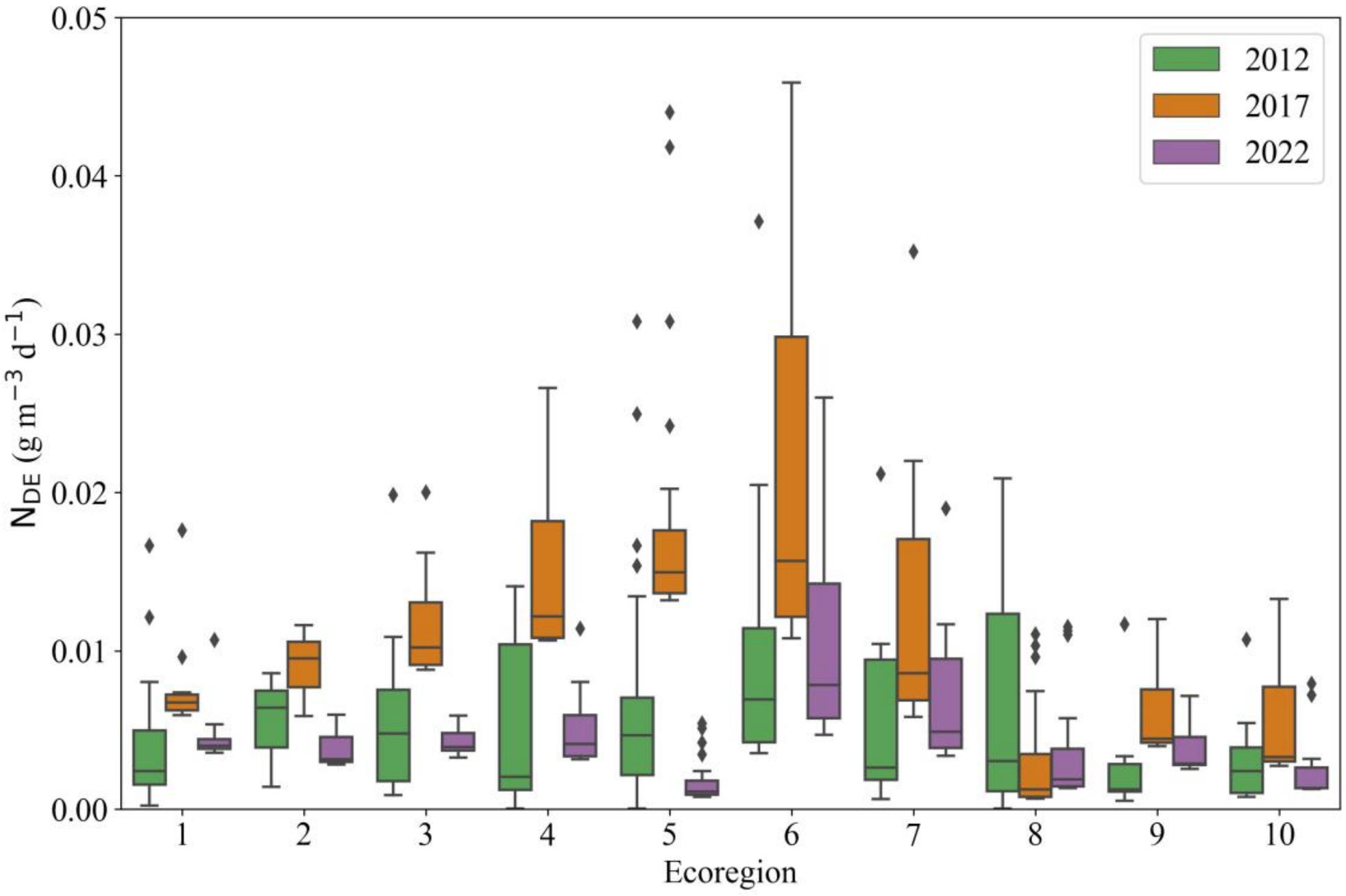

(b)

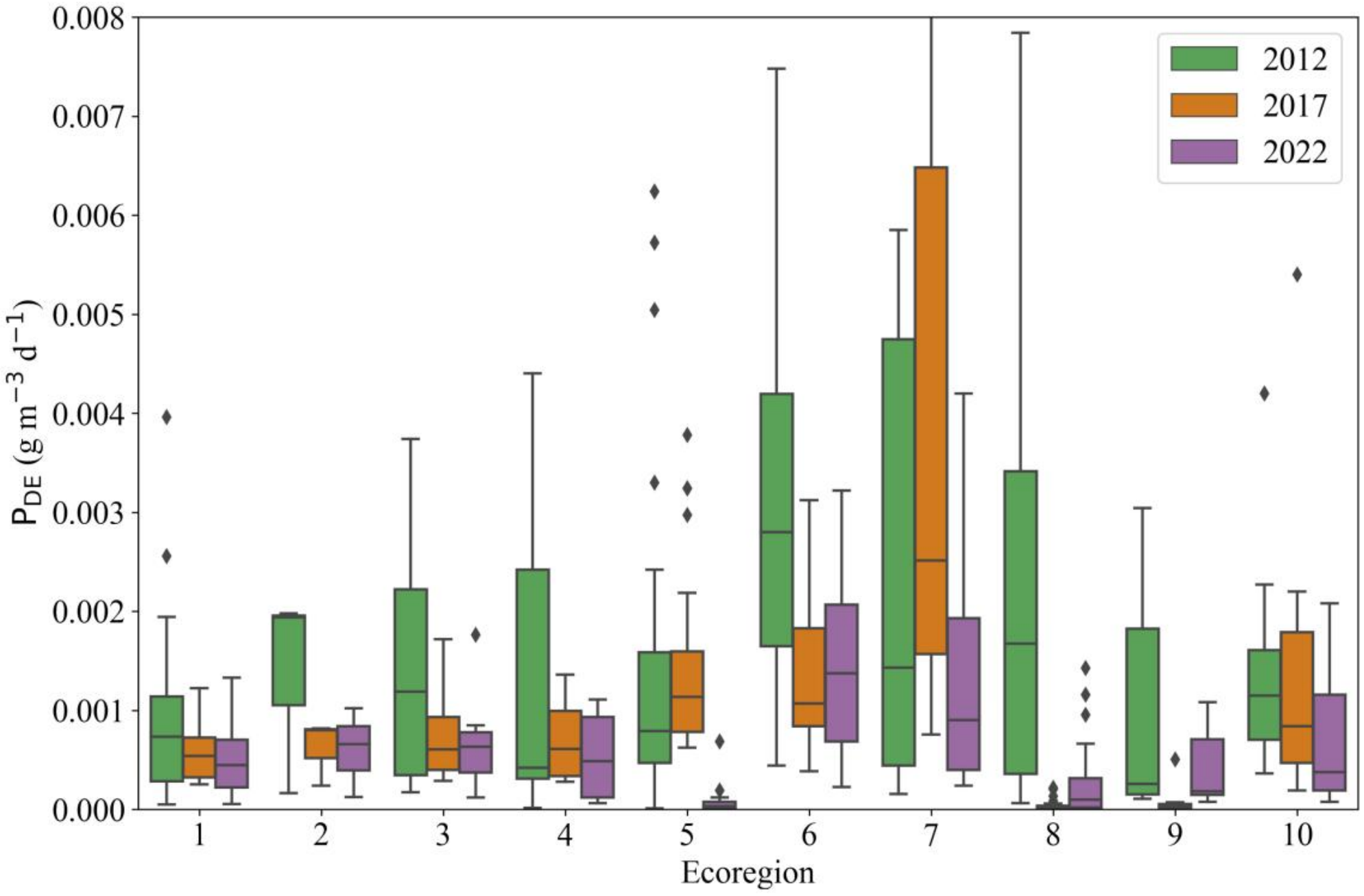

Figure S6 The quantitative statistical distribution of (a) N_{DE} and (b) P_{DE} in 10 ecoregions in the three sampling years. Source data are provided as a Source Data file.

Fig. S6(a) shows that the mean N_{DE} values for most ecoregions fluctuated across different years. For example, the mean N_{DE} value for Ecoregion 1 was 0.008 $g \cdot m^{-3} \cdot d^{-1}$ in 2017, higher than those in 2012 and 2022. The mean N_{DE} value of Ecoregion 6 increased from 0.006 $g \cdot m^{-3} \cdot d^{-1}$ in

2012 to 0.015 $g \cdot m^{-3} \cdot d^{-1}$ in 2017, and then decreased to 0.008 $g \cdot m^{-3} \cdot d^{-1}$ in 2022. Significant differences in the mean N_{DE} values among different ecoregions are evident, with Ecoregion 6 being the most prominent. In each year, the maximum N_{DE} value of Ecoregion 6 was 0.02-0.03 $g \cdot m^{-3} \cdot d^{-1}$ higher on average than that of other ecoregions. Regarding variation trends, the N_{DE} of most ecoregions exhibited a pattern of initial increasing followed by decrease, and the range (maximum minus minimum value) displayed a decreasing trend, which was completely consistent with the overall trend.

Fig. S6(b) indicates that, in most ecoregions, P_{DE} values across different years were generally low, with both mean and median values mostly not exceeding 0.003 $g \cdot m^{-3} \cdot d^{-1}$, nearly an order of magnitude lower than N_{DE}. From 2012 to 2022, the maximum values and ranges (maximum minus minimum values) of P_{DE} in most ecoregions decreased. Unlike the trend of N_{DE}, P_{DE} in most ecoregions showed a consistent downward trend.

When simply categorized by geographic regions, significant spatial differentiation characteristics of N_{DE} and P_{DE} become evident among ecoregions:

- Ecoregions 9 and 10 consistently maintained low N_{DE} and P_{DE} levels; both are located in the far western United States.

- Ecoregions 6 and 7 consistently demonstrated relatively high N_{DE} and P_{DE} levels; both are situated in the central plains.

- Ecoregions 3 and 4, which exhibited similar changing trends in N_{DE} and P_{DE}, are both nestled in the Appalachian Mountains in the eastern region.

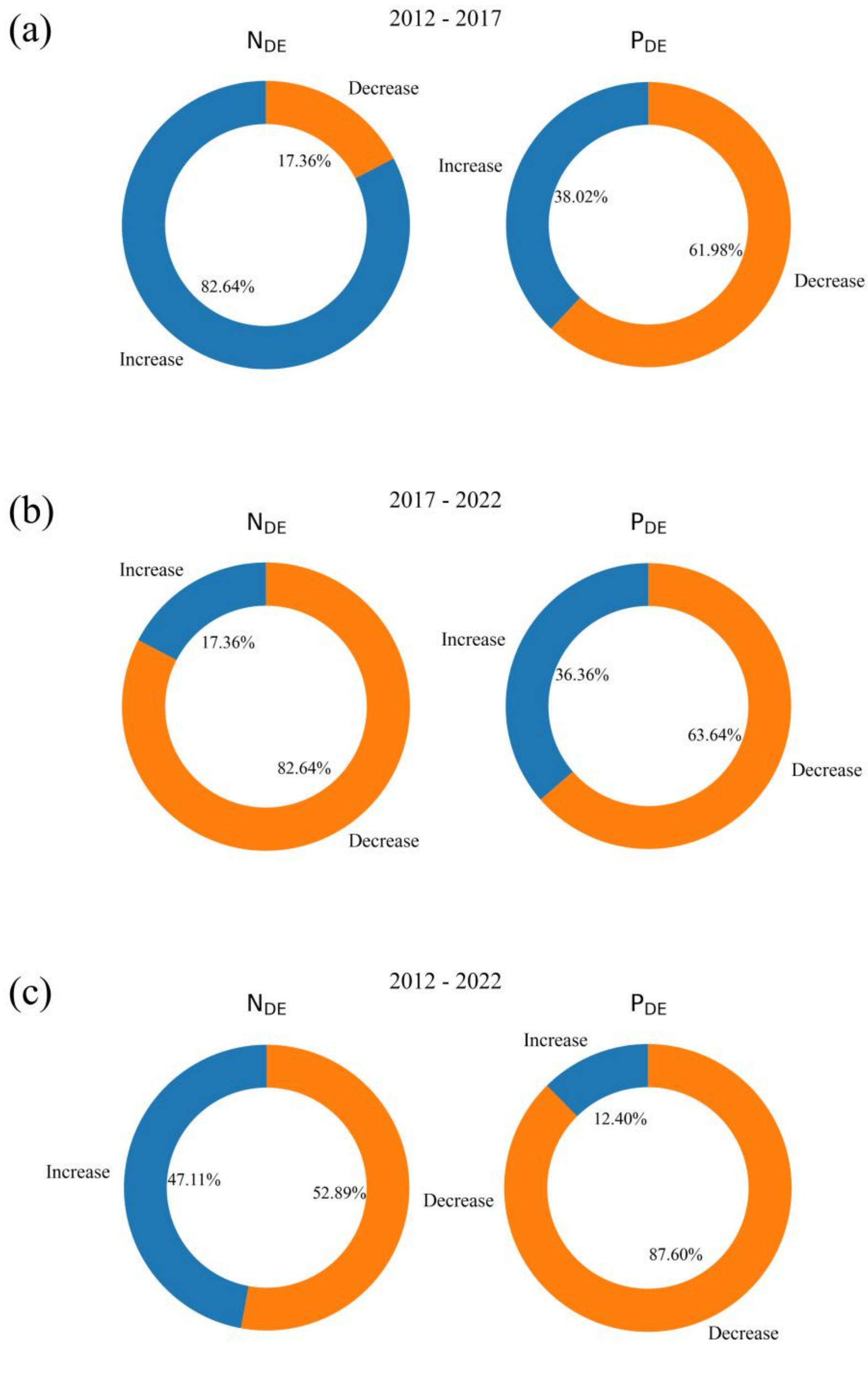

Figure S7 (a) The changing trends of N_{DE} and P_{DE}. The proportions of lakes with increasing or decreasing N_{DE} and P_{DE} from (b) 2012 to 2017, (c) 2017 to 2022, (d) 2012 to 2022. Source data are provided as a Source Data file.

Fig. S7 illustrates that from 2012 to 2022, lakes with increasing N_{DE} accounted for 47.11%, while those with decreasing N_{DE} accounted for 52.89%. The changes in the proportion of lakes in each stage are consistent with the trend of the mean N_{DE} value for the 121 lakes, which exhibit a pattern of initial increase followed by decrease. This indicates that the changes in N_{DE} of U.S. lakes were not polarized (with half of the lakes showing continuous N_{DE} increase and the other half showing continuous decrease), but rather a large-scale trend of first increasing and then decreasing.

From 2012 to 2022, the proportion of lakes with increasing P_{DE} was only 12.40%, far lower than that of lakes with decreasing P_{DE} and completely different from the changes in N_{DE}. This suggests that the overall P_{DE} of U.S. lakes displayed a decreasing trend, and the changes in P_{DE} and N_{DE} were independent of each other.

Supplementary Note 2: Net retention and preferential retention

-Calculation details

In this study, three new binary classification parameters (N_{net}, P_{net}, and pefer), were introduced for each lake to better characterize its nutrient retention dynamics. The variable N_{net} represents the net N retention status of the lake ($N_{DE} > N_{EN}$: $N_{net} = 1$, $N_{DE} < N_{EN}$: $N_{net} = -1$). The variable P_{net} represents the net P retention status of the lake ($P_{DE} > P_{EN}$: $P_{net} = 1$, $P_{DE} < P_{EN}$: $P_{net} = -1$). The variable, prefer, is adopted to represent the preferential retention situation of the lake ($N_{DE}/N_{EN} > P_{DE}/P_{EN}$: prefer = 1, $N_{DE}/N_{EN} < P_{DE}/P_{EN}$: prefer = -1).

(a)

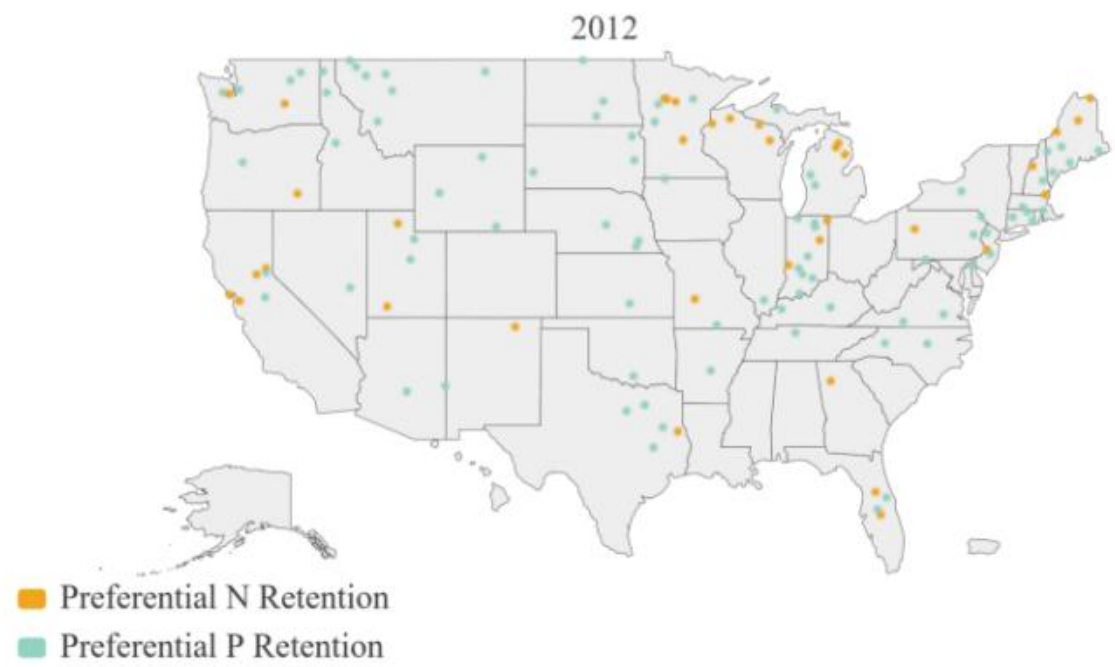

(b)

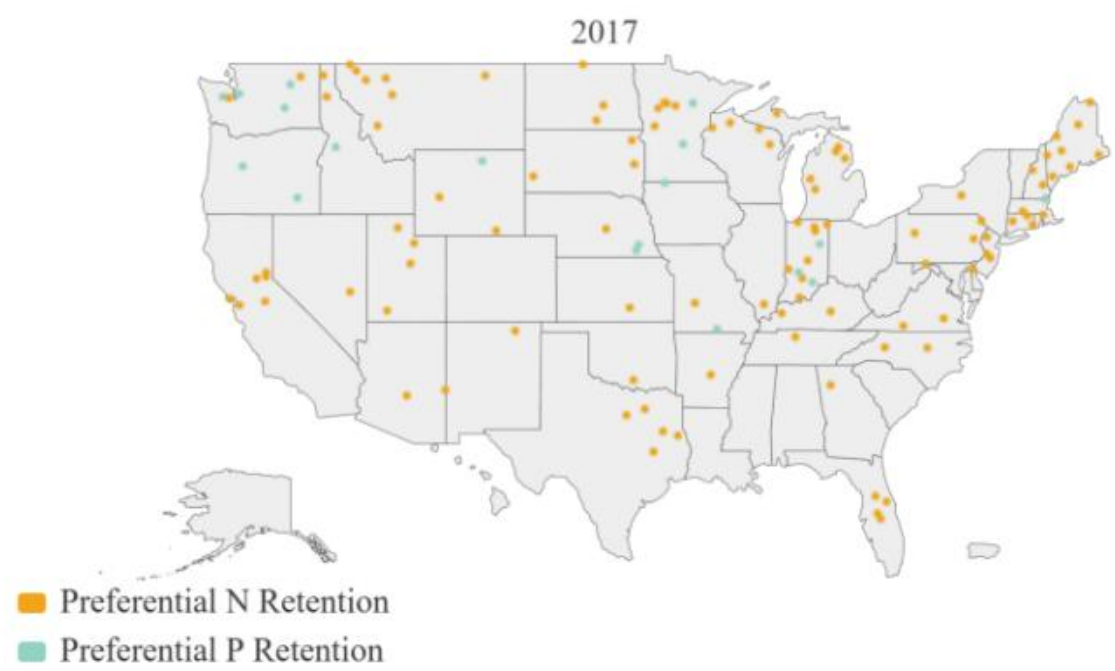

(c)

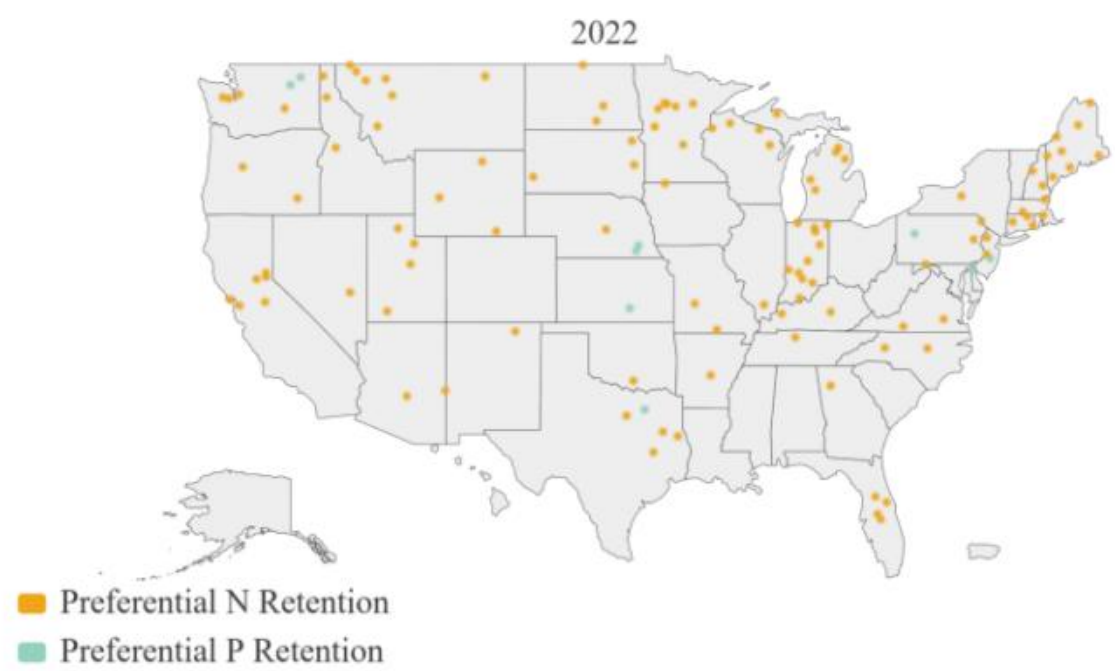

Figure S8 The geographical distribution of preferential nutrient retention. Source data are provided as a Source Data file.

-Results of ecoregion pattern

This study further sheds light on the trend of lakes in each ecological region shifting from preferential of phosphorus (P) retention to nitrogen (N) retention. The trend slope was calculated using linear regression to quantify the intensity of this trend. By taking the year as the independent variable and the proportion of lakes with preferential retention of N as the dependent variable, a linear regression analysis was conducted to obtain the trend slope ($pp \cdot y^{-1}$) for each ecological region. It is important to note that we use "pp" (percentage point) instead of "%" to avoid ambiguity.

For example, if the proportion changes from 10% to 20%, we describe this as a change of 10 pp, rather than 10%. This is because a change of 10% could also mean a change from 10% to 11%. The trend slopes in each ecological region are all greater than 0 (Supplementary Fig. S9), indicating that the proportion of lakes with preferential retention of N in each ecoregion shows a relatively clear upward trend. Furthermore, the larger the trend slope, the more significant the rate of transition from P to N preferential retention in that ecological region would be. This phenomenon highlights a cross-regional consistent shift in the preferential retention from P to N, rather than a localized characteristic.

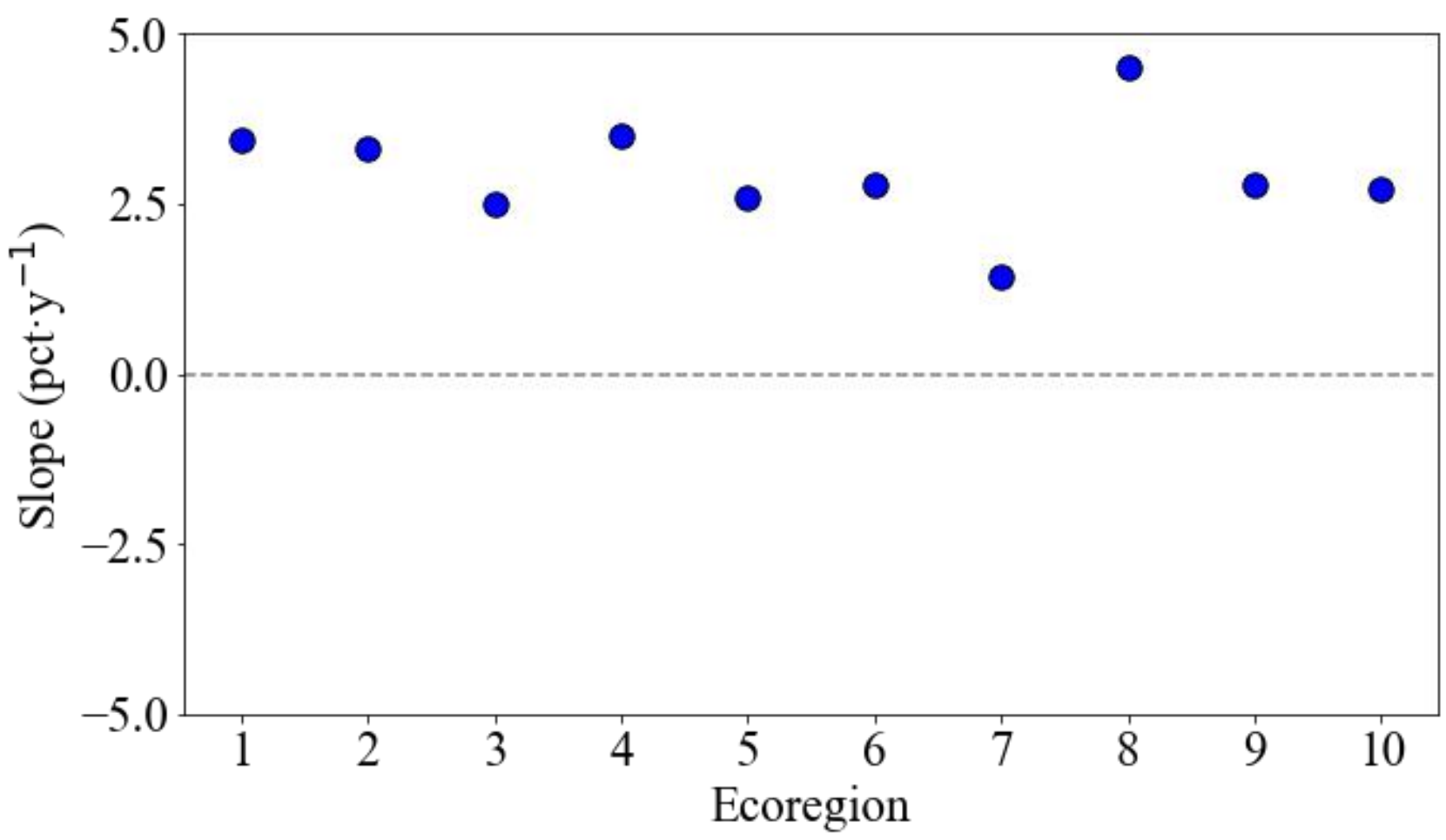

Figure S9 The trend slope of the shift from preferential retention of P to N in each ecoregion. Source data are provided as a Source Data file.

The proportions of net N and P retention in lakes across diverse ecoregions displays a clear distinction, but they all align with the overall distribution pattern and changing trend (Supplementary Fig. S9).

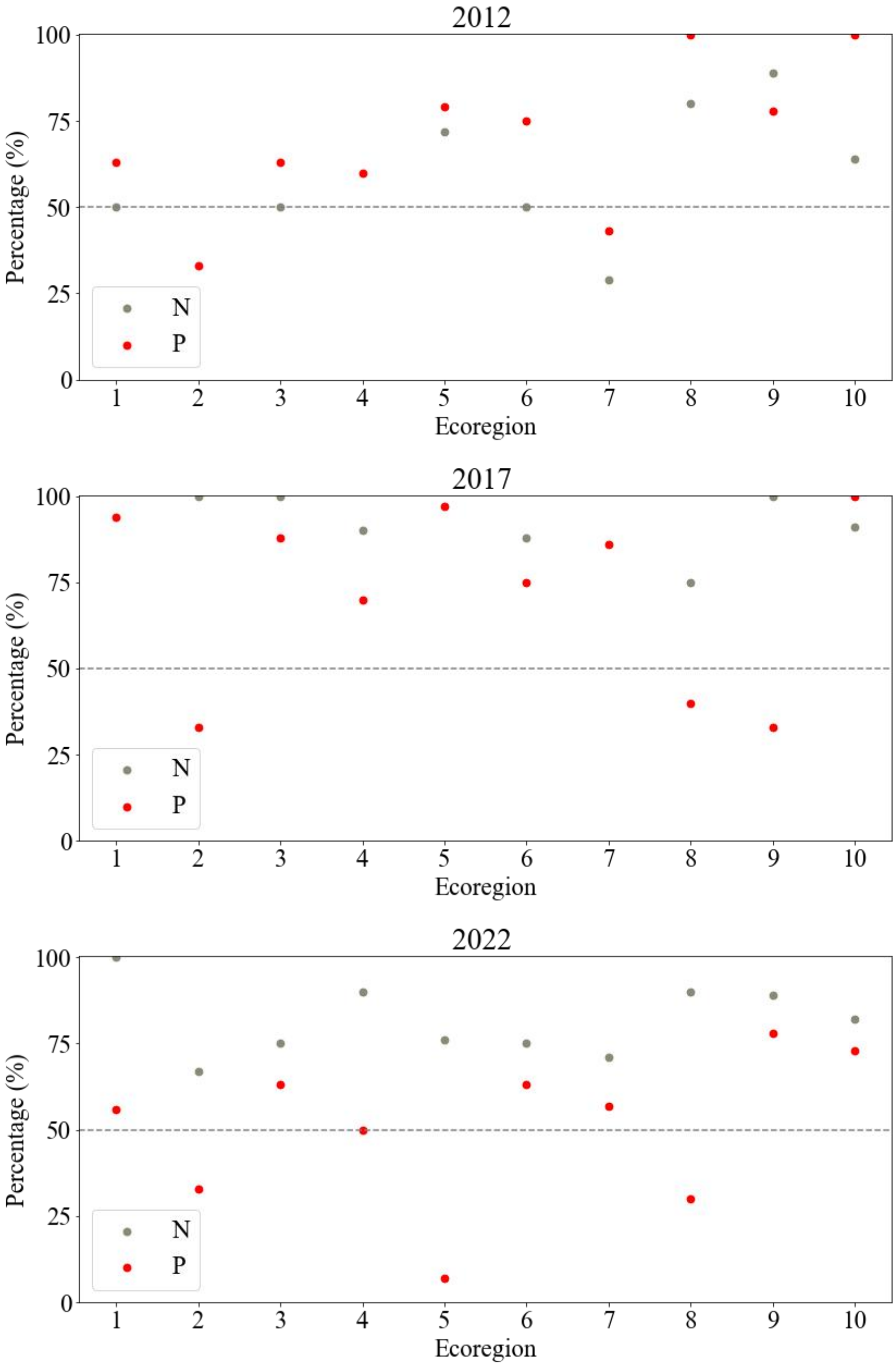

Figure S10 The proportion of lakes with net retention of N (P) in each ecoregion. Source data are provided as a Source Data file.

Supplementary Note 3: Causal inference analysis of the relationship between preferential N retention and net P retention

To further examine whether the observed association between preferential N retention and net P retention is robust to a causal inference framework, we employed a fixed-effects Logit model with lake fixed effects and a post-period time fixed effect (equations 5 & 6). This approach allows us to control for unobserved, time-invariant heterogeneity across lakes (e.g., geomorphology, baseline nutrient cycling characteristics) as well as common temporal shocks affecting all lakes (e.g., regional climate variability or broad management changes). By accounting for both lake-specific and time-specific effects, the model reduces potential confounding arising from stable lake attributes and shared temporal trends, thereby providing a more rigorous assessment of the relationship between preferential N retention and net P retention.

$$\log\left(\frac{P_{i,t}}{1\text{-}P_{i,t}}\right)=\alpha_i+\gamma_t+\beta_1\cdot X_{centered,i,t}+\beta_2\cdot N_{centered,i,t}+\varepsilon_{i,t} \quad (5)$$

$$\gamma_t=\beta_0+\beta_3\cdot year_{2017\text{-}2022,t} \quad (6)$$

Symbols	Explanations
t	Time index, taking values 2012, 2017, and 2022.
$P_{i,t}$	Binary indicator of net phosphorus (P) retention status for lake i in year t, where 1 denotes positive and 0 denotes negative.
$\log(\frac{P_{i,t}}{1\text{-}P_{i,t}})$	Log-odds of net P retention, representing the Logit transformation that maps the binary outcome probability to a continuous scale.
α_i	Lake-specific fixed effect capturing time-invariant characteristics of lake i
γ_t	Common time trend term accounting for shared temporal influences across lakes, approximated by a post-2017 time indicator to control for broad changes affecting the 2017–2022 period
$X_{centered,i,t}$	Within-lake centered indicator of preferential nitrogen (N) retention for lake i in year t, where 1 indicates preferential N retention and 0 indicates preferential P retention.
$N_{centered,i,t}$	Within-lake centered indicator of net nitrogen (N) retention status for lake i in year t, where 1 denotes positive and 0 denotes negative
$year_{2017\_2022,t}$	Time dummy variable, equal to 1 for observations in 2017 and 2022, and 0 for observations in 2012.
β_0	Intercept term
β_1	Coefficient of primary interest, capturing the association between preferential N retention and the likelihood of net P retention

Symbols	Explanations
t	Time index, taking values 2012, 2017, and 2022.
β_2	Coefficient associated with net N retention status, included as a control variable
β_3	Coefficient capturing the common post-2017 time trend.
$\varepsilon_{i,t}$	Unobserved error component implicitly accounted for in the maximum-likelihood estimation of the Logit model

The fixed-effects Logit analysis provides consistent evidence of a negative association between preferential N retention and net P retention (Supplementary Table S1). The estimated coefficient on preferential N retention is −2.54 (SE = 0.41), which is highly statistically significant ($z = -6.21$, $p < 0.001$), with the 95% confidence interval [−3.35, −1.74] excluding zero.

Model diagnostics indicate satisfactory fit and validity, with a McFadden pseudo R^2 exceeding conventional thresholds and no evidence of problematic multicollinearity. Although heteroskedasticity is detected, inference is robust to cluster-robust standard errors. Importantly, the negative effect of preferential N retention remains stable across alternative specifications, including Probit estimation and subsample analyses, and is further supported by placebo tests showing that the estimated effect lies far outside the distribution of coefficients obtained under randomized treatment assignments.

While the parallel trends assumption is not fully satisfied and thus warrants cautious interpretation, the convergence of results across multiple robustness and validity checks supports the conclusion that preferential N retention exerts an inhibitory effect on the probability of net P retention.

Table S1 Model diagnostics and robustness checks for the two-way fixed-effects Logit analysis

Category	Statistic	Value	Interpretation
Model fit	McFadden pseudo R^2	0.259	Indicates good model fit (>0.2)
	Log-likelihood	−100.89	—
	LR statistic	70.67	Model jointly significant
	LR test p-value	<0.001	Reject null of no explanatory power
Predictive ability	AUC	0.815	Strong discrimination ability
Heteroskedasticity	Test result	Significant	Heteroskedasticity detected; cluster-robust SE applied
Overdispersion	Dispersion parameter	1.017	No overdispersion (≈1)
Multicollinearity	VIF (X_centered)	2.46	No multicollinearity (<5)
	VIF (N_centered)	1.21	No multicollinearity (<5)
	VIF (year_2017_2022)	2.55	No multicollinearity (<5)
	VIF (constant)	6.09	Mild multicollinearity (acceptable)
Core result (Logit)	Coefficient on preferential N retention	−2.54	Negative and highly significant

Category	Statistic	Value	Interpretation
	Standard error	0.41	High estimation precision
	z-value	−6.21	Significant at 1% level
	95% CI	[−3.35, −1.74]	Excludes zero
Robustness (Probit)	Coefficient on preferential N retention	−1.54	Same sign as Logit
	p-value	<0.001	Statistically significant
Subsample analysis	Coefficient on preferential N retention	−6.68	Same direction
	p-value	<0.001	Significant
Consistency check	Sign consistency	Yes	Across Logit, Probit, and subsamples
	Significance consistency	Yes (core variable)	Stable inference
Parallel trends	Test conclusion	Not fully satisfied	Results interpreted cautiously
Placebo test	Mean placebo coefficient	−0.01	Centered near zero
	Placebo SD	0.40	—
	True coefficient z-score	−6.32	Strong deviation from placebo
	False significance rate	4.7%	Low
	Placebo conclusion	Robust	Core result unlikely driven by chance

Supplementary Note 4: Logistic regression analysis of Chla and net retention

The variable N_{net} ($N_{DE} > N_{EN}$: $N_{net} = 1$, $N_{DE} < N_{EN}$: $N_{net} = -1$) and the variable P_{net} ($P_{DE} > P_{EN}$: $P_{net} = 1$, $P_{DE} < P_{EN}$: $P_{net} = -1$) have been defined before. Considering the inherent binary classification characteristics and the potential non-linear relationship between N_{net}, P_{net}, and Chla content in lakes, we further use a logistic regression model to analyze their interdependencies across three sampling years.

Our findings suggest a significant negative correlation between Chla content and net N and P retention in lakes during the sampling years of 2012, 2017, and 2022 (Supplementary Fig. S11). This suggests that as eutrophication of the lakes intensifies, as indicated by rising Chla concentrations, the likelihood of lakes that have net retention of N or P gradually decreases. The degree of negative correlation between Chla content and net N retention significantly differs from that of net P retention.

In the logistic regression analysis, key performance indicators for the model include: accuracy (the proportion of correctly predicted samples relative to the total sample size), precision (the proportion of actual positive samples among those predicted as positive by the model), and recall rate (the proportion of actually positive samples correctly identified as positive by the model). Over the three sampling years, the model achieves an accuracy of no less than 0.67, a precision of no less than 0.77, and a recall rate of no less than 0.93. This indicates a relatively high level of precision and strong overall performance.

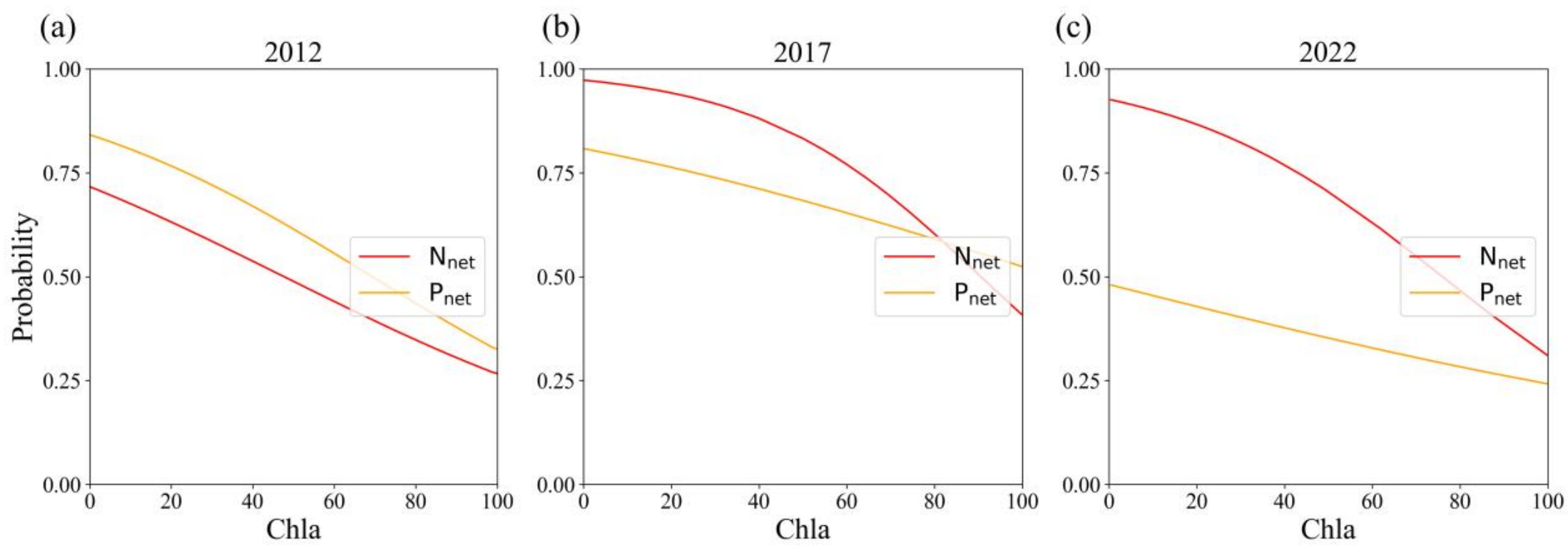

Figure S11 Logistic regression of Chla, net retention of N and P in the three sampling years. "Probability" represents the class probability (the predicted probability that N_{net} or P_{net} equals to 1 under different Chla values). The curves in the three sampling years uniformly show a descending trend, signifying that as the Chla values increase, the predicted probability that N_{net} or P_{net} equals to 1 (positive net retention) will decrease. Source data are provided as a Source Data file.

Supplementary Note 5: External loading

-Calculation details

The computation of Nload and Pload is based on equations (9) - (10) detailed in the Methods section. By calculating the posterior mean of eight parameter distributions (θ_1^P, θ_2^P, θ_3^P, θ_4^P and θ_1^N, θ_2^N, θ_3^N, θ_4^N) across the 10 ecoregions, and integrating these with the measured LU_{forest}, $LU_{wetland}$, f_{NANI}, and f_{NAPI} values, the Nload and Pload for each lake are calculated using equations (5) - (6). The inflow of N and P are calculated via equations (7) - (8).

$$\mathrm{Nload} = \exp(\theta^{N}_{1\,\mathrm{post-mean}} \times f_{\mathrm{NANI}} + \theta^{N}_{2\,\mathrm{post-mean}} \times LU_{\mathrm{forest}} + \theta^{N}_{3\,\mathrm{post-mean}} \times LU_{\mathrm{wetland}} + \theta^{N}_{4\,\mathrm{post-mean}}) \quad (5)$$

$$\mathrm{Pload} = \exp(\theta^{P}_{1\,\mathrm{post-mean}} \times f_{\mathrm{NAPI}} + \theta^{P}_{2\,\mathrm{post-mean}} \times LU_{\mathrm{forest}} + \theta^{P}_{3\,\mathrm{post-mean}} \times LU_{\mathrm{wetland}} + \theta^{P}_{4\,\mathrm{post-mean}}) \quad (6)$$

$$\mathrm{Inflow\ N} = \mathrm{Nload/V} \quad (7)$$

$$\mathrm{Inflow\ P} = \mathrm{Pload/V} \quad (8)$$

-Results for overall and ecoregion patterns

It was observed that the distribution of external of N and P loadings gradually became more concentrated (Supplementary Fig. S12). In 2012, the external N loading data were relatively dispersed, and the median was notably higher than that in other years (2017 and 2022). The data from 2017 displayed a clear trend of concentration, with a lower median than that in 2012. The 2022 data distribution was also relatively focused, and the median further decreased.

The external P loading exhibited different characteristics year-on-year. In 2012, the overall values were low and the distribution was concentrated. In 2017, the data distribution range widened, and the median slightly increased. In 2022, the data showed the highest degree of scatter, and the median was significantly higher than that in 2012 and 2017. A similar conclusion can be drawn from the perspective of N (P) inflows (bearing in mind that inflow refers to external loading after removing the scale effects). Collectively, these findings suggest that in 2012, certain lakes still experienced high external loadings. Since 2017, the external loadings for these lakes have significantly decreased. This is most likely due to the attention drawn by the outliers from these lakes to the USEPA authorities, who have since implemented targeted measures to effectively mitigate the situation.

The overall external loading of N and P shows a declining trend. An analysis on the external N and P loading data from 2012 to 2022 reveals their distinct changing trends. Specifically, the external loading of N significantly decreased from 2012 to 2017, plummeting from 151,143 $g \cdot d^{-1}$ in 2012 to 43,409 $g \cdot d^{-1}$ in 2017. However, it subsequently increased from 2017 to 2022, rising to 47,299 $g \cdot d^{-1}$. Nevertheless, the external loading level of N in 2022 remains significantly lower

than that in 2012. Conversely, the external P loading exhibited a continuous decline, dropping from 18,558 g·d^{-1} in 2012 to 9,109 g·d^{-1} in 2017, and further decreasing to 6,695 g·d^{-1} in 2022. In comparison, the N and P inflows, when adjusted for scale effects, more accurately reflect this downward trend.

The trends in the changes of mean and the median values exhibit a subtle divergence. Specifically, for P, the mean value trends show a consistent decline, while for N, the trend experiences an initial sharp drop followed by a slight upward trend. Overall, both N and P external loadings are on a downward path. The fluctuations in inflow more vividly highlights this declining trend.

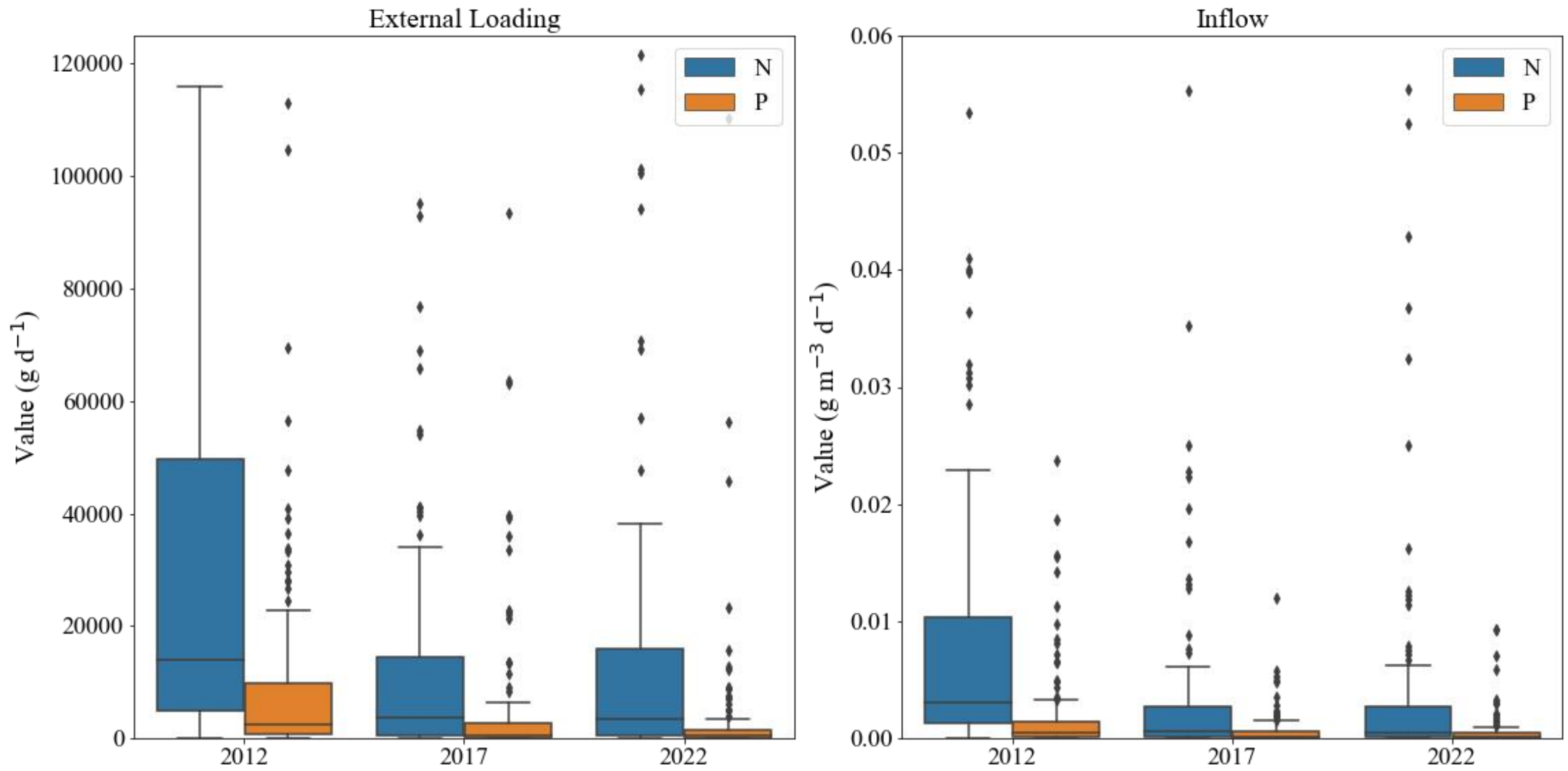

Figure S12 Quantitative statistics of N and P external loading and inflow for 121 lakes under our study across three sampling years in each ecoregion. Source data are provided as a Source Data file.

The performance of lakes in each ecoregion in the external loading and inflow of N and P is consistent with the overall pattern (Supplementary Figs. S13 & S14).

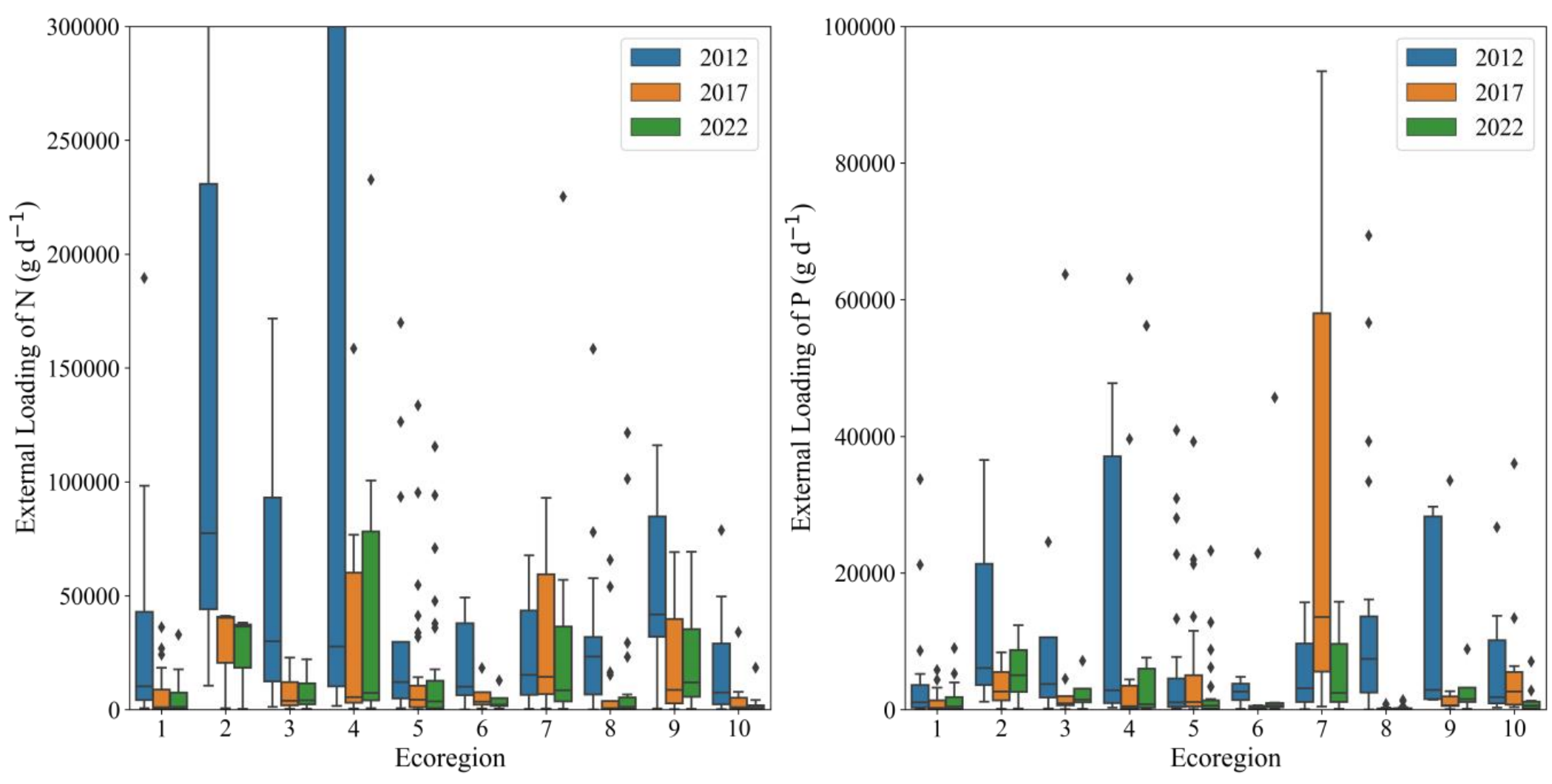

Figure S13 Quantitative statistics of external loading of N and P for 121 lakes under our study in 10 ecoregions across three sampling years. Source data are provided as a Source Data file.

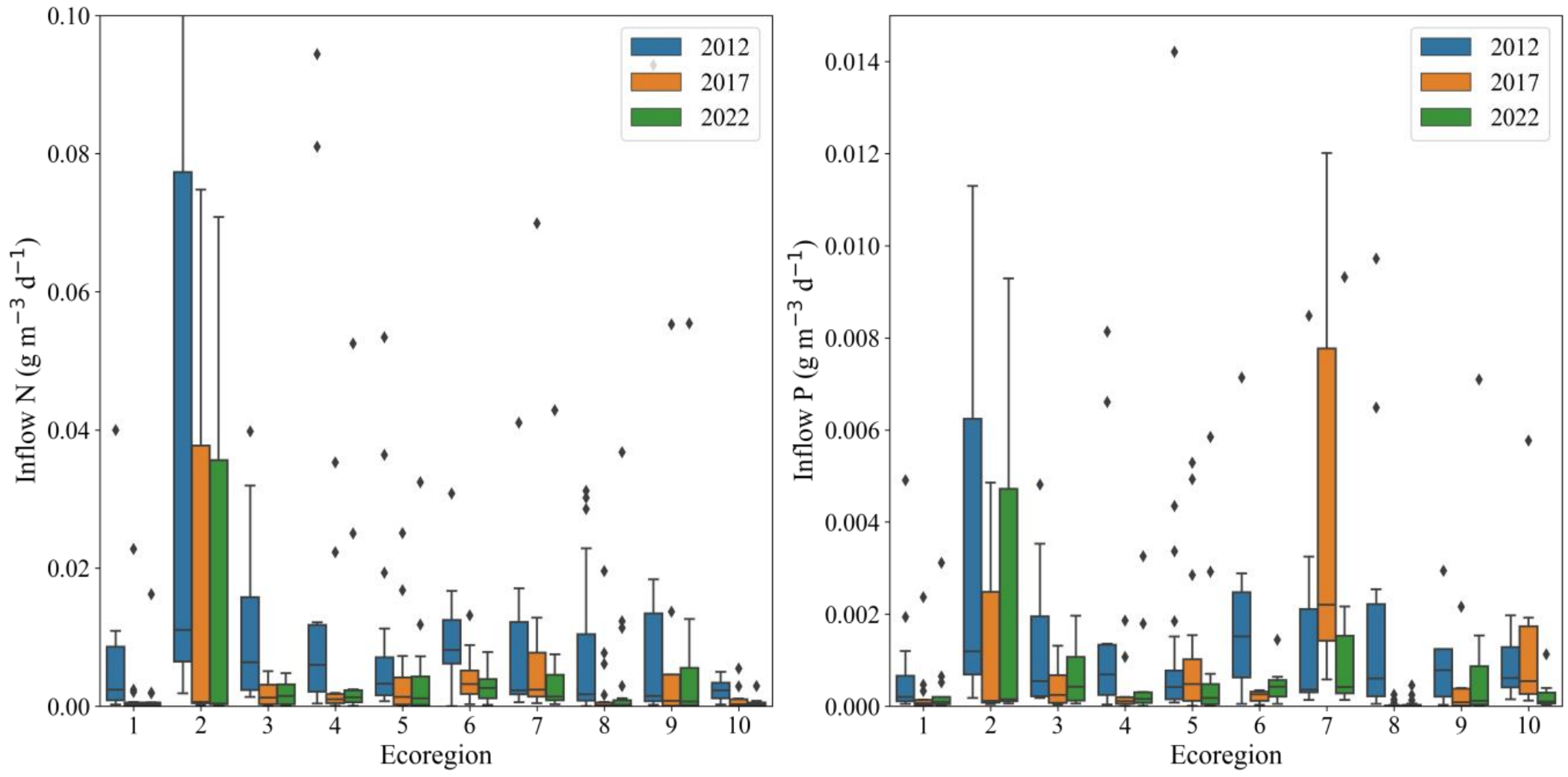

Figure S14 Quantitative statistics of inflow of N and P for 121 lakes under our study in 10 ecoregions across three sampling years. Source data are provided as a Source Data file.

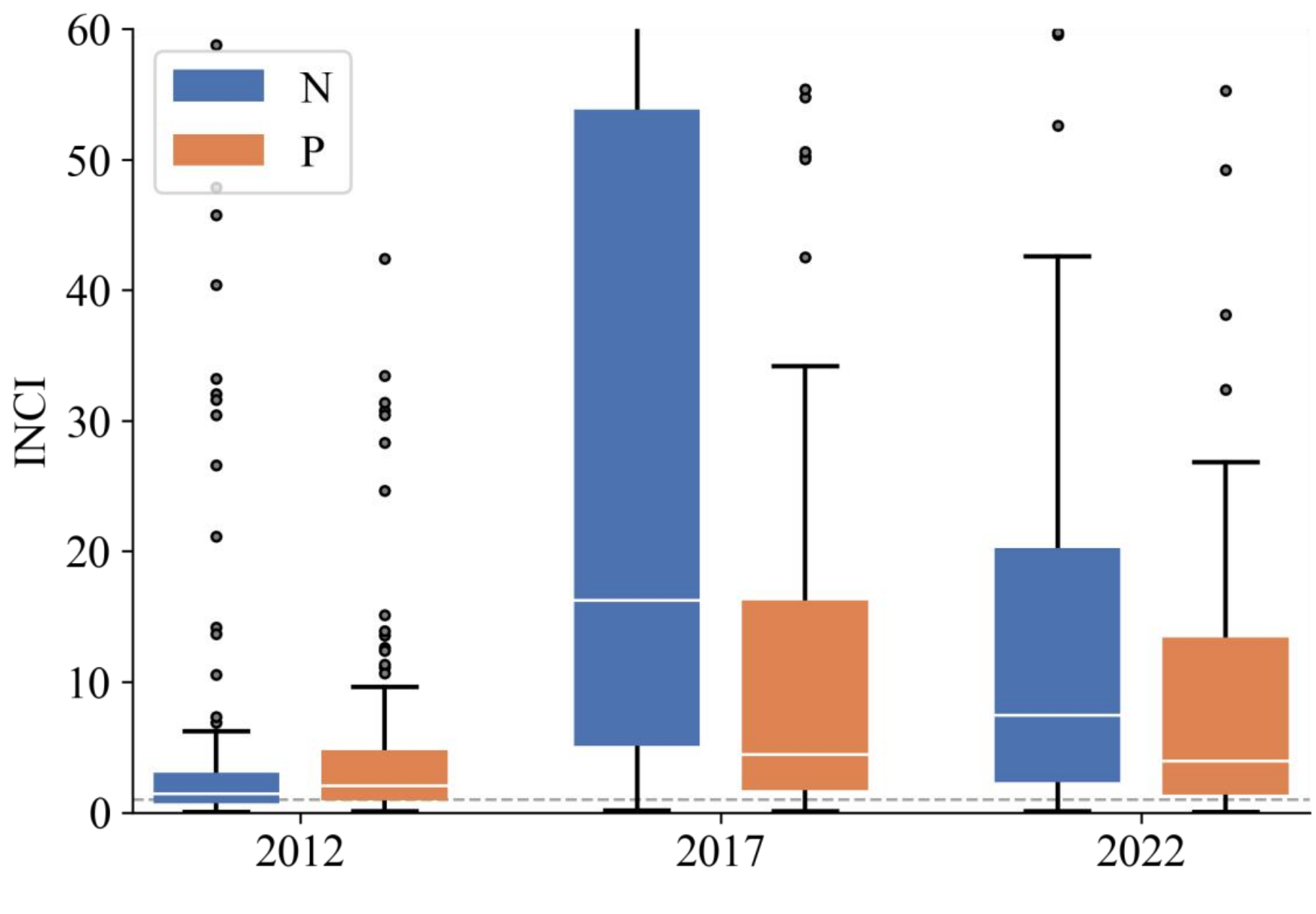

Figure S15 Distribution of N_{INCI} and P_{INCI} values across 121 U.S. lakes. Box-and-whisker plots showing the distributions of nitrogen (N, blue) and phosphorus (P, orange) – related INCI values across 121 U.S. lakes. The boxes indicate the interquartile range (25th – 75th percentiles), the horizontal lines within the boxes represent median values, whiskers extend to 1.5 times the interquartile range, and individual points denote outliers. The grey dashed horizontal line at y = 1

represents the INCI classification threshold (internal dominant model: INCI > 1; external dominant model : INCI <1). Source data are provided as a Source Data file.

Supplementary Note 6: Evaluating alternative explanations for indicator reversals aside from flickering-like dynamcis

Diagnosing flickering in a strict dynamical-systems sense requires long, continuous, and high-frequency time series, which are not available for most U.S. lakes. Accordingly, the purpose of this section is not to formally confirm flickering, but to evaluate whether alternative explanations—particularly externally driven interannual variability—are sufficient to account for the repeated reversals observed in a small subset of lakes. Based on the multiple lines of evidence summarized below, we argue that simple interannual variability alone is unlikely to fully explain the observed patterns.

-Interannual variability:

Interannual variability in nutrient inputs and concentrations has been proposed as a potential explanation for apparent reversals in nutrient retention behavior, especially in the context of long-term phosphorus control efforts in the United States. While such variability undoubtedly exists, several considerations suggest that it is insufficient, on its own, to explain the repeated switching observed in our categorical retention indicators.

First, long-term national and regional assessments indicate that mean nutrient concentrations and external nutrient loads in U.S. lakes have remained broadly stable over the past two decades, with no evidence of coherent interannual oscillations or systematic directional shifts at the continental scale. In the absence of large or coordinated external perturbations, it therefore appears unlikely that interannual variability alone would repeatedly drive binary retention indicators across sign thresholds.

Second, this study deliberately employs categorical (binary) indicators of nutrient rather than raw numerical estimates. While this approach intentionally sacrifices some quantitative resolution, it substantially reduces uncertainty by limiting sensitivity to measurement noise and parameter variability. Our uncertainty and sensitivity analyses demonstrate that moderate perturbations in input variables do not readily induce shifts in indicator classification, making reversals even less likely. Because a reversal requires two consecutive shifts, the probability of such back-and-forth switching is further reduced. Consequently, typical year-to-year fluctuations, even if present, are unlikely to propagate into repeated reversals of categorical retention states.

Taken together, these two lines of evidence indicate that pronounced interannual variability is not apparent in the available data, and that moderate fluctuations, where they occur, are unlikely to

translate into repeated changes in binary retention indicators. On this basis, interannual variability alone appears insufficient to explain the observed patterns.

Third, we examined the occurrence of extreme climatic or hydrological events during the three sampling years and found no indication that the lakes exhibiting repeated reversals experienced major disturbances coincident with the observation periods. This further reduces the plausibility that short-term spikes or episodic external forcing underlie the observed switching behavior.

Fourth, this fails to interpret the emergence of a nutrient metabolic disorder-like state. Logically, if interannual variability of N and P data were solely responsible, one would expect concurrent occurrences of nutrient retention reversal and shift in N/P mass ratios in nutrient inflows and outflows, which is inconsistent with the phenomenon derived from the datasets. The nutrient metabolic disorder-like state suggests much more complex interactions of the factors beyond mere annual fluctuations.

Finally, we acknowledge that interannual variability and noise-induced transitions can produce superficially similar observational patterns, particularly when systems operate near critical thresholds. Distinguishing between these mechanisms would require long, high-frequency time series, which are not available in the present study. Nevertheless, when considered alongside multiple lines of evidence for enhanced internal nutrient cycling intensity (INCI), and our finding that net retention of both nitrogen and phosphorus in U.S. lakes is primarily governed by internal cycling rather than external loading, the observed behavior appears more consistent with internally mediated, noise-amplified dynamics than with externally forced interannual variability.

-Theory of nutrient diffusion： The diffusion of N and P-containing pollutants, particularly the transport of global atmospheric N deposition and riverine N and P to the ocean [1], has been proposed as the primary driver of this recent surge in eutrophication. Analogous to the interannual variability theory, this perspective overly emphasizes external factors as the dominant drivers of shifts, potentially obscuring the complexities of internal processes. It does not align with the output results of N_{load} and inflow N in our model (Supplementary Fig. S12).

-Data distortion: The concern regarding data distortion in N and P measurements, attributed to sampling methodologies, preprocessing procedures, or selective data collection, is a common but unlikely issue. The USEPA employs consistent sampling methodologies and definitions to ensure data integrity [2]. Notably, most aspects of the survey, including indicators, benchmarks, and the methodology for calculating survey results, remained unchanged in 2017 and 2022. The sole potential influence stems from the revised Chla benchmark, where the USEPA reverted to using ecoregional Chla benchmarks for 2017, departing from the World Health Organization's standards adopted in 2012. This adjustment aimed to enhance the survey's accuracy and effectiveness.

Moreover, the selection of lakes adheres to stratified random sampling principles, mitigating concerns regarding selective data collection. The USEPA's reputation and expertise in data collection are well-established and commendable. To further bolster data quality, our preprocessing steps include the identification of overlapping lakes across the ten-year datasets, thereby refining the analytical foundation.

Supplementary Note 7: The reasons that ascribe the reversals to flickering-like dynamics

In the present work, the nutrient retention shift can be considered as flickering-like dynamics. The future observations may continue to reveal shifts in preferential nutrient retention and gradually support the concept of flickering. In comparison with the aforementioned three explanations, flickering not only aligns seamlessly with our observations but also finds ample support from various sources. We thus chose flickering-like dynamics over other explanations for specific reasons:

-Internal impact: Flickering is a warning sign of noise-induced transitions, characterized by changes occurring within the aquatic ecosystems rather than external forces [3]. We have shown that internal nutrient cycling has a dominant impact on retention, as indicated by the INCI indicator, aligning with the characteristics of flickering.

-Observation: Following flickering, noise-induced transitions may occur. The abrupt changes from noise-induced transitions are typically internal qualitative changes. We observed the nutrient metabolic disorder-like state in lakes, which can be considered as an abrupt change from a noise-induced transition, because there is no significant change in external loading in the lakes. This phenomenon has not been observed before. Among the two sources of abrupt changes (noise-induced transitions and bifurcations), noise-induced transitions are fundamentally unpredictable without long-term data, which is challenging to obtain for lakes in the USA. The USEPA collects data every five years, so it is not surprising that nutrient metabolic disorder-like state has not been reported with such a sparse dataset. Other countries are less willing to spend a huge amount of money on continuously observing numerous lakes.

-Qualitative change: Flickering involves qualitative changes, such as high-frequency wet-dry events that happened in the Chew Bahir basin [4]. The current researches on eutrophication could hardly define two states with qualitative differences, so variance and skewness are commonly used to indirectly detect flickering [5]. Several indicators adopted by our research can all intuitively define two different states which can be viewed as qualitative changes, such as preferential nutrient retention (N or P) and net nutrient retention (positive or negative), allowing us to directly detect flickering.

-Model characteristics: In-lake nutrient budget model has been extensively used to mechanistically study the potential for abrupt transitions and early warning indicators to eutrophication due to nutrient dynamics in a lake. We adopted a Bayesian hierarchical framework to run the model in 2000 steps. In general research, this is equivalent to subdividing the time scale

into 2000 parts, with each step representing a time node. In theory, each time node has a pattern of preferential nutrient retention, therefore, the model inherently possesses flickering characteristics, as trophic cascade models in general tend to exhibit such traits. The only problem is that the pattern at each time node is not generated by the measured data, so this can only serve as auxiliary evidence.

Supplementary Note 8: Why we choose the term "nutrient metabolic disorder"

The term "nutrient metabolic disorder" was chosen to describe the preferential retention/interception and release of a certain nutrient in American lakes, mainly because this phenomenon is extremely similar to the nutrient metabolic disorder in human body. Firstly, in the medical field, metabolic disorder is a state of the body, in which there is a pathological and uncoordinated imbalance between the supply and demand of substances during digestion, absorption, and excretion. In this study, the preferential retention/interception and release of a certain nutrient in American lakes also signify an imbalance in the supply and demand of nutrients within the lake ecosystem. Secondly, typical diseases associated with metabolic disorders, such as type 2 diabetes, are characterized by polydipsia, polyphagia, and polyuria. These conditions may present with no symptoms or mild symptoms in the early stages of the disease. In this study, the preferential retention/interception and release of a certain nutrient in U.S. lakes, and the absence of similar reports before 2012, exhibit characteristics very similar to those of type 2 diabetes. Thirdly, the root cause of type 2 diabetes is not an excess sugar intake, but rather a deep-seated metabolic problem. As shown in this study, the net retention in U.S. lakes is predominantly driven by internal cycling rather than external loading, which is very similar to type 2 diabetes. Furthermore, when we compare the human body and the lake system, the rationality of choosing this term is evident throughout the entire process (Supplementary Table S2).

Table S2 Comparison of the human body and the lake system.

Comparison items	Human body	Lake system
Key Concepts	The liver is the central organ for regulating energy and nutrient metabolism in the body, responsible for metabolism of substances such as carbohydrates, fats, and proteins, and is also involved in important physiological functions like detoxification, synthesis, and storage.	The nutrient retention function (process) of the lake system. Through physical sedimentation, biological absorption, chemical fixation, etc., it retains nutrients such as N and P flowing into the lake to maintain the nutrient balance of the lake ecosystem.
	Fatty liver	The huge internal nutrient loading and eutrophication have caused the net retention capacity of the lake to start weakening (reach a saturated state).

Comparison items	Human body	Lake system
	Metabolism	Internal cycling processes
	Fatty liver occurs, leading to a decline in the liver's detoxification ability and triggering systemic metabolic disorders, such as abnormal blood sugar and lipid metabolism.	The retention capacity of the lake reaches a saturated state, making it difficult to achieve self-purification, and then trigger metabolic disorders in the lake.
Abnormal Function Manifestations	Alcoholic fatty liver, mainly caused by long-term and excess alcohol consumption, leads to abnormal fat metabolism in liver and accumulation of fat within liver cells.	The dominance of external loading causes the retention capacity of the lake to reach saturation. For example, agricultural non-point source pollution and industrial wastewater discharge around the lake lead to a large amount of nutrient input exceeding the lake's retention capacity.
	Non-alcoholic fatty liver disease (NAFLD) has become the main cause of chronic liver diseases globally, which is closely related to obesity, insulin resistance, metabolic syndrome, etc.	The saturation of the retention capacity of lakes in the United States that is not dominated by external loading, such as abnormal internal cycling in lakes and other factors, is fully likely to be the main cause of chronic eutrophication, leading to imbalance of the lake ecosystem.
Pathogenic Factors	Metabolic-related diseases are the main pathogenic factors of NAFLD. Diseases such as type 2 diabetes and hyperlipidemia can affect normal metabolic function of the liver.	Problems in the internal cycling are the main causes of abnormal retention function, such as the abnormal release of nutrients in sediments and the imbalance of nutrient uptake and release by organisms.
Specific Disease Manifestations	Type 2 diabetes, a typical metabolic disorder, is clinically manifested as symptoms such as polydipsia, polyphagia, and polyuria.	Nutrient metabolic disorder (NMD) in lakes is manifested as the lake preferentially uptakes nutrients and then releases them, breaking the normal nutrient balance and affecting the health of the lake ecosystem.

Comparison items	Human body	Lake system
Associated Disease Conditions	NAFLD is often accompanied by diseases such as type 2 diabetes. They affect each other and jointly exacerbate the degree of metabolic disorder.	The saturation of retention capacity is accompanied by NMD, leading to further exacerbation of the lake ecosystem and water quality, as well as biodiversity reduction.
Human Body Manifestations	In the case of NAFLD, although some patients have liver fat accumulation, their obesity BMI is not obvious, which increases the difficulty of early diagnosis of the disease.	When retention capacity is saturated, the lake has shown a trend of eutrophication, but from some monitoring indicators, it only slowly increases and the performance is not very obvious, making it easy to be ignored.

Supplementary Note 9: Analysis of the characteristics of the eutrophication wave in U.S. lakes from 2012 to 2022

The eutrophication manifestations of lakes in the United States from 2012 to 2022 can serve as the forefront of a global new wave of eutrophication since 2000. This study provides a summary from two aspects.: 1. The performance of more than 80,000 lakes covered by the USEPA report; 2. The concentrations of total nitrogen (TN), total phosphorus (TP), chlorophyll a (Chla) and trophic states in the 121 lakes in the United States involved in this study.

-USEPA report:

According to the USEPA report, the characteristics of the current round of eutrophication in lakes in the United States can be summarized as follows: Firstly, there is a slight and continuous increase in nutrient levels within the lakes. Through monitoring chlorophyll a (Chla), total nitrogen (TN) and total phosphorus (TP), the USEPA has classified the water quality into three categories: "Good", "Fair" and "Poor". These three indicators can reflect the degree of eutrophication, as the definition of eutrophication can refer to these three indicators, respectively. For example, when Chla > 7 $mg{\cdot}m^{-3}$ (ppb), TN > 0.2$g{\cdot}m^{-3}$ (ppm), or TP > 0.02 $g{\cdot}m^{-3}$ (ppm), it can be defined as eutrophication. Despite the variation in lake samples monitored by the USEPA between 2012 and 2022, the statistical patterns still show a trend of eutrophication in lakes across the United States. From 2012 to 2022, the proportion of lakes classified as "Poor" has been continuously increasing, but the increasing range is relatively small (Supplementary Fig. S16). Secondly, the root causes remain unclear. Anthropogenic factors, rising temperatures, and the increasing frequency of extreme weather events all could create a more favorable environment for eutrophication. However, the abovementioned factors do not show significant manifestations in the USEPA report.

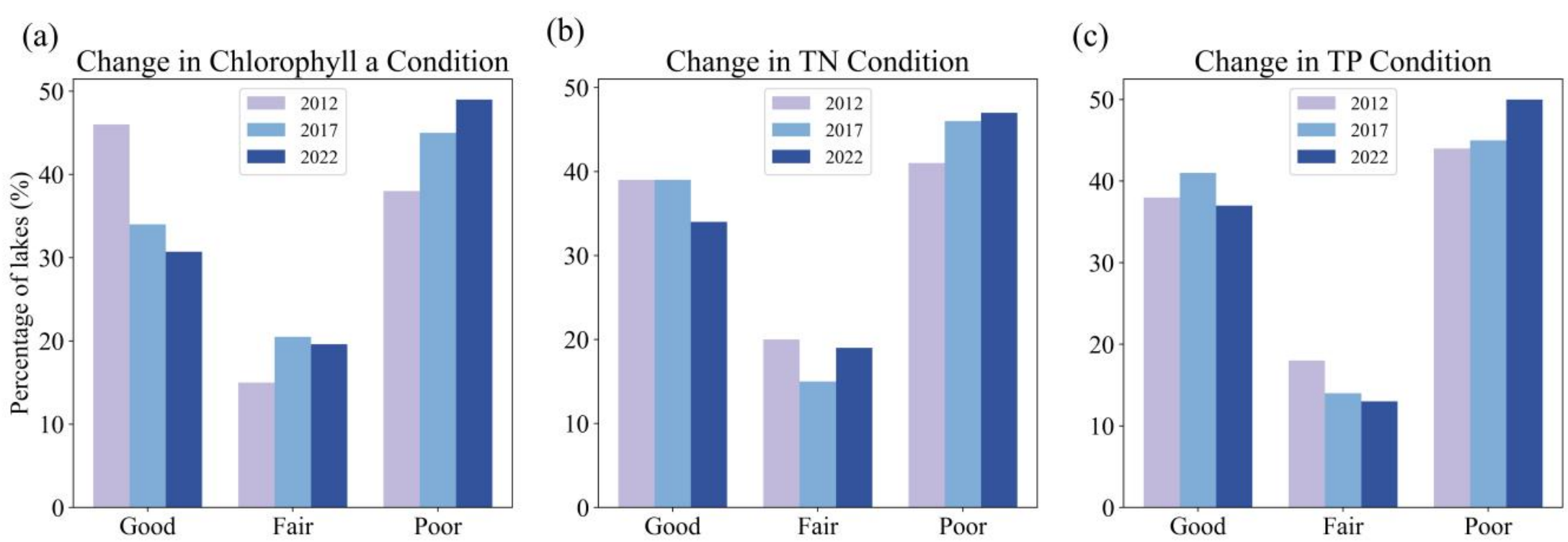

Figure S16 The distribution of Chla, TN and TP in over 80000 lakes in the USA in the three sampling years reported by USEPA. Panels a-c show Chla, TN, and TP, respectively. Source

data are provided as a Source Data file.

-TN, TP, Chla, and trophic states of 121 lakes involved in this study:

According to the tracking analysis of the 121 lakes in this study, the trend of eutrophication is consistently reflected. By classifying Chla, TN, and TP into the same "Good", "Fair" and "Poor" categories, it appears that the 121 lakes have the same distribution as that in the USEPA report (Supplementary Fig. S17). In terms of the trend, the proportion of lakes with "Good" Chla levels decreased, while that with "Poor" Chla levels increased. A similar changing trend occurred for TN. For TP, the proportion of lakes with "Good" levels first increased and then decreased, while that with "Poor" levels gradually increased.

What is different is that the distribution characteristics of the trophic states of these 121 lakes reflect a significant trend towards eutrophication. The proportion of eutrophic lakes has increased from 20.7% in 2012 to 34.7% in 2022 (Supplementary Fig. S18). This indicates that although the increase in Chla is slow, it has made many mesotrophic lakes cross the threshold of 7 mg·m^{-3} and are classified as eutrophic lakes.

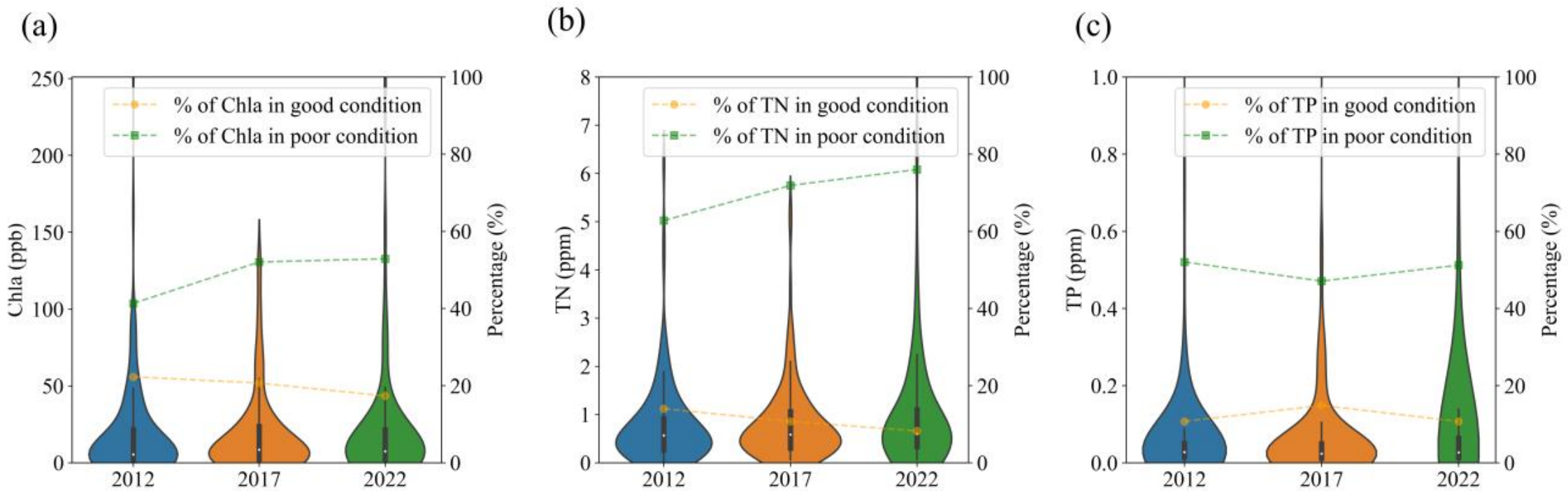

Figure S17 The distribution of Chla, TN and TP in 121 lakes under our study in the three sampling years. Panels a-c show Chla, TN, and TP, respectively. The left axis of the figure employs a violin plot to visualize the statistical distribution of the absolute values, while the right axis overlays a line chart to illustrate proportional trends in the "good" or "poor" ratings of the relevant indicators. Source data are provided as a Source Data file.

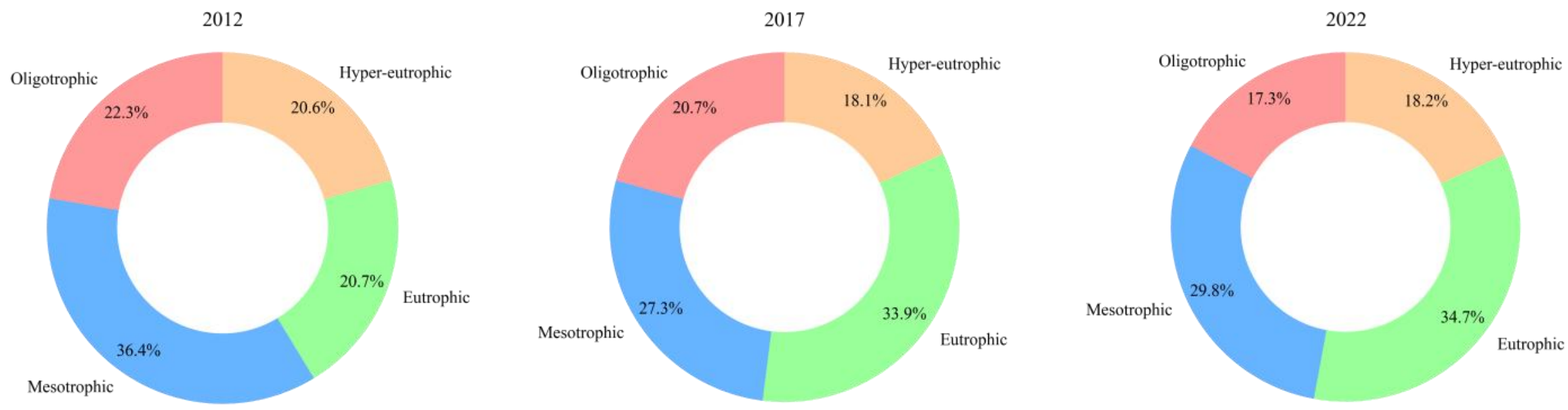

Figure S18 The distribution of the trophic states of U.S. lakes in three sampling years. Source

data are provided as a Source Data file.

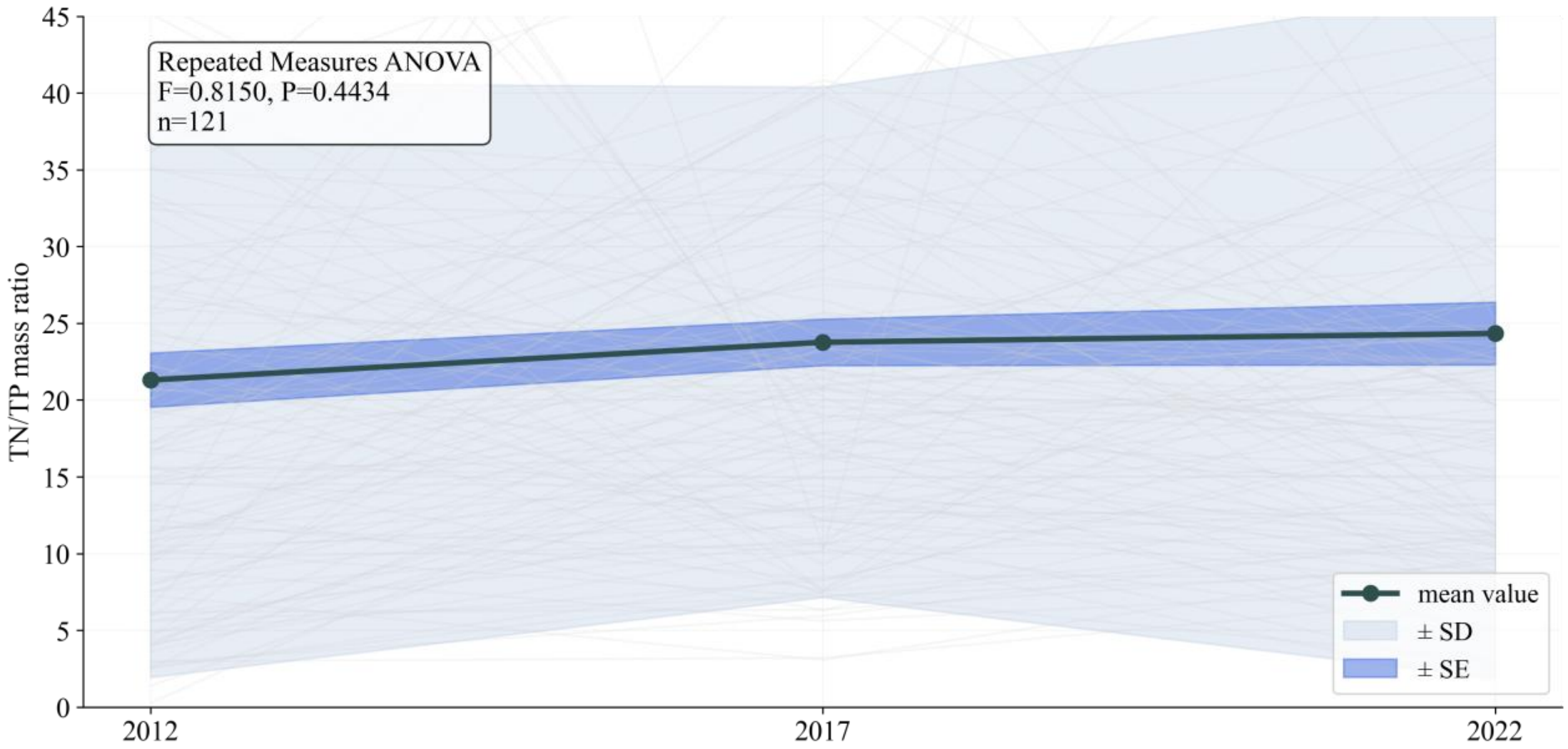

Figure S19 Temporal changes in the TN/TP ratio across 121 U.S. lakes from 2012 to 2022. Thin lines represent individual lakes, while solid circles indicate annual mean values with ±SE (dark shading) and ±SD (light shading). A repeated-measures ANOVA detected no significant temporal change in the TN/TP ratio over the study period (F = 0.8150, P = 0.4434, n = 121). Source data are provided as a Source Data file.

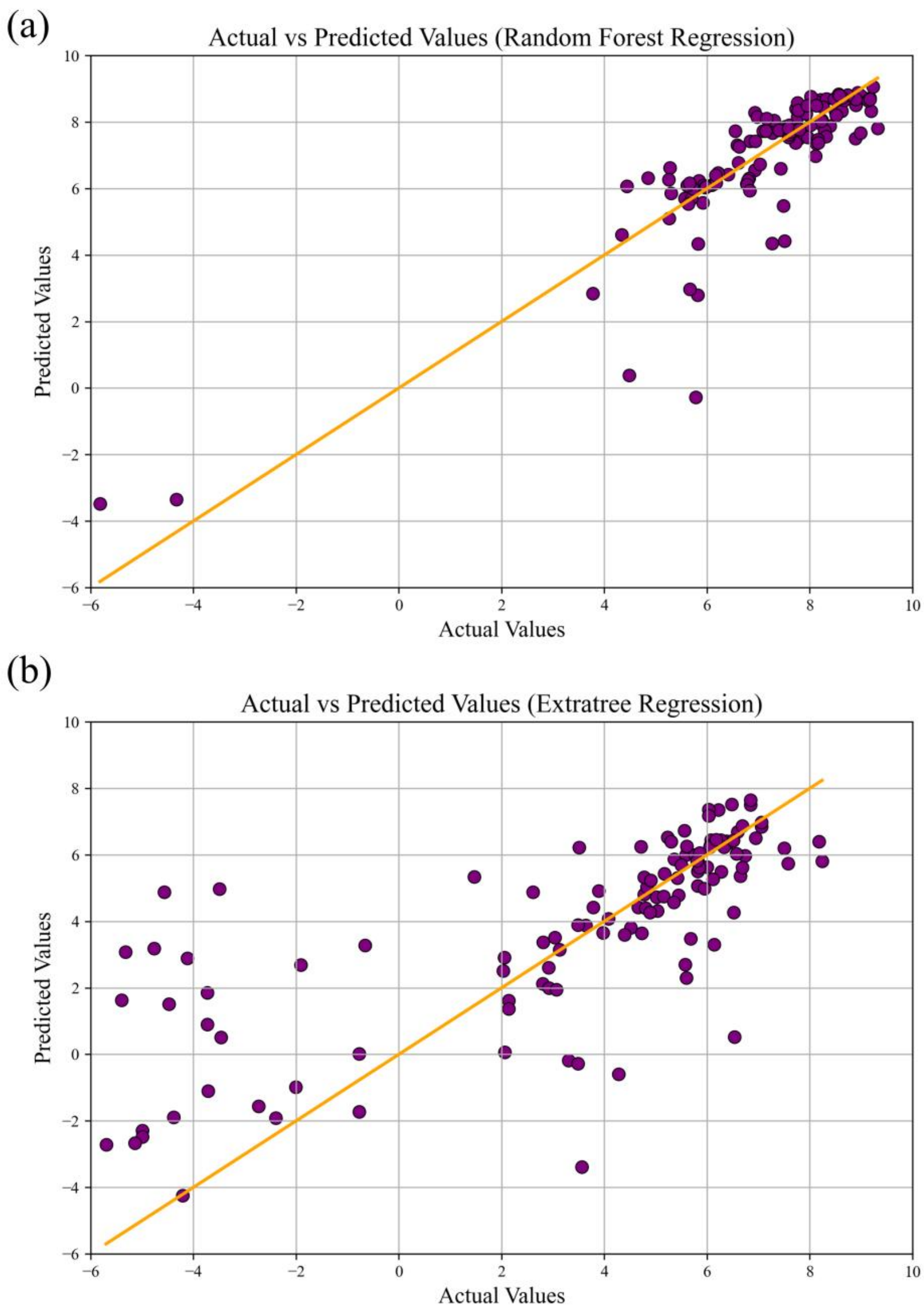

Figure S20 A comparison of the measured- and the predicted-values in the case of using the optimal model fitting for (a) f_{NANI} **and (b)** f_{NAPI}. Source data are provided as a Source Data file.

Table S3 Regression fitting of f_{NANI} based on machine learning.

Regression Method	R^2	MSE	KS Statistic	KS P-Value
Linear Regression	0.06	3.80	0.35	6.43×10^{-7}
Ridge Regression	0.07	3.80	0.35	6.42×10^{-7}
Lasso Regression	-0.01	4.10	0.35	6.42×10^{-7}
SVM	0.42	2.33	0.06	0.99
Decision Tree	0.46	2.21	0.21	0.01
Random Forest	0.71	1.16	0.10	0.59
ExtraTrees	0.62	1.56	0.07	0.95
Deep Learning	0.03	3.95	0.33	3.11×10^{-7}
Gradient Boosting Regression	0.54	1.88	0.22	0.01

Note: The distributional differences between predicted and observed values were evaluated using the two-sample Kolmogorov-Smirnov (KS) test. The test was two-sided.

Table S4 Regression fitting of f_{NAPI} based on machine learning.

Regression Method	R^2	MSE	KS Statistic	KS P-Value
Linear Regression	0.05	13.97	0.48	1.29×10^{-12}
Ridge Regression	0.05	13.96	0.48	1.29×10^{-12}
Lasso Regression	0.06	13.81	0.48	1.29×10^{-12}
SVM	0.25	11.07	0.18	0.04
Decision Tree	0.36	9.34	0.29	6.63
Random Forest	0.46	7.87	0.15	0.13
ExtraTrees	0.53	6.92	0.13	0.24
Deep Learning	0.05	2.10	0.51	8.21×10^{-14}
Gradient Boosting Regression	0.38	9.13	0.20	0.02

Note: The distributional differences between predicted and observed values were evaluated using the two-sample Kolmogorov-Smirnov (KS) test. The test was two-sided.

Supplementary Note 10: Uncertainty and sensitivity

1. The advantages of Bayesian hierarchical method compared to the traditional methods.

In traditional methods, each parameter is estimated in isolation, with each parameter based on statistical models or empirical formulas, lacking a reasonable comprehensive reference standard. This independent estimation approach leads to complexity and uncertainty in the estimation process, especially when there are certain interactions and correlations between parameters. Handling each parameter in isolation often fails to accurately reflect its true value. Moreover, traditional methods often do not fully utilize all information in the original data, especially when dealing with multiple ecological variables or complex ecological systems, where the correlations and inherent patterns in the data are often overlooked. When processing large-scale ecological data, parameters between different ecological regions usually have certain inherent connections and interactions. For example, the N and P enrichment coefficients in certain regions may be influenced by the same environmental factors, or the enrichment processes of N and P in different regions may follow similar patterns. The flaw in traditional methods lies in treating these ecological regions as independent units for estimation, ignoring the potential correlations between regions.

On the contrary, the Bayesian hierarchical method has the following significant advantages: First, it enables parameter integration and hierarchical fitting. The model closely integrates multiple related parameters through formulas and uses the existing real-world data for hierarchical fitting. This approach effectively captures the inherent connections between parameters, reduces the uncertainty caused by independent estimation, and improves the model's precision. Second, it incorporates reliable prior distributions. Bayesian methods allow the introduction of prior knowledge, such as descriptions of parameter distributions in different ecological regions from previous literature. These authoritative prior distributions provide a solid foundation for the model, enhancing reliability of the estimated results. Third, the posterior distribution presents a complete picture of uncertainty. Since the model treats parameters as random variables, it can output their posterior distributions, offering a comprehensive perspective for uncertainty analysis. When applied to temporal scales, the posterior distribution from the previous round can be used as the prior distribution for the new round, simulating and maintaining temporal consistency to further reduce uncertainty. Fourth, it enables full-process uncertainty control. Bayesian methods systematically control and quantify uncertainty throughout the entire modeling process. By calculating metrics such as standard deviation, Monte Carlo standard error, confidence intervals, and effective sample size, the model clearly demonstrates the sources and degrees of uncertainty, providing critical evidence for interpreting results and making decisions.

2. The uncertainties and sensitivity of the sub-parameter estimates

In the framework of the Bayesian hierarchical model, "sub-parameters" generally refer to the parameters at lower tiers within the hierarchical structure. For example, μ_A_N is a sub-parameter. In the

Bayesian hierarchical method, the parameters of lakes are treated as random variables that follow their respective normal distributions, but the mean (μ) and standard deviation (σ) of each parameter (μ and σ are sub-parameters) are also treated as random variables following normal distributions.

We quantified the uncertainties associated with the output sub-parameter estimates using MCSE (Monte Carlo standard error), STD (standard deviation), 5%-95% confidence intervals, and ESS (effective sample size) (Supplementary Tables S5 & S6). The Monte Carlo Standard Error (MCSE) is specifically used to measure the uncertainty of estimates from Monte Carlo simulations. A lower MCSE indicates more precise estimates and more stable sampling outcomes. In absolute terms, apart from A_N, which exhibits relatively higher uncertainty, the uncertainty of all other indicators is extremely low. The standard deviation (STD) reflects uncertainty through the degree of dispersion. In absolute terms, except for the two indicators A_N and A_P, which show greater dispersion, all other STD values are less than 1, indicating very concentrated estimates and minimal uncertainty. The confidence interval (5%-95%) reflects the concentration of data, and thus uncertainty, through the width of the interval. In absolute terms, except for A_N and A_P (which have greater dispersion), the widths of the other confidence intervals range from 1 to 6, indicating very concentrated estimates and minimal uncertainty. The effective sample size (ESS) reflects the amount of independent information in the sample; a larger ESS indicates more reliable information from the sample and lower uncertainty. In disciplines such as Bayesian analysis, an ESS greater than 100 is often considered to provide relatively reliable estimates. From the vantage point of ESS, the uncertainty of all parameters is extremely low. The primary reason for the relatively higher uncertainty in A_N and A_P remains that the means and variances of their prior distributions are both high. When assessed by the ratio of STD to the mean of their prior distribution, the means and standard deviations of these two indicators actually fluctuate within a range of no more than 15% around the mean of their prior distribution, signifying that the actual uncertainty is minimal. The comprehensive evaluation results indicate that the uncertainties of all output sub-parameter estimates remain at a low level. We obtained full posterior distributions for sub-parameters in each group to characterize the sensitivity of output sub-parameter estimates to 1000 sets of input parameters through the Hamiltonian Monte Carlo method (Supplementary Figs. S23 & S24). The posterior distribution is continuous and exhibits a sharply peaked curve, indicating a low sensitivity to variations in the input parameters.

Table S5 The uncertainty indicators of the mean and standard deviation of the random variable for each parameter of Michaelis–Menten function (averaged over three sampling years). The uncertainty indicators include MCSE (Monte Carlo Standard Error), STD (Standard Deviation), 5%-95% Confidence Interval, and ESS (Effective Sample Size). The performance indicators include N_EFF/S (Effective Sample Size per Second) and $\hat{R}$ (Gelman-Rubin Diagnostic). With the exception of A_N, all other parameters exhibit extremely low uncertainty. The elevated uncertainty associated with the parameter A_N stems from its prior distribution featuring both a high mean and variance. However, if the evaluation uses a ratio of MCSE/mean, the values of μ_A_N (0.0056) and σ_A_N (0.0026) reveal that the parameter's actual variation around the prior distribution remains within 1% (below 10% is ideal normally), effectively demonstrating negligible uncertainty.

Parameters	MCSE	STD	5%	95%	ESS	N_EFF/S	$\hat{R}$
μ_A_N	121.68	2220.89	17101.33	26854.90	631.70	12.03	1.01
σ_A_N	5.50	300.11	1531.59	2520.40	3019.83	55.37	1.00
μ_A_P	12.47	354.47	1893.94	3067.95	893.44	15.98	1.01
σ_A_P	2.78	95.78	321.67	635.06	1576.65	25.33	1.00
μ_B_N	0.00	0.26	1.22	2.08	2958.24	53.89	1.00
σ_B_N	0.00	0.10	0.83	1.16	4370.35	79.05	1.00
μ_B_P	0.01	0.28	1.66	2.59	2391.71	45.08	1.00
σ_B_P	0.00	0.10	0.84	1.17	2839.61	45.93	1.00
μ_M_N	0.05	1.98	14.35	20.84	1356.24	24.06	1.00
σ_M_N	0.03	0.98	1.85	5.07	1303.20	22.71	1.00
μ_M_P	0.00	0.10	1.88	2.21	4108.32	71.15	1.00
σ_M_P	0.00	0.10	0.84	1.18	2884.36	51.59	1.00
μ_S_N	0.00	0.00	0.00	0.00	1631.52	27.93	1.00
σ_S_N	0.00	0.01	0.00	0.02	1025.99	19.02	1.01
μ_S_P	0.00	0.00	0.01	0.02	2312.92	42.12	1.00
σ_S_P	0.00	0.00	0.01	0.01	3209.86	56.56	1.00

Table S6 The uncertainty indicators for the mean and standard deviation of the random variable for each parameter of loading function (averaged over three sampling years). All parameters exhibit extremely low uncertainty.

Parameters	MCSE	STD	5%	95%	ESS	N_EFF/S	R^
$\mu\_\theta_1^N$	0.00	0.04	0.29	0.41	1393.49	25.96	1.00
$\sigma\_\theta_1^N$	0.00	0.01	0.04	0.07	2111.04	38.31	1.00
$\mu\_\theta_2^N$	0.00	0.00	-0.06	-0.04	3791.68	66.54	1.00
$\sigma\_\theta_2^N$	0.00	0.03	0.01	0.09	958.96	21.11	1.01
$\mu\_\theta_3^N$	0.00	0.01	-0.03	-0.01	2044.04	37.53	1.00
$\sigma\_\theta_3^N$	0.00	0.01	0.00	0.02	599.32	12.07	1.01
$\mu\_\theta_4^N$	0.01	0.18	2.41	3.01	1380.79	25.02	1.00
$\sigma\_\theta_4^N$	0.00	0.10	0.10	0.43	952.58	18.54	1.00
$\mu\_\theta_1^P$	0.00	0.05	0.10	0.27	1667.90	31.33	1.00
$\sigma\_\theta_1^P$	0.00	0.02	0.07	0.14	1222.70	21.36	1.00
$\mu\_\theta_2^P$	0.00	0.01	-0.02	-0.01	2587.12	48.22	1.00
$\sigma\_\theta_2^P$	0.00	0.01	0.00	0.02	787.70	15.86	1.00
$\mu\_\theta_3^P$	0.00	0.01	-0.03	-0.01	1819.86	34.19	1.00
$\sigma\_\theta_3^P$	0.00	0.01	0.00	0.03	372.05	7.01	1.02
$\mu\_\theta_4^P$	0.01	0.47	2.34	3.88	4112.99	72.49	1.00
$\sigma\_\theta_4^P$	0.00	0.13	3.07	3.49	3952.23	74.51	1.00

3. The uncertainties and sensitivity of the model-derived results

The model-derived results (proportion of lakes exhibiting net retention, preferential retention, and internal dominant model for N and P) are from percentage calculations based on the categorical variable. For example, to reflect the overall picture of net N retention in U.S. lakes, we first assess whether each lake exhibits net N retention ($N_{DE} > N_{EN}$) and then calculate the proportion of lakes with net N retention, rather than calculating the absolute quantity of net N retention (N_{DE} - N_{EN}). The advantage of using percentage calculations for the categorical variables is the substantial reduction in uncertainty. For example, in a single lake with net N retention, if N_{EN} and N_{DE} fluctuate by 20% around their estimated values, the lake may still be categorized as net N retention, resulting in an uncertainty close to 0. If we calculate the quantitative variables using the traditional method, N_{DE} - N_{EN} could also fluctuate by 20% around their estimated values.

The uncertainty of model-derived results can be quantified using the misclassification percentage error. We first estimate the uncertainty of N_{EN}, N_{DE}, P_{EN}, P_{DE}, L_N and L_P , then calculate the probability of misclassifying the categorical variable for each lake (Supplementary Table S7). Through expected value calculations, we finally derive the misclassification percentage error for 121 lakes (Supplementary Table S8). The error margins for all results remain within the following limits: net N retention (± 0.80 pp, percentage point), net P retention (± 0.40 pp), preferential retention of N and P (± 0.53 pp), N internal dominant model (± 6.4×10^{-5} pp), and P internal dominant model (± 0.014 pp). The extremely low uncertainty in the model results fully highlights the significant advantage of using percentage calculations for categorical variables over traditional quantitative variable calculations.

Based on the posterior distributions, the sensitivity of model-derived results to input parameter variability was further evaluated (Supplementary Table S9). It is evident that the model-derived results displayed low sensitivity to variations in input parameters, underscoring the robustness of the model.

Table S7 The lower bound of the probability that the estimate fluctuates within the corresponding range around the estimated value. The table shows that the probabilities of N_{EN}, P_{EN}, N_{DE}, and P_{DE} fluctuating within ranges not exceeding 20% of their estimated values are greater than 95.44%, 84.73%, 68.26%, and 78.87%, respectively, while the probabilities of L_N and L_P fluctuating within ranges not exceeding 50% of their estimated values are at least 68.26% and 54.68%, respectively. The empty spaces in the table, where the values are close to 100%, indicate that N_{EN}, P_{EN}, N_{DE}, and P_{DE} never fluctuate by more than 40% around their estimated values.

Variation range	N_{EN}	P_{EN}	N_{DE}	P_{DE}	L_N	L_P
±20%	95.44	84.73	68.26	78.87	31.08	23.58
±30%	99.73	96.42	86.64	93.70	45.14	34.72
±40%	-	-	-	-	57.62	45.14
±50%	-	-	-	-	68.26	54.68

Table S8 The uncertainty ranges of the model-derived results. The model-derived results (proportion of lakes exhibiting net and preferential retention, as well as internal dominant model for N and P) are from percentage calculations based on categorical variables. Particularly noted that this uncertainty is a roughly estimated error upper bound, as the current error is already small enough. If it is further divided into each sampling year (2012, 2017, and 2022), a smaller upper bound of uncertainty can be obtained.

Proportion of categorical variables	Uncertainty range
Net N retention	±0.80 pp
Net P retention	±0.40 pp
Preferential retention of N and P	±0.53 pp
N internal dominant model	$\pm 6.4\times10^{-5}$ pp
P internal dominant model	±0.014 pp

Table S9 The sensitivity of model-derived results. It is worth noting that all model-derived results are from percentage calculations based on categorical variables. Thus, the parameter sensitivity of these results can be evaluated by examining the percentage changes in categorical variables when input parameters are varied by ± 5%.

Proportion of categorical variables	Variation range
Net N retention	±1.65 pp
Net P retention	±0.83 pp
Preferential retention	±3.31 pp
N internal dominant model	±4.96 pp
P internal dominant model	±6.61 pp

4. Sensitivity of model inference to uncertainty in f_{NAPI}

In this study, f_{NAPI} was estimated using multiple state-of-the-art machine learning algorithms, among which Extra Trees yielded the best predictive performance. Nevertheless, model evaluation indicated a moderate predictive power, with a mean positive prediction bias of 0.47 on the test dataset, where 54.17% of samples were overestimated and 45.83% underestimated.

Rather than assuming f_{NAPI} to be precisely constrained, we explicitly accounted for this uncertainty within a Bayesian hierarchical modeling framework. Compared to traditional deterministic or single-level statistical models, Bayesian hierarchical models are less sensitive to upstream uncertainty in forcing variables, as uncertainty can be partially absorbed at higher hierarchical levels and propagated probabilistically through the model structure. In addition, our inference relies primarily on binary response metrics, further reducing sensitivity to point-wise prediction errors in continuous input variables such as f_{NAPI}.

To directly assess the influence of potential systematic bias in f_{NAPI} on the final inference, we conducted a conservative sensitivity analysis by uniformly reducing f_{NAPI} values in 2017 and 2022 by 0.47, corresponding to the mean positive prediction bias identified during model evaluation. Even under this substantial modification, the key inferred patterns—including preferential nutrient retention, net retention, internal dominant model, remained effectively unchanged (Supplementary Table S10).

Table S10. Sensitivity of key model outcomes to systematic bias in f_{NAPI}

Key results	Main Text	Bias-corrected f_{NAPI} (−0.47)
2017		
Lakes showing preferential P retention (%)	14.88	17.36
Lakes showing preferential N retention (%)	85.12	82.64
N−P− (%)	6.61	6.61
N−P+ (%)	2.48	2.48
N+P− (%)	17.36	18.18
N+P+ (%)	73.55	72.73
2022		
Lakes showing preferential P retention (%)	7.44	5.79
Lakes showing preferential N retention (%)	92.56	94.21
N−P− (%)	14.88	14.88

Key results	Main Text	Bias-corrected f_{NAPI} (−0.47)
N−P+ (%)	1.65	1.65
N+P− (%)	42.15	42.98
N+P+ (%)	41.32	40.50

We additionally reproduced all figures directly supporting the main conclusions using the bias-corrected f_{NAPI} values; the resulting patterns are visually consistent with those presented in the main text, with only marginal differences in detail (Supplementary Fig. S21 & S22).

These results provide a concrete example supporting the methodological advantages discussed in Section 1 and the substantial reduction in uncertainty afforded by percentage-based categorical inference described in Section 3.

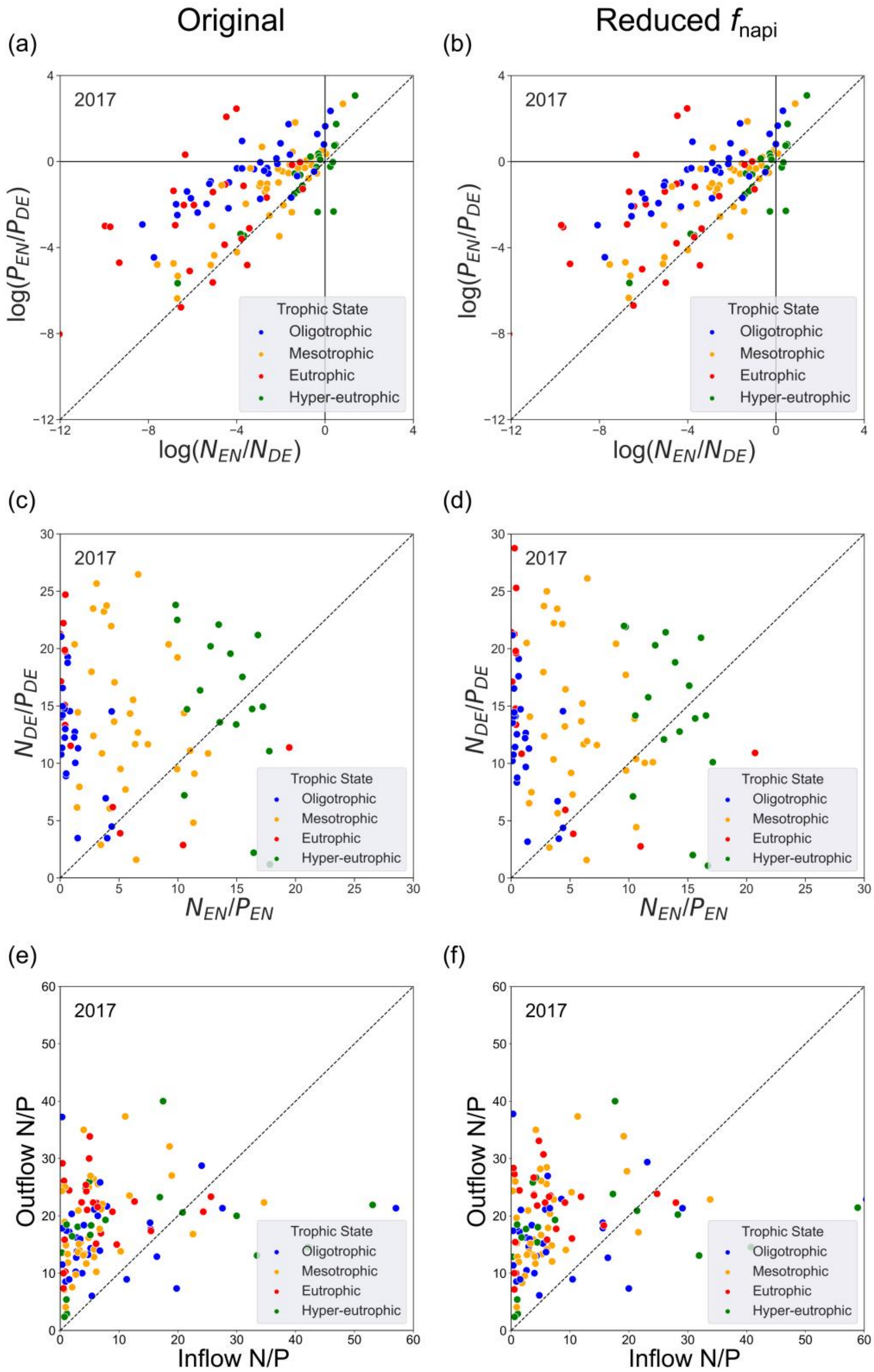

Figure S21 Sensitivity of model outputs to systematic bias correction in f_{NAPI} for 2017. Comparison of model outputs for 2017 using the original f_{NAPI} estimates (left panels, **(a), (c), (e)**) and bias-corrected f_{NAPI} values reduced uniformly by 0.47 (right panels, **(b), (d), (f)**), corresponding to the mean positive prediction bias identified in model evaluation. Source data are provided as a Source Data file.

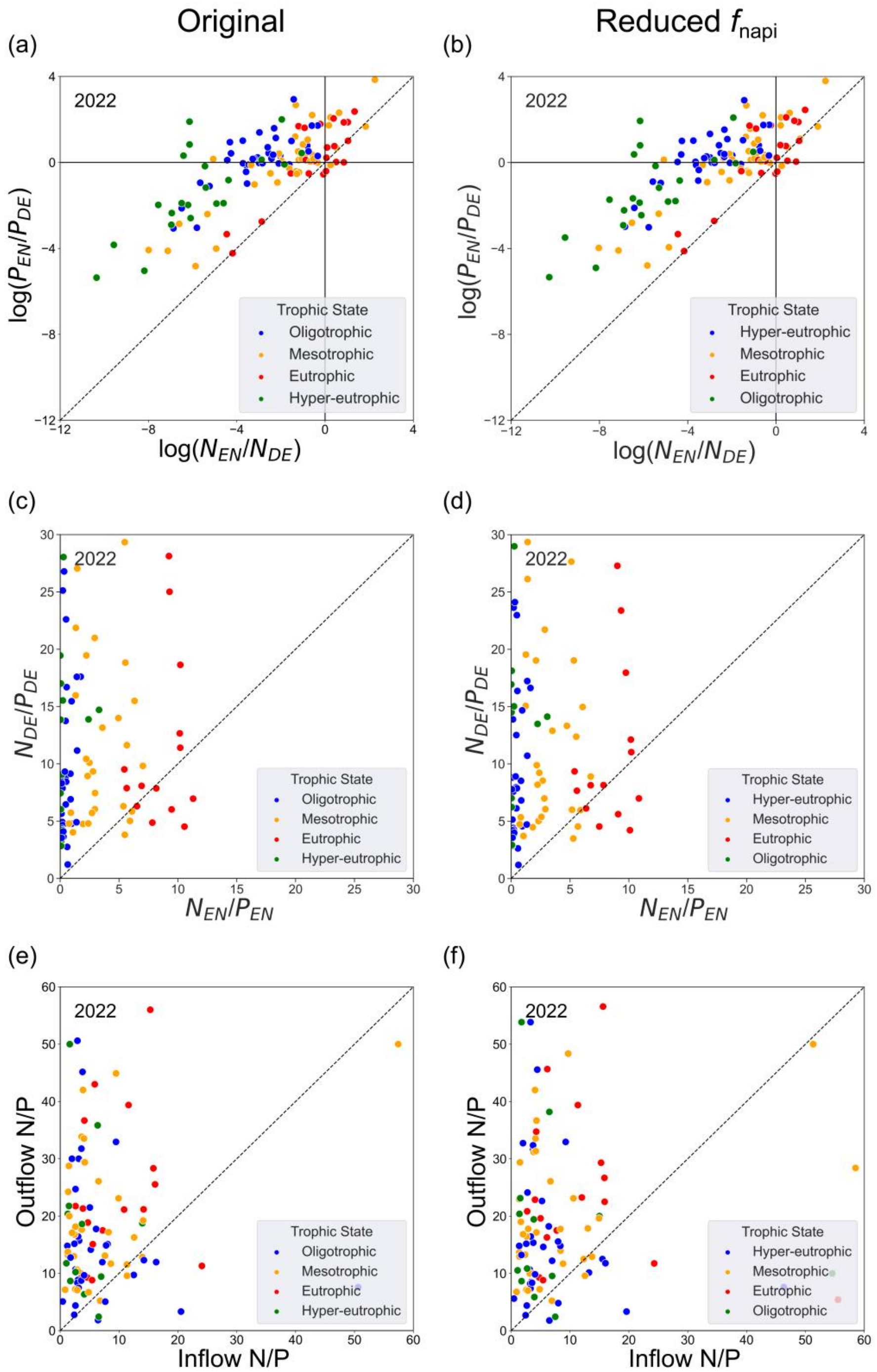

Figure S22 Sensitivity of model outputs to systematic bias correction in f_{NAPI} for 2022. Comparison of model outputs for 2022 using the original f_{NAPI} estimates (left panels, **(a), (c), (e)**) and bias-corrected f_{NAPI} values reduced uniformly by 0.47 (right panels, **(b), (d), (f)**), corresponding to the mean positive prediction bias identified in model evaluation. Source data are provided as a Source Data file.

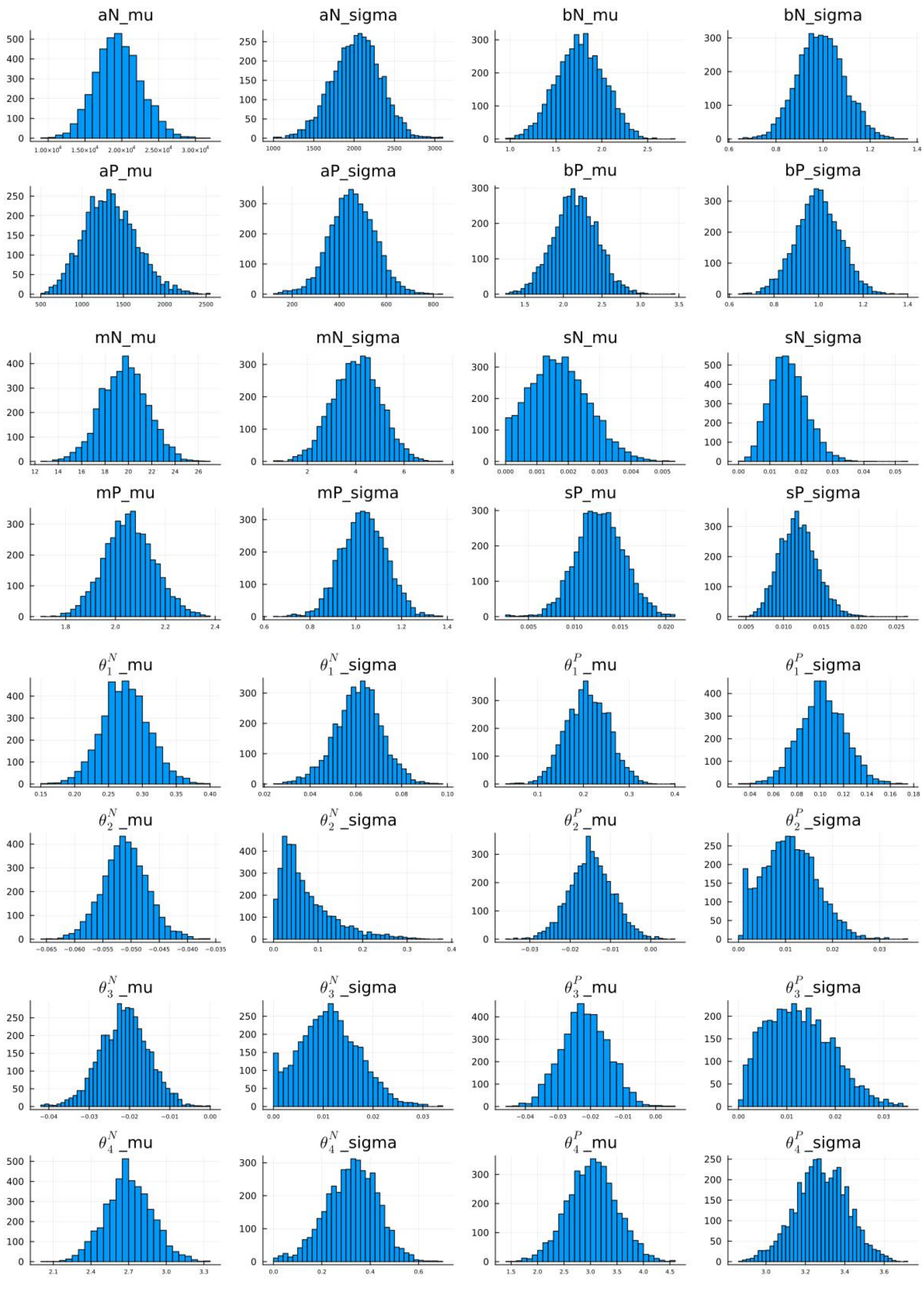

Figure S23 Posterior distributions of the parameters for the nutrient budget model of 2017.

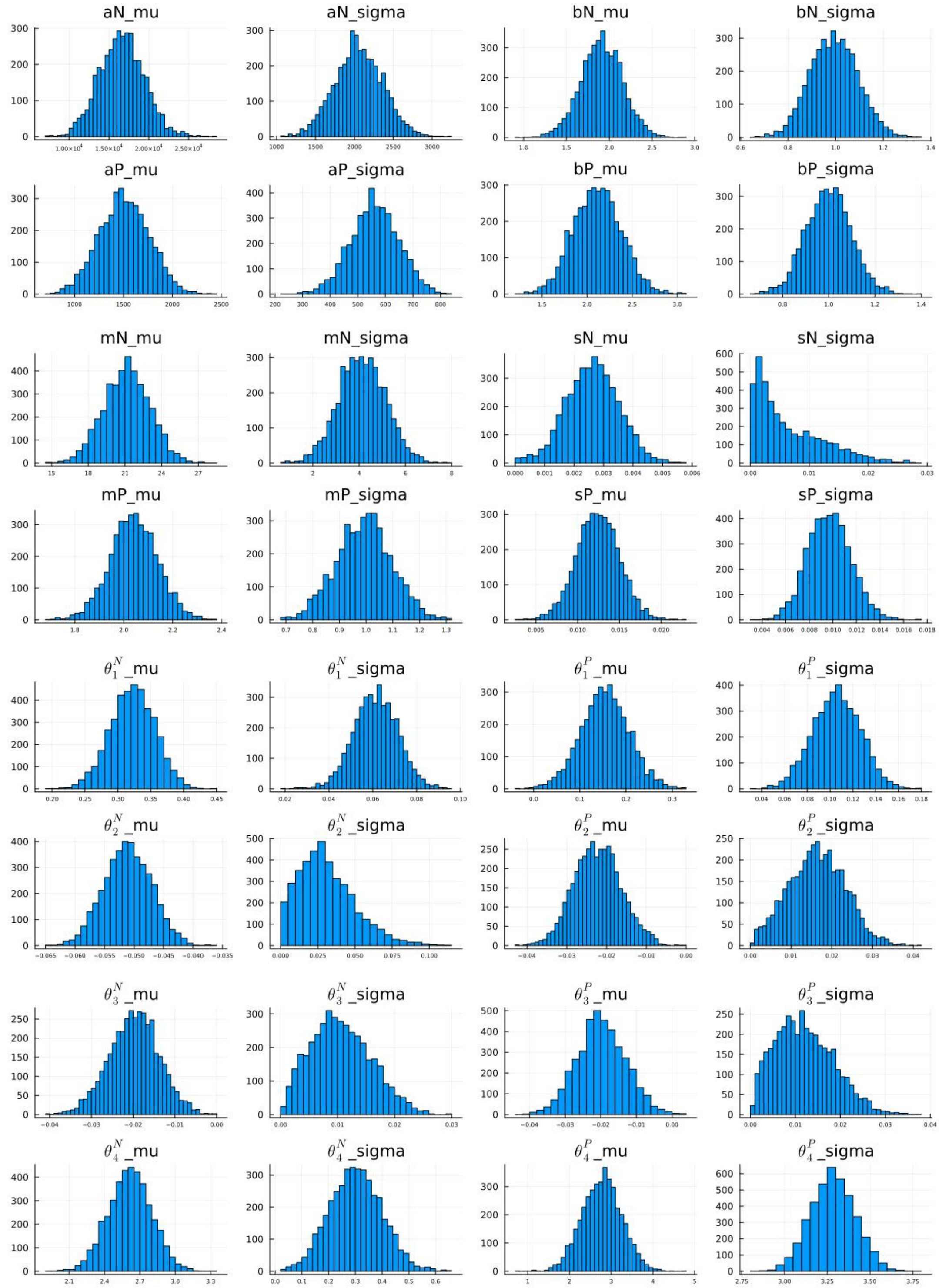

Figure S24 Posterior distributions of the parameters for the nutrient budget model of 2022.

Supplementary Note 11: Field sampling details of USL datasets

1. Target

The lakes selected based on USL data in this study are mainly from the EPA NLA, due to EPA's stringent lake sample selection process and the NLA's representative nature of lakes in the United States. The target for the NLA includes all lakes, reservoirs, and ponds within the 48 contiguous United States greater than 1 hectare (ha) in surface area that are permanent waterbodies, at least 1 m deep, and have a minimum 0.1 ha of open water. Additionally, lakes are required to have a minimum residence time of one week. The Great Lakes, Great Salt Lakes, and lakes that are tidally influenced are excluded; as are those used for aquaculture, disposal-tailings, sewage treatment, evaporation, or other unspecified disposal use.

2. Time

Field data were collected between May and October in three survey years of 2012, 2017 and 2022.

3. Survey design implementation

Usually, field crews spent a whole day collecting chemical, physical, and biological data at each lake. In general, field crews evaluated lakes from the NLA survey design using a variety of techniques including aerial photo interpretation, GIS analyses, local knowledge, etc. to identify locations that did not meet the NLA lake definition. Crews also excluded lakes from sampling during field reconnaissance if they were non-target types or could not be assessed due to accessibility issues (e.g., landowner permission, too dangerous to access, etc.).

4. Indicators

4.1 Nutrients and Chla

The USEPA only evaluated total P (TP) and total N (TN) as the nutrient assessment criteria. Near the surface (at depths up to 2 meters), field crews collected samples for nutrients and Chla using a long plastic tube, as described in the NLA report.

4.2 Physical indicators

Physical indicators including discharge rate, WRT, lake volume, watershed area, LU_{forest} and LU_{wetland} are all from the dataset of Wu et al.[3]. LU_{forest} and LU_{wetland} are derived from NLA Landscape datasets. Here, LU_{wetland} is the sum of LU_{woody} (percentages of watershed area classified as woody wetland land cover) and $LU_{\text{herbaceous}}$ (percentages of watershed area classified as herbaceous wetland land cover); LU_{forest} is the sum of $LU_{\text{deciduous}}$ (percentages of watershed area classified as deciduous forest land cover) and $LU_{\text{evergreen}}$ (percentages of watershed area classified as evergreen forest land cover). For other indicators, Wu et al.[3] obtained the data by referencing a dataset organized by

Messager et al., [6] termed HydroLAKES. Therefore, the method for measuring these parameters comes from Messager et al.[6] HydroLAKES distinguishes 1.43 million individual polygons of natural lakes and human-made reservoirs with a surface area of at least 10 ha.

To acquire the individual WRT and discharge rate for each lake, Messager et al.[6] used estimates of long-term (1971–2000) average ‘naturalized’ discharge provided by the state-of-the-art global integrated water model WaterGAP (model version 2.2 as of 2014). These data were spatially downscaled from their original 0.5 degree pixel resolution (~ 50 km at the equator) to the 15 arc-second (~ 500 m) resolution of the global HydroSHEDS river network using geo-statistical approaches.

To acquire individual lake volumes and watershed areas for the lakes, Messager et al.[6] compiled a bathymetric dataset. The bathymetric dataset was sourced from 14 existing national and international datasets which were compiled based on about 40 peer-reviewed articles. The watershed area data were from the bathymetric dataset. Data that were originally provided as bathymetric lines or in raster format were converted to lake volume using 3D GIS procedures.

Supplementary Note 12: The solution of the nutrient budget model

Mass balance equations (9 & 10) were derived from the previous study [7]; the solutions to the mass balance equations and the definitions of the symbols are presented in the main text. This model includes the terms representing contributors to lake eutrophication, and the most common strategies to mitigate eutrophication [8], such as watershed management practices (L_{N} and L_{P}), flushing (h), chemical immobilization of sediment P (N_{EN} and P_{EN}), and food web manipulation (N_{DE} and P_{DE}).

$$\frac{d\mathrm{TN}}{dt} = \frac{L_{\mathrm{N}}}{V} + N_{\mathrm{EN}} - N_{\mathrm{DE}} - h \times \mathrm{TN} \tag{9}$$

$$\frac{d\mathrm{TP}}{dt} = \frac{L_{\mathrm{P}}}{V} + P_{\mathrm{EN}} - P_{\mathrm{DE}} - h \times \mathrm{TP} \tag{10}$$

Direct discharges of P and N can be considered by adding a constant input to the mass balance equations. To focus on the deterministic framework of the nutrient budget model, previous studies consistently set shocks to zero. However, since the simulation results from NLA2012 datasets are available where the shocks are set as zero, new shocks to the same lakes reported in NLA2017 and NLA2022 cannot be ignored. If we set 2012 as the baseline, the nutrient budget model would change from a deterministic in-lake system to a dynamic system. Thus, we apply machine learning to generate f_{NAPI} and f_{NANI} to consider the impact of 2012 on 2017 and 2017 on 2022. Next, we use an algorithm to refine these shocks to normally distributed random variables. We first get mean value following a normal distribution with a large variance and 0 as initial mean value; then we observe the posterior distribution formed by the model after it is fitted. The posterior distributions from the last model iteration are used as the prior distributions for the next iteration. With enough iterations, change in the mean value will converge. The adjacent difference less than 5% was used as the termination condition.

Table S11 Prior distributions of parameters used in the nutrient budget model in 2012.

Symbol	Distribution	References
μ_A_N	Normal (30000, 3000)	[8]
σ_A_N	Normal (2000, 300)	[8]
μ_A_P	Normal (5000, 500)	[8]
σ_A_P	Normal (400, 100)	[8]
μ_B_N	Normal (1.3, 0.3)	[8]
σ_B_N	Normal (1.0, 0.1)	[8]
μ_B_P	Normal (2.1, 0.3)	[8]
σ_B_P	Normal (1.0, 0.1)	[8]
μ_M_N	Normal (12.0, 2.0)	[8]
σ_M_N	Normal (2.0, 1.0)	[8]
μ_M_P	Normal (2.0, 0.1)	[8]
σ_M_P	Normal (1.0, 0.1)	[8]
μ_S_N	Normal (0.001, 0.001)	[8]
σ_S_N	Normal (0.001, 0.01)	[8]
μ_S_P	Normal (0.01, 0.003)	[8]
σ_S_P	Normal (0.01, 0.003)	[8]
$\mu\_\theta_1^N$	Normal (0.50, 0.05)	[8]
$\sigma\_\theta_1^N$	Normal (0.05, 0.01)	[8]
$\mu\_\theta_2^N$	Normal (-0.05, 0.004)	[8]
$\sigma\_\theta_2^N$	Normal (0.010, 0.1)	[8]
$\mu\_\theta_3^N$	Normal (-0.02, 0.006)	[8]
$\sigma\_\theta_3^N$	Normal (0.010, 0.006)	[8]
$\mu\_\theta_4^N$	Normal (3.00, 0.2)	[8]
$\sigma\_\theta_4^N$	Normal (0.2, 0.1)	[8]
$\mu\_\theta_1^P$	Normal (0.23, 0.05)	[8]
$\sigma\_\theta_1^P$	Normal (0.1, 0.02)	[8]
$\mu\_\theta_2^P$	Normal (-0.01, 0.006)	[8]
$\sigma\_\theta_2^P$	Normal (0.011, 0.006)	[8]
$\mu\_\theta_3^P$	Normal (-0.02, 0.007)	[8]
$\sigma\_\theta_3^P$	Normal (0.010, 0.008)	[8]
$\mu\_\theta_4^P$	Normal (3.6, 0.5)	[8]
$\sigma\_\theta_4^P$	Normal (3.28, 0.13)	[8]
μ_θ_N	-	The model for 2012 sets 4 parameters to 0 since the initial model sets no shock.
σ_θ_N	-	
μ_θ_P	-	
σ_θ_P	-	

Table S12 Prior distributions of parameters used in the nutrient budget model in 2017.

Symbol	Distribution	References
μ_A_N	Normal (24486, 3000)	Posterior distribution of 2012
σ_A_N	Normal (2012, 300)	Posterior distribution of 2012
μ_A_P	Normal (3460, 500)	Posterior distribution of 2012
σ_A_P	Normal (423, 100)	Posterior distribution of 2012
μ_B_N	Normal (1.7, 0.3)	Posterior distribution of 2012
σ_B_N	Normal (1.0, 0.1)	Posterior distribution of 2012
μ_B_P	Normal (2.1, 0.3)	Posterior distribution of 2012
σ_B_P	Normal (1.0, 0.1)	Posterior distribution of 2012
μ_M_N	Normal (18.2, 2.0)	Posterior distribution of 2012
σ_M_N	Normal (4.0, 1.0)	Posterior distribution of 2012
μ_M_P	Normal (2.0, 0.1)	Posterior distribution of 2012
σ_M_P	Normal (1.0, 0.1)	Posterior distribution of 2012
μ_S_N	Normal (0.001, 0.001)	Posterior distribution of 2012
σ_S_N	Normal (0.001, 0.01)	Posterior distribution of 2012
μ_S_P	Normal (0.010, 0.003)	Posterior distribution of 2012
σ_S_P	Normal (0.01, 0.003)	Posterior distribution of 2012
$\mu\_\theta_1^N$	Normal (0.50, 0.05)	Posterior distribution of 2012
$\sigma\_\theta_1^N$	Normal (0.05, 0.01)	Posterior distribution of 2012
$\mu\_\theta_2^N$	Normal (-0.05, 0.004)	Posterior distribution of 2012
$\sigma\_\theta_2^N$	Normal (0.010, 0.1)	Posterior distribution of 2012
$\mu\_\theta_3^N$	Normal (-0.02, 0.006)	Posterior distribution of 2012
$\sigma\_\theta_3^N$	Normal (0.010, 0.006)	Posterior distribution of 2012
$\mu\_\theta_4^N$	Normal (3.00, 0.2)	Posterior distribution of 2012
$\sigma\_\theta_4^N$	Normal (0.2, 0.1)	Posterior distribution of 2012
$\mu\_\theta_1^P$	Normal (0.23, 0.05)	Posterior distribution of 2012
$\sigma\_\theta_1^P$	Normal (0.1, 0.02)	Posterior distribution of 2012
$\mu\_\theta_2^P$	Normal (-0.01, 0.006)	Posterior distribution of 2012
$\sigma\_\theta_2^P$	Normal (0.011, 0.006)	Posterior distribution of 2012
$\mu\_\theta_3^P$	Normal (-0.02, 0.007)	Posterior distribution of 2012
$\sigma\_\theta_3^P$	Normal (0.010, 0.008)	Posterior distribution of 2012
$\mu\_\theta_4^P$	Normal (3.6, 0.5)	Posterior distribution of 2012
$\sigma\_\theta_4^P$	Normal (3.28, 0.13)	Posterior distribution of 2012
μ_θ_N	Normal (0.38, 1)	Algorithm Generated
σ_θ_N	Normal (2.6, 1)	Algorithm Generated
μ_θ_P	Normal (0, 1)	Algorithm Generated
σ_θ_P	Normal (3.24,1)	Algorithm Generated

Table S13 Prior distributions of parameters used in the nutrient budget model in 2022.

Symbol	Distribution	References
μ_A_N	Normal (19530, 3069)	Posterior distribution of 2017
σ_A_N	Normal (2028, 298)	Posterior distribution of 2017
μ_A_P	Normal (1337, 315)	Posterior distribution of 2017
σ_A_P	Normal (455, 96)	Posterior distribution of 2017
μ_B_N	Normal (1.8, 0.3)	Posterior distribution of 2017
σ_B_N	Normal (1.0, 0.1)	Posterior distribution of 2017
μ_B_P	Normal (2.1, 0.3)	Posterior distribution of 2017
σ_B_P	Normal (1.0, 0.1)	Posterior distribution of 2017
μ_M_N	Normal (19.7, 2.0)	Posterior distribution of 2017
σ_M_N	Normal (4.0, 1.0)	Posterior distribution of 2017
μ_M_P	Normal (2.0, 0.1)	Posterior distribution of 2017
σ_M_P	Normal (1.0, 0.1)	Posterior distribution of 2017
μ_S_N	Normal (0.002, 0.001)	Posterior distribution of 2017
σ_S_N	Normal (0.002, 0.01)	Posterior distribution of 2017
μ_S_P	Normal (0.01, 0.003)	Posterior distribution of 2017
σ_S_P	Normal (0.01, 0.002)	Posterior distribution of 2017
μ_θ_1^N	Normal (0.35, 0.04)	Posterior distribution of 2017
σ_θ_1^N	Normal (0.06, 0.01)	Posterior distribution of 2017
μ_θ_2^N	Normal (-0.05, 0.004)	Posterior distribution of 2017
σ_θ_2^N	Normal (0.010, 0.1)	Posterior distribution of 2017
μ_θ_3^N	Normal (-0.02, 0.006)	Posterior distribution of 2017
σ_θ_3^N	Normal (0.01, 0.006)	Posterior distribution of 2017
μ_θ_4^N	Normal (2.7, 0.2)	Posterior distribution of 2017
σ_θ_4^N	Normal (0.3, 0.1)	Posterior distribution of 2017
μ_θ_1^P	Normal (0.19, 0.05)	Posterior distribution of 2017
σ_θ_1^P	Normal (0.1, 0.02)	Posterior distribution of 2017
μ_θ_2^P	Normal (-0.02, 0.006)	Posterior distribution of 2017
σ_θ_2^P	Normal (0.015, 0.006)	Posterior distribution of 2017
μ_θ_3^P	Normal (-0.02, 0.007)	Posterior distribution of 2017
σ_θ_3^P	Normal (0.010, 0.008)	Posterior distribution of 2017
μ_θ_4^P	Normal (3.6, 0.5)	Posterior distribution of 2017
σ_θ_4^P	Normal (3.28, 0.13)	Posterior distribution of 2017
μ_θ_N	Normal (0.41, 0.1)	Posterior distribution of 2017
σ_θ_N	Normal (0.18, 0.01)	Posterior distribution of 2017
μ_θ_P	Normal (0, 0.01)	Posterior distribution of 2017
σ_θ_P	Normal (0.01,0.01)	Posterior distribution of 2017

Table S14 Comparison of the indexes of two nutrient budget models (one with shocks and the other without shocks). We define that the absolute difference between indexes of two models divided by index with shocks less than 5% as the same; above 5% is different. MCSE (Monte Carlo standard error), STD (standard deviation) and ESS (effective sample size) are indexes of MCMC (Markov Chain Monte Carlo).

Parameters	Mean	MCSE	STD	ESS
μ_A_N	*different*	*different*	*same*	*different*
σ_A_N	*same*	*same*	*same*	*different*
μ_A_P	*different*	*different*	*different*	*different*
σ_A_P	*different*	*different*	*same*	*different*
μ_Q_N	*different*	*different*	*different*	*different*
σ_Q_N	*same*	*same*	*same*	*same*
μ_Q_P	*different*	*different*	*same*	*different*
σ_Q_P	*same*	*different*	*same*	*different*
μ_M_N	*different*	*different*	*different*	*different*
σ_M_N	*different*	*different*	*same*	*different*
μ_M_P	*same*	*different*	*same*	*different*
σ_M_P	*same*	*different*	*same*	*different*
μ_S_N	*different*	*different*	*different*	*different*
σ_S_N	*different*	*different*	*different*	*different*
μ_S_P	*different*	*same*	*different*	*different*
σ_S_P	*different*	*different*	*different*	*different*
$\mu\_\theta_1^N$	*different*	*same*	*different*	*different*
$\sigma\_\theta_1^N$	*same*	*different*	*different*	*different*
$\mu\_\theta_2^N$	*different*	*different*	*different*	*different*
$\sigma\_\theta_2^N$	*different*	*different*	*different*	*different*
$\mu\_\theta_3^N$	*different*	*different*	*different*	*different*
$\sigma\_\theta_3^N$	*same*	*different*	*same*	*different*
$\mu\_\theta_4^N$	*different*	*different*	*different*	*different*
$\sigma\_\theta_4^N$	*different*	*different*	*different*	*different*
$\mu\_\theta_1^P$	*different*	*different*	*different*	*different*
$\sigma\_\theta_1^P$	*different*	*different*	*same*	*different*
$\mu\_\theta_2^P$	*different*	*different*	*different*	*different*
$\sigma\_\theta_2^P$	*different*	*different*	*different*	*different*
$\mu\_\theta_3^P$	*same*	*different*	*different*	*different*
$\sigma\_\theta_3^P$	*different*	*different*	*different*	*different*
$\mu\_\theta_4^P$	*different*	*same*	*same*	*same*
$\sigma\_\theta_4^P$	*same*	*different*	*same*	*different*

(a) (b)

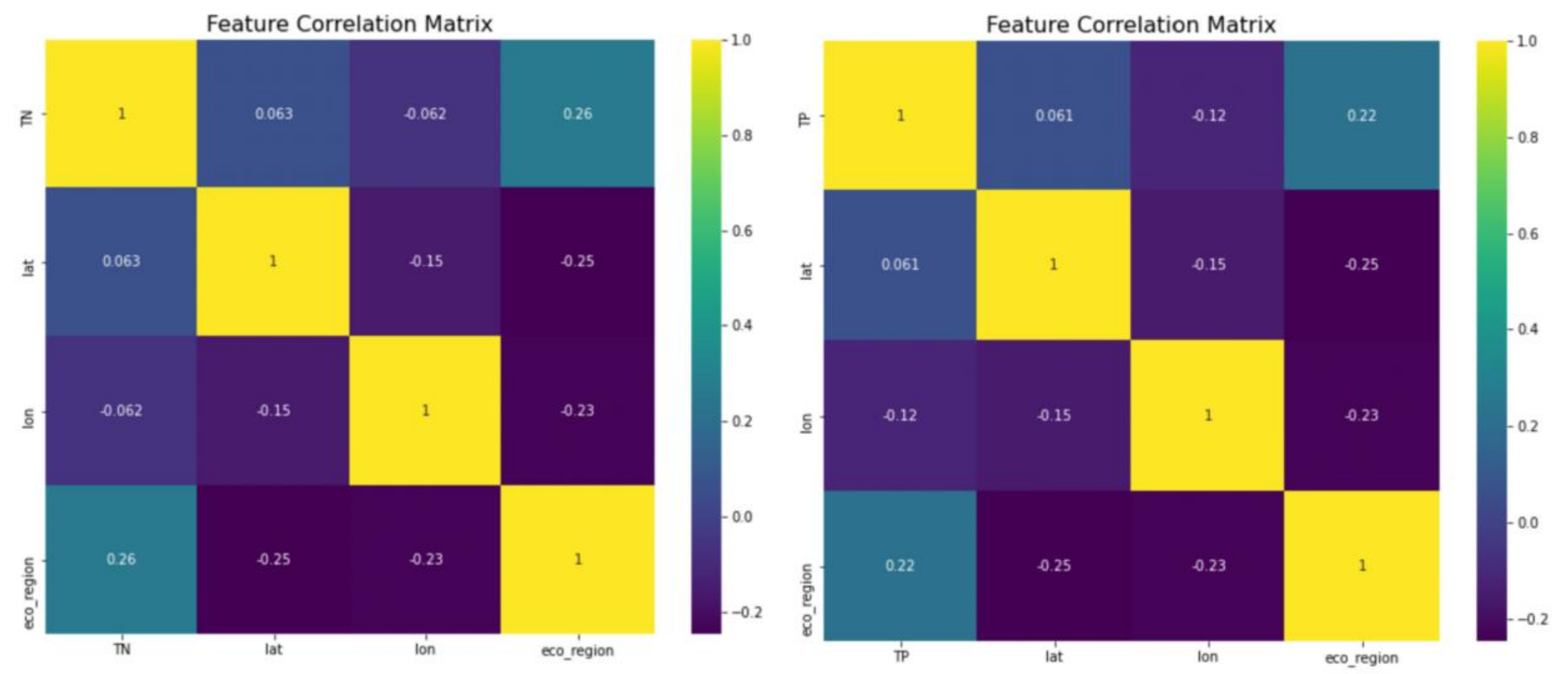

Figure S25 Feature correlation matrix of input parameters in machine learning generation of (a) f_{NANI} **and (b)** f_{NAPI}. The matrices show that the potential impact of auto-correlation among the independent variables has been ruled out. Source data are provided as a Source Data file.

References

[1] Beusen, A. H., Bouwman, A. F., Van Beek, L. P., Mogollón, J. M. & Middelburg, J. J. Global riverine N and P transport to ocean increased during the 20th century despite increased retention along the aquatic continuum. *Biogeosciences* 13 (8), 2441-2451 (2016).

[2] National Lakes Assessment 2022 (EPA, 2024).

[3] Lenton, T. M. Early warning of climate tipping points. *Nature climate change* 1(4), 201-209 (2011).

[4] Trauth, M. H. et al. Early warning signals of the termination of the African Humid Period (s). *Nature Communications* 15, 3697 (2024).

[5] Wang, R. et al. Flickering gives early warning signals of a critical transition to a eutrophic lake state. *Nature* 492(7429), 419-422 (2012).

[6] Messager, M. L., Lehner, B., Grill, G., Nedeva, I. & Schmitt, O. Estimating the volume and age of water stored in global lakes using a geo-statistical approach. *Nature Communications* 7, 13603 (2016).

[7] Wu, Z. et al. Imbalance of global nutrient cycles exacerbated by the greater retention of phosphorus over nitrogen in lakes. *Nature Geoscience* 15, 464 – 468 (2022).

[8] Carpenter, S.R. Eutrophication of aquatic ecosystems: bistability and soil P. *Proceedings of the National Academy of Sciences* 102 (29), 10002-10005, (2005).
