# Supplementary material for: Preferential nitrogen retention characterizes current eutrophication in United States lakes
Source: Nat Commun. 2026 Jul 9;17:8485. doi: 10.1038/s41467-026-75318-9 (PMC13478381; doi:10.1038/s41467-026-75318-9)
Supplement: Supplementary file 2 [file 41467_2026_75318_MOESM2_ESM.pdf]

# nature portfolio

Corresponding author(s): Xilong Wang

Last updated by author(s): May 18, 2026



# Reporting Summary

Nature Portfolio wishes to improve the reproducibility of the work that we publish. This form provides structure for consistency and transparency in reporting. For further information on Nature Portfolio policies, see our Editorial Policies and the Editorial Policy Checklist.

## Statistics

For all statistical analyses, confirm that the following items are present in the figure legend, table legend, main text, or Methods section.

| n/a | Confirmed | |
|---|---|---|
| ☐ | ☒ | The exact sample size (*n*) for each experimental group/condition, given as a discrete number and unit of measurement |
| ☐ | ☒ | A statement on whether measurements were taken from distinct samples or whether the same sample was measured repeatedly |
| ☐ | ☒ | The statistical test(s) used AND whether they are one- or two-sided<br>*Only common tests should be described solely by name; describe more complex techniques in the Methods section.* |
| ☐ | ☒ | A description of all covariates tested |
| ☐ | ☒ | A description of any assumptions or corrections, such as tests of normality and adjustment for multiple comparisons |
| ☐ | ☒ | A full description of the statistical parameters including central tendency (e.g. means) or other basic estimates (e.g. regression coefficient) AND variation (e.g. standard deviation) or associated estimates of uncertainty (e.g. confidence intervals) |
| ☐ | ☒ | For null hypothesis testing, the test statistic (e.g. *F*, *t*, *r*) with confidence intervals, effect sizes, degrees of freedom and *P* value noted<br>*Give P values as exact values whenever suitable.* |
| ☐ | ☒ | For Bayesian analysis, information on the choice of priors and Markov chain Monte Carlo settings |
| ☐ | ☒ | For hierarchical and complex designs, identification of the appropriate level for tests and full reporting of outcomes |
| ☐ | ☒ | Estimates of effect sizes (e.g. Cohen's *d*, Pearson's *r*), indicating how they were calculated |

*Our web collection on statistics for biologists contains articles on many of the points above.*

## Software and code

Policy information about availability of computer code

| | |
|---|---|
| Data collection | No software is used in data collection. The datasets are obtained from National lakes assessments. |
| Data analysis | Bayesian inference for the in-lake budget model was performed in Julia via the CmdStan software. All spatiotemporal data analysis and visualization were implemented in Python. The key code developed in the study is at Figshare(https://doi.org/10.6084/m9.figshare.32297847). |

For manuscripts utilizing custom algorithms or software that are central to the research but not yet described in published literature, software must be made available to editors and reviewers. We strongly encourage code deposition in a community repository (e.g. GitHub). See the Nature Portfolio guidelines for submitting code & software for further information.

## Data

Policy information about availability of data

All manuscripts must include a data availability statement. This statement should provide the following information, where applicable:

- Accession codes, unique identifiers, or web links for publicly available datasets
- A description of any restrictions on data availability
- For clinical datasets or third party data, please ensure that the statement adheres to our policy

All data that support the findings in the current study are available within the Article and its Supplementary Information. Source data including USL dataset, the fnani&NAPI data and INCI data are available at Figshare (https://figshare.com/s/481c9bef67a527de5ae8).

## Research involving human participants, their data, or biological material

Policy information about studies with human participants or human data. See also policy information about sex, gender (identity/presentation), and sexual orientation and race, ethnicity and racism.

| | |
|---|---|
| Reporting on sex and gender | This study does not involve this section. |
| Reporting on race, ethnicity, or other socially relevant groupings | This study does not involve this section. |
| Population characteristics | This study does not involve this section. |
| Recruitment | This study does not involve this section. |
| Ethics oversight | This study does not involve this section. |

Note that full information on the approval of the study protocol must also be provided in the manuscript.

# Field-specific reporting

Please select the one below that is the best fit for your research. If you are not sure, read the appropriate sections before making your selection.

☐ Life sciences ☐ Behavioural & social sciences ☒ Ecological, evolutionary & environmental sciences

For a reference copy of the document with all sections, see nature.com/documents/nr-reporting-summary-flat.pdf

# Ecological, evolutionary & environmental sciences study design

All studies must disclose on these points even when the disclosure is negative.

| | |
|---|---|
| Study description | This study adopts a stepwise approach. First, multi-source data are integrated to build the USL dataset. Next, drawing on this dataset and the fnani/fnapi test set, a range of machine-learning methods are systematically evaluated; the best-performing algorithms are selected to generate (fit) the fnani/fnapi data. Subsequently, a Bayesian hierarchical model is constructed that takes both the USL dataset and the fnani/fnapi data as inputs to model and predict the target variable. Throughout the entire workflow, the Bayesian framework is used to quantify and propagate uncertainty at every stage. |
| Research sample | 121 lakes in the USA were screened out and a panel dataset named United States Lakes (USL) dataset was compiled. |
| Sampling strategy | Three datasets of 121 lakes were screened out on the basis of the intersection of the NLA datasets of 2012, 2017 and 2022 according to HUC8. |
| Data collection | All data were collected by the authors. |
| Timing and spatial scale | All data on nutrient dynamics of 121 lakes in the USA were collected across three sampling years (2012, 2017, and 2022) |
| Data exclusions | Data were NOT excluded. |
| Reproducibility | All attempts to repeat the model simulations were successful. |
| Randomization | Samples were not randomly assigned. Instead, 121 lakes were classified into ten regions according to the USEPA Level III and IV Ecoregion framework. Lakes lying within the same ecoregion share a common set of sub-parameters in the Bayesian hierarchical model, a design chosen to facilitate model fitting and to reduce predictive uncertainty rather than to achieve random allocation across groups. |
| Blinding | Blinding was not possible in our study, because our research did not involve randomized controlled clinical trials. |

Did the study involve field work? ☐ Yes ☒ No

# Reporting for specific materials, systems and methods

We require information from authors about some types of materials, experimental systems and methods used in many studies. Here, indicate whether each material, system or method listed is relevant to your study. If you are not sure if a list item applies to your research, read the appropriate section before selecting a response.

## Materials & experimental systems

| n/a | Involved in the study |
| --- | --- |
| ☒ | ☐ Antibodies |
| ☒ | ☐ Eukaryotic cell lines |
| ☒ | ☐ Palaeontology and archaeology |
| ☒ | ☐ Animals and other organisms |
| ☒ | ☐ Clinical data |
| ☒ | ☐ Dual use research of concern |
| ☒ | ☐ Plants |

## Methods

| n/a | Involved in the study |
| --- | --- |
| ☒ | ☐ ChIP-seq |
| ☒ | ☐ Flow cytometry |
| ☒ | ☐ MRI-based neuroimaging |

# Plants

| | |
| --- | --- |
| Seed stocks | This study does not involve plant. |
| Novel plant genotypes | This study does not involve plant. |
| Authentication | This study does not involve plant. |
