# Supplementary material for: Preferential nitrogen retention characterizes current eutrophication in United States lakes
Source: Nat Commun. 2026 Jul 9;17:8485. doi: 10.1038/s41467-026-75318-9 (PMC13478381; doi:10.1038/s41467-026-75318-9)
Supplement: Supplementary file 3 [file 41467_2026_75318_MOESM3_ESM.pdf]

nature portfolio

Peer Review File

Preferential nitrogen retention characterizes current eutrophication in United States lakes

Corresponding Author: Professor Xilong Wang

This file contains all reviewer reports in order by version, followed by all author rebuttals in order by version.

Version 0:

Reviewer comments:

Reviewer #1

(Remarks to the Author)
Let me start by saying that I have not reviewed the details of the modeling. I have reviewed the dominant text and the interpretations. Overall, this is an important and provocative paper, with important implications for understanding and managing eutrophication. While this analysis is centered on US lakes, it has relevance for lakes worldwide, which will face the same shifts in nutrient dynamics and trophic state as eutrophication progresses.
The paper does need English editing. While I have made some line by line comments below, it would benefit from a more thorough English polishing.
Detailed edits:
Line 24. A previous study…
Line 25, suggested edit: and weakens the argument for N reduction. (delete rests of sentence that goes on to line 26)
Line 40, remove although
Lines 41-42, suggested edit: ….80% of P, and together with a series of P control…some eutrophication alleviation in some countries in the Northern Hemisphere, but lake …
Line 44. Replace It with This phenomenon
Line 45. Suggested edit: lake eutrophication continued to exacerbate
Line 48 replace since to in the
Line 55 replace dominantly with predominantly
Lines 57 to 58 use em dashes so that it reads within–and natural losses from–lakes
Line 62 what is meant by algal excretion? Only used in the figure legend and never mentioned/defined in text
Line 65- awkward phrasing…why is this here?
Line 67 suggested edit: …are both pollution and since many have been remediated, they are at the forefront…
Line 69, replace they with managers
Line 73 insert may after subsequently
Line 77 insert commas around yet currently scarce
Line 79 remove the words creatively and
Line 80 remove the word out
Lines 82-88, suggested edits to make these statements into questions.
Line 81 end sentence after 2022).
Line 82 New sentence We employed…
Line 82 rephrase 1) To what extent has preferential retention of P shifted to N…
Line 85, change to change significantly. Also change whether to have substantial
Line 86 remove has
Line 87 replace Whether with Has a new wave…remove second has in the sentence
Line 89 change to above mentioned
Line 90 employed
Line 104 remove out
Line 142 sharply, not shapely
Line 144 insert the before number
Line 149 replace severer with more severe
Line 171 replace that to in which
Line 178. Do you mean percent rather than the number of? Note that letters in the figure legend do not match the figure

panels.
Lines 188-190 Suggested edit: This phenomenon has been shown in models….eutrophicaiton. (delete most of line 189) These shifts align with the suite of characteristics of flickering, rather than with interannual variability…
Line 195 do you mean of flickering? Note that the dots on the maps are virtually impossible to see
Line 201 …in the USA was in a state of
Line 202 to that which occurs
Line 212 remained
Line 212 and ALL SUBSEQUENT uses of the term MNMD like- replace with NMD-like
Line 213 remove the before nutrient
Line 217 same comment about figure 5—dots on the maps cannot be seen
Line 223 suggested edit: US lakes have been at the forefront …century, demonstrating a slow, yet steady, growth of eutrophication from 2012-2022
Line 225 Yet, there has been no significant
Line 226 remove s on literature
Line 228 remove It could be that…, and start sentence with Over
Line 233 weakens not weakened
Line 236 insert trend after eutrophication
Line 244 Text- especially in colored box in Figure 6 too small to read
Line 313 insert has before initiated
(from p. 14 methods section- not edited)

(Remarks on code availability)

Reviewer #2

(Remarks to the Author)
Review of "Eutrophication Dynamics: The Shift of Preferential Nutrient Retention and the Era of Nutrient Metabolic Disorder" by Zhou et al.

Line 83: It is also possible that the imposed P controls themselves shifted the N:P stoichiometry, which then pushed the system towards this regime shift (although it is questionable, and links directly to the points I mentioned in my comments below). In that case, both N and P controls would have been important at the same time.

Lines 111 to 119: It looks like the authors intended to refer to Figure 2d, because Figure 2a shows log-scaled enrichment and retention ratios for N and P, which are not the same as percentage changes in preferential retention. None of the panels in 2a, 2b or 2c actually present the percentage increase or decrease that the text is describing.

Lines 112 to 117: This whole section needs to be rewritten with clearer statements and the correct figure references. The figure numbers do not match the text, and the caption itself is confusing. For example, it says "Lakes located beyond the black line show a pattern of preferential N retention", but there are two different black lines in the plot, the dotted 1:1 line and the solid axes. "Beyond" is ambiguous. The authors should explicitly state which region or quadrant they mean. In the same place, the authors also need to clearly explain how the enrichment-to-depletion ratio relates to nutrient retention and why it is inversely proportional, following Wu et al. Without this clarification, the reader will simply be confused.

Line 146: The claim that "the preference of N retention has suppressed the net P retention in most lakes" is not supported by the evidence. Even if $N^{+}P^{-}$ lakes increased, that does not show that N retention caused a decline in P retention, nor does it demonstrate a suppression mechanism. The manuscript is inferring causality from a pattern, and the logic is pushed too far beyond what the data actually show.

Statement related to Figure 3: The decline in net retention with increasing trophic level is a well-known feature of eutrophic systems. It simply means that more eutrophic lakes retain nutrients less effectively. This does not establish the causal link that the authors are implying, that preferential N retention is suppressing P retention. Needs explanation and justification.

Line 163–170: The authors use the Wu et al. algorithm to calculate INCI, but Fig. 3 only shows percentages of lakes based on INCI categories. In Supplementary Text 4, they only show external loading and inflow statistics for N and P. I would like to actually see the INCI values themselves. These should be in the supplementary material so the reader gets a clearer picture of what the model is producing.

Line 175–176: I am not sure how the authors arrive at "This consistently suggests that the influence of internal cycling on net nutrient retention also has an upper limit". What is this upper limit? The sentence needs more explanation before making that conclusion.

Supplementary Text 5, Line 371–372: To rule out interannual variability, the authors need to present a proper continuous time series for at least a decade or more. If they want to justify flickering or tipping-point behaviour, they need to show high-frequency switching, noise-driven exits and returns to the attractor. None of this can be shown with only three snapshots.

Three years of data are not enough to call it flickering.

Supplementary Text 5, Line 364–365: The statement that “USEPA reports show 10-year nutrient input data remain largely stable” does not rule out extreme events or short-term spikes, which could be important. This again points to the need for a long-term dataset.

Supplementary Text 5, Line 398–400: Again, I cannot see the actual INCI results anywhere in the manuscript.

Figure 4: Needs improvement. The points and colours are barely visible.

Line 200–215: Fig. 5a shows P retention higher than N in 2012, which matches the model’s outflow N/P being above the 1:1 line, meaning more N is released than P. But in 2017 and 2022 (Fig. 5b and 5c), when NDE/PDE is higher (points above the 1:1 line), meaning N should be retained more than P, I would expect to see the same trend in the inflow–outflow plots. Instead, the outflow plots still show more N above the 1:1 line, meaning more N release than P, which is the opposite. I am not convinced how the authors conclude this is “anomalous”. The model and the observations are not aligned. And if the authors think this reflects ongoing eutrophication, they should show evidence for that.

Line 248–249: I understand Extra Trees performs better than the other ML methods for f_NAPI, but with $R^2 = 0.53$ and MSE = 6.92, it is not strong enough to claim the final model has “lower uncertainty”. This is also evident in figure S18(b) with overestimation of f_NAPI at lower actual value. The unexplained variance is high and feeding this into the Bayesian model risks introducing systematic bias, since the ML-based external loads (f_NAPI) are not well constrained.

Overall: The study tackles an important problem, and the dataset is valuable. The attempt to use a Bayesian nutrient-budget model alongside ML is interesting, and the focus on internal cycling is a genuinely useful angle. But the manuscript also stretches its conclusions well beyond what the data can support. Three snapshot years cannot justify claims about flickering, regime shifts, suppression mechanisms, or systems “adjusting nutrient preferences”. Several key definitions are unclear, figure references don’t line up, and important variables like INCI are not clear to me. The ML models feeding the Bayesian core have modest skill, yet the manuscript repeatedly claims “extremely low uncertainty”, which is not consistent with the model performance. In a few places, the narrative simply over-interprets patterns as causation. The idea has potential, but the conclusions need to be far more cautious and grounded in what the data can actually demonstrate.

(Remarks on code availability)
I was not able to access the code because the repository says ‘files are restricted to users with access’ and requires a username and email. That would break reviewer anonymity, so I could not check the code.

Version 1:

Reviewer comments:

Reviewer #1

(Remarks to the Author)
Thank you for the revisions. My comments below are minor editorial corrections:

line 68 change to have been polluted, not pollution
lines 81-82 change to screened, compiled, analyzed
line 87 remove whether
line 245 remove This reminds that
line 350 remove Moreover

(Remarks on code availability)

Reviewer #2

(Remarks to the Author)
Thank you for the careful revision and detailed response. The manuscript has improved substantially compared with the previous version, and the authors have made a genuine effort to address the reviewers’ concerns. The topic is interesting and potentially important for the field, particularly given the broad implications for understanding nutrient dynamics and eutrophication management in lakes.

In my view, the revision has clearly strengthened the manuscript in several useful ways. The presentation is improved, a number of earlier ambiguities have been clarified, and the additional analyses and source data deposition have made the study more transparent. I also appreciate that the authors have moderated some of the earlier language. That said, I think the manuscript would still benefit from one further tightening of the interpretation in a few key places. In particular, the language around flickering-like dynamics, critical transitions, and regime shift should be kept consistently cautious throughout. With only three time points, the data may support the idea of patterns consistent with instability, but not a firm diagnosis of flickering or a regime shift in the stronger sense. I would therefore recommend revising these statements so that they are

framed explicitly as a plausible interpretation or hypothesis supported by the observed patterns, rather than as a conclusion that has already been demonstrated. That would make the paper more rigorous and, frankly, harder to shoot down.

A similar point applies to the discussion of the relationship between preferential N retention and suppression of net P retention. The revised analyses do improve this part, but the manuscript should still avoid language that sounds more causal than the evidence allows. I suggest that the authors consistently use wording such as associated with, consistent with, or may contribute to, unless they can provide stronger evidence for causality. This is not just semantics; it matters because the current formulation risks overstating what the analysis can actually establish. A slightly more careful framing would preserve the novelty of the result while keeping the interpretation on solid ground.

On reproducibility, the provision of source datasets and Stan model files is helpful and addresses the earlier access issue. However, if the authors wish to claim that the study can be reproduced from the deposited materials, I would encourage them to provide the fuller computational workflow as well, including the scripts used for preprocessing, model execution, and generation of derived outputs and figures, or otherwise moderate the wording of the code availability statement accordingly.

Overall, I find the manuscript improved and potentially publishable, but I believe one further round of tightening would strengthen it considerably. The main concern now is a more disciplined alignment between the strength of the claims and the actual strength of the evidence.

(Remarks on code availability)
I reviewed the deposited materials on Figshare, but in my view they do not yet provide a fully reproducible workflow for the study. The repository appears to include the Stan model files and processed datasets, which is helpful, but it does not seem to contain the full computational pipeline needed to reproduce the results end to end. In particular, I could not identify a clear README, execution instructions, preprocessing scripts, machine-learning workflow for generating fNANI/fNAPI, the scripts used to run the Bayesian models, or the code used to derive the reported outputs and figures. As such, the deposit is useful for inspection of model structure and input data, but not sufficient for full reproducibility in practice. I was therefore not able to verify the results by running the code directly. In its current form, I would not regard the code as a readily usable community resource.

Open Access This Peer Review File is licensed under a Creative Commons Attribution 4.0 International License, which permits use, sharing, adaptation, distribution and reproduction in any medium or format, as long as you give appropriate credit to the original author(s) and the source, provide a link to the Creative Commons license, and indicate if changes were made.
In cases where reviewers are anonymous, credit should be given to 'Anonymous Referee' and the source.
The images or other third party material in this Peer Review File are included in the article’s Creative Commons license, unless indicated otherwise in a credit line to the material. If material is not included in the article’s Creative Commons license and your intended use is not permitted by statutory regulation or exceeds the permitted use, you will need to obtain permission directly from the copyright holder.
To view a copy of this license, visit https://creativecommons.org/licenses/by/4.0/

Responses (manuscript ID: NCOMMS-25-70712) to Reviewers:

We sincerely thank all reviewers for their constructive comments and thoughtful revision suggestions on our manuscript. These insights have been invaluable and have significantly enhanced the quality and clarity of our work. We have carefully revised the manuscript and provided detailed point-to-point responses to adequately address the concerns raised by all reviewers.

The comments are in green color, and the revised text that appears in the Main Text and Supplementary Information is in blue color.

Reviewer #1 :

Let me start by saying that I have not reviewed the details of the modeling. I have reviewed the dominant text and the interpretations. Overall, this is an important and provocative paper, with important implications for understanding and managing eutrophication. While this analysis is centered on US lakes, it has relevance for lakes worldwide, which will face the same shifts in nutrient dynamics and trophic state as eutrophication progresses.

The paper does need English editing. While I have made some line by line comments below, it would benefit from a more thorough English polishing.

【Response】 *Thank you so much for your positive comments and revision suggestions. We have carefully revised the manuscript closely following your constructive comments and revision suggestions, which highly improved the quality and clarity of our manuscript.*

Detailed edits:

1.Line 24. A previous study…

【Response】Thanks for your edit. The sentence has been revised as shown below. *Please see lines 23-24 for revisions in the text.*

A previous study indicates that almost 90% of global lakes show preferential retention of P[3].

2. Line 25, suggested edit: and weakens the argument for N reduction. (delete rests of sentence that goes on to line 26)

【Response】Thanks for your edit. The sentence has been revised as shown below. *Please see lines 24-25 for revisions in the text.*

This further fuels the long-standing debate for prioritization of P pollution mitigation and weakens the argument for N reduction[5-7].

3. Line 40, remove although
Lines 41-42, suggested edit: ….80% of P, and together with a series of P control…some eutrophication alleviation in some countries in the Northern Hemisphere, but lake …

【Response】Thanks for your edit. The sentence has been revised as suggested as shown below. *Please see lines 39-44 for revisions in the text.*

Meanwhile, wastewater treatment plants generally retain approximately 60% of N and 80% of P[16], and together with a series of P control measures applied between the 1970s and 1990s did achieve some eutrophication alleviation in some countries in the Northern Hemisphere[10], but lake eutrophication in these countries staged a comeback again in the last 20 years (defined as a new wave of eutrophication since 21st century[17]).

4. Line 44. Replace It with This phenomenon
Line 45. Suggested edit: lake eutrophication continued to exacerbate

【Response】Thanks for your edit. The sentence has been revised as suggested as shown below. *Please see lines 47-48 for revisions in the text.*

This phenomenon is especially significant in countries such as USA where P control has become the dominant view; lake eutrophication continued to exacerbate[18, 19].

5. Line 48 replace since to in the

【Response】 Thanks for your correction. The sentence has been revised as suggested as shown below. *Please see lines 47-48 for revisions in the text.*

No consensus on the underlying causes responsible for the new wave of eutrophication has been reached in the 21st century[21, 22].

6. Line 55 replace dominantly with predominantly

【Response】Thanks for your edit. The sentence has been revised as suggested as shown below. *Please see lines 54-56 for revisions in the text.*

Additionally, whether the nutrient in lakes is predominantly limited by N or P depends on the in-lake nutrient dynamics[25].

7. Lines 57 to 58 use em dashes so that it reads within–and natural losses from–lakes

【Response】Thanks for your edit. The sentence has been revised as suggested as shown below. Please see lines 56-58 for revisions in the text.

Thus, understanding of internal nutrient cycling that includes in-lake nutrient enrichment and depletion[3, 26] and nutrient retention which includes natural internal retention within– and natural losses from– lakes[27] is also considered essential to eutrophication mitigation (Fig. 1).

8. Line 62 what is meant by algal excretion? Only used in the figure legend and never mentioned/defined in text
Line 65- awkward phrasing…why is this here?

【Response】We appreciate the reviewer's careful reading. The term "algae excretion" was originally intended to broadly describe the return of nutrients to the water column following algal uptake, including nutrient regeneration associated with algal metabolism, senescence, and biomass turnover. We agree that this wording may be unclear and potentially misleading. We have therefore revised the manuscript to replace this term with "**nutrient regeneration associated with algal biomass turnover**". We note that the manuscript does not further elaborate on this process in the main text because the objective of this study is not to resolve or quantify individual sub-processes of internal nutrient cycling. Instead, in-lake nutrient enrichment and depletion are treated at an aggregated level in the model framework. The reference to specific mechanisms is provided only to conceptually illustrate the meaning of internal nutrient regeneration and does not imply that these processes are explicitly represented or analyzed in the model.

We thank the reviewer for the comment regarding *Line 65*. This explanation for nutrient retention was included in the figure caption because the concept of nutrient retention is explicitly illustrated in the figure and the caption was intended to make the figure self-explanatory. However, we agree that the original wording was overly detailed for a figure caption and partially overlapped with the main text. We have therefore revised the caption to simplify the definition.

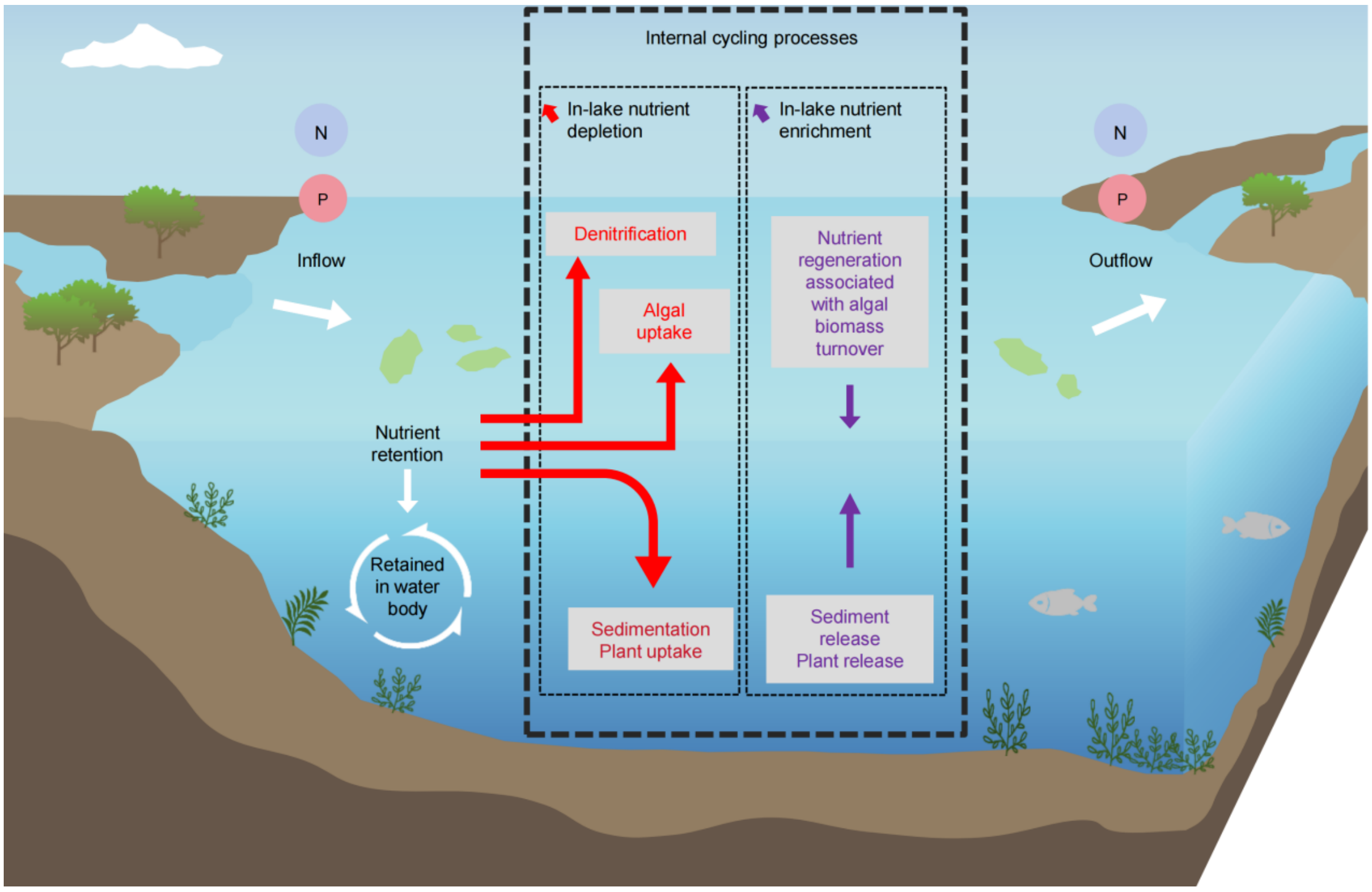

Fig. 1 A diagram showing the nutrient (N and P) cycling processes and nutrient retention. The internal nutrient

cycling processes include two processes: (1) in-lake nutrient enrichment, which mainly refers to input of nutrients from sediment release and algae excretion, and (2) in-lake nutrient depletion, which means removal of a part of existing nutrient and that retained from inflows by denitrification, sedimentation, and algae uptake. Nutrient retention includes nutrients retained in the water body and those removed through in-lake depletion. Net nutrient retention is derived from nutrient retention by excluding the contribution of in-lake nutrient enrichment.

9. Line 67 suggested edit: …are both pollution and since many have been remediated, they are at the forefront…

【Response】Thanks for your edit. Following your suggestion, the statement "The lakes in the USA are both polluted and remediated early; they are at the forefront of the development history of lake pollution remediation in the world" has been revised as shown below. *Please see lines 68-70 for revisions in the text.*

The lakes in the USA are both pollution and since many have been remediated, they are at the forefront of the development history of lake pollution remediation in the world.

10. Line 69, replace they with managers

【Response】 Following your suggestion, the statement "Given that prioritization of P mitigation has been recognized by policy makers, they have to rely on traditional means such as reducing excess external nutrient loading, sediment dredging, aeration of the hypolimnetic water and in situ chemical inactivation to control P[28,29,30] based on the cognition of the old paradigm of eutrophication." has been revised as shown below. *Please see lines 70-71 for revisions in the text.*

Given that prioritization of P mitigation has been recognized by policy makers, managers have to rely on traditional means such as reducing excess external nutrient loading, sediment dredging, aeration of the hypolimnetic water and in situ chemical inactivation to control P[28,29,30] based on the cognition of the old paradigm of eutrophication.

11. Line 73 insert may after subsequently

【Response】 Thanks for your edit. A word "may" has been added after subsequently as suggested. *Please see lines 73-75 as shown below for revisions in the text.*

Notably, the old paradigm is conventionally derived from statistical analysis but lacks continuous monitoring of the same lakes[31, 32], which is problematic or even misleading and subsequently may lead to formulation of non-tailored and ineffectively targeted restoration strategies.

12. Line 77 insert commas around yet currently scarce

【Response】Thanks for your edit. A comma has been inserted between scale and since. *Please see lines 78-81 as shown below for revisions in the text.*

Definitely, a more preferable yet currently scarce approach is to conduct one-to-one tracking of the nutrient status of lakes on a large scale, since the geophysical, nutrient, and landscape indicators among different lakes exhibit substantial and pronounced variations[35].

13. Line 79 remove the words creatively and
Line 80 remove the word out
Line 81 end sentence after 2022).
Line 82 New sentence We employed…

【Response】Thanks for your edits. Following your suggestion, the words "creatively and" and "out" in the sentence have been deleted, and the sentence was changed to end after 2022. The last part of the sentence was changed to start from we employed. *Please see lines 81-83 as shown below for revisions in the text.*

Here we systematically screen 121 lakes in the USA and compile a panel dataset named United States Lakes (USL) dataset, and analyze their nutrient dynamics across three sampling years (2012, 2017, and 2022). We employed a cohort-style tracking study aiming to address the following knowledge gaps.

14. Lines 82-88, suggested edits to make these statements into questions.
Line 82 rephrase 1) To what extent has preferential retention of P shifted to N…
Line 85, change to change significantly. Also change whether to have substantial
Line 86 remove has
Line 87 replace Whether with Has a new wave…remove second has in the sentence

【Response】Thanks for your suggestion and detailed edits. Following your suggestion, these statements have been revised as shown below. *Please see lines 84-90 for revisions in the text.*

1) To what extent has preferential retention of P shifted to N over the past decade, thereby influencing the effectiveness of P control strategies? 2) Given that the external loading that may influence eutrophication of these lakes did not change significantly over the past decade[20], whether have substantial changes in net N and P retention occurred? If so, has the net nutrient retention change in these lakes been dominated by external loading or internal cycling? and 3) Has a new wave of eutrophication occurred in the lakes and they are undergoing a regime shift, rather than just an ordinary recurrence of eutrophication?

15. Line 89 change to above mentioned
Line 90 employed

【Response】 Thanks for your revision suggestion and edit. Following your suggestion, the sentence has been revised as shown below. *Please see lines 91-93 for revisions in the text.*

To systematically address these knowledge gaps based upon the above mentioned datasets, we employed a progressive approach by combining machine learning and Bayesian hierarchical method to track changes in nutrient retention in 121 lakes from 2012 to 2022.

16. Line 104 remove out

【Response】 Thanks for your edit. The word "out" has been deleted. *Please see lines 106-107 as shown below for revisions in the text.*

The nutrient retention pattern in 121 lakes that were screened for tracking in the USA was verified using their datasets (see Methods, USL data).

17. Line 142 sharply, not shapely

【Response】 Thanks for your correction. The word "shapely" has been corrected as "sharply". *Please see lines 147-148 as shown below for the correction in the text.*

However, Finlay et al.[42] found that steep declines of P availability and productivity made N concentrations sharply rise.

18. Line 144 insert the before number

【Response】 A word "the" has been inserted before the word "number". *Please see lines 147-150 as shown below for revisions in the text.*

By tracking the nutrient dynamics of 121 lakes in the USA, we find that accompanying the shift in dominance of the preferential nutrient retention from P to N, the number of the N+P- group lakes significantly increased while that of the N-P+ group lakes significantly decreased (Fig. 2e, 2f & 2g).

19. Line 149 replace severer with more severe

【Response】Thanks for your correction. The word "severer" has been corrected as "more severe". *Please see lines 156-159 as shown below for revisions in the text.*

The proportion of lakes with positive net P and N retention generally rapidly decreases as trophic levels increase (Fig. 3), indicating a negative feedback of these two nutrients and the lakes with more severe pollution are more unfavorable to their net retention.

20. Line 171 replace that to in which

【Response】The word "that" has been revised as "in which" as suggested. *Please see lines 176-178 as shown below for revisions in the text.*

The number of lakes at a given trophic level in which their net N and P retention was predominantly regulated by internal cycling generally showed an initial increase followed by a decrease from 2012 to 2022 (Fig. 3).

21. Line 178. Do you mean percent rather than the number of? Note that letters in the figure legend do not match the figure panels.

【Response】 Thanks for your correction. Yes. We mean percentage here. To avoid confusion, we have changed the word “number” to “percentage” to keep consistent with the figure panels. Please see the revised figure caption for Fig. 3 as shown below for revisions.

Fig. 3 The percentage changes of the lakes that exhibited net N (P) retention and internal dominant model.

22. Lines 188-190 Suggested edit: This phenomenon has been shown in models….eutrophicaiton. (delete most of line 189) These shifts align with the suite of characteristics of flickering, rather than with interannual variability…

【Response】 Thanks for your revision suggestion and edit. The sentences have been revised and shortened as shown below. *Please see lines 194-196 for revisions in the text.*

This phenomenon has been shown in models of trophic cascades and eutrophication. These shifts align with the suite of characteristics of flickering, rather than with interannual variability, diffusion of nutrients, and data distortion (Supplementary Text 6 & 7).

23. Line 195 do you mean of flickering? Note that the dots on the maps are virtually impossible to see

【Response】Yes. Here, we mean flickering of key metrics. We have clarified this and enlarged the dots on the map. *Please see the revised Fig. 4 for revisions.*

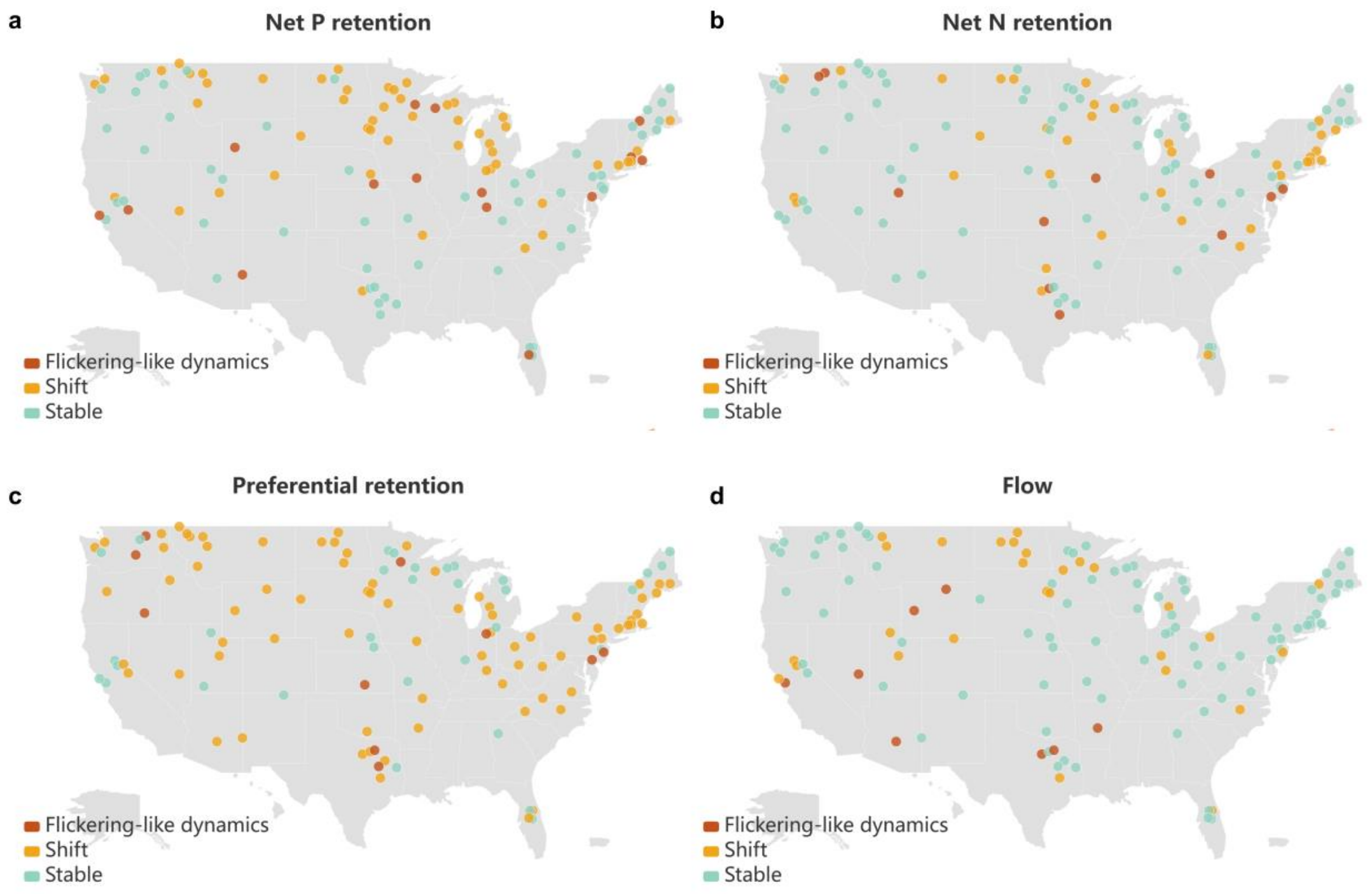

Fig. 4 Spatial distribution of the flickering-like dynamics of key metrics. The types can be categorized based on the number of shifts as: 2 shifts (flickering-like dynamics), 1 shift (shift), and no shift (stable). Four graphs show flickering-like pattern of **(a)** net P retention switching between positive and negative, **(b)** net N retention switching between positive and negative, **(c)** preferential nutrient retention switching between N and P, **(d)** flow N/P switching between inflow N/P over outflow N/P and outflow N/P over inflow N/P. Source data are provided as a Source Data file.

24. Line 201 …in the USA was in a state of
    Line 202 to that which occurs

【Response】Thanks for your edits. The sentences have been clarified as shown below. *Please see lines 206-210 for revisions in the text.*

Consistent with the theoretical expectation associated with flickering dynamics, our results further reveal that between 2012 and 2017 the nutrient dynamics of lakes across the USA underwent a pronounced and system-wide shift, exhibiting anomalous characteristics consistent with a nutrient metabolic disorder-like (NMD-like) pattern (Fig. 5g - 5i).

25. Line 212 remained
Line 212 and ALL SUBSEQUENT uses of the term MNMD like- replace with NMD-like
Line 213 remove the before nutrient

【Response】 Thanks for your edits. The word “remains” was corrected as “remained” and “the” before nutrient was removed. All terms “NMD like” throughout the main text were revised as “NMD-like”. *Please see lines 206-221, 218-219 for revisions in the text.*

As a result, this peculiar phenomenon remained undetected for a long time.

26. Line 217 same comment about figure 5—dots on the maps cannot be seen

【Response】The dots on the map have been enlarged as suggested. *Please see the revised Fig. 5 for revisions.*

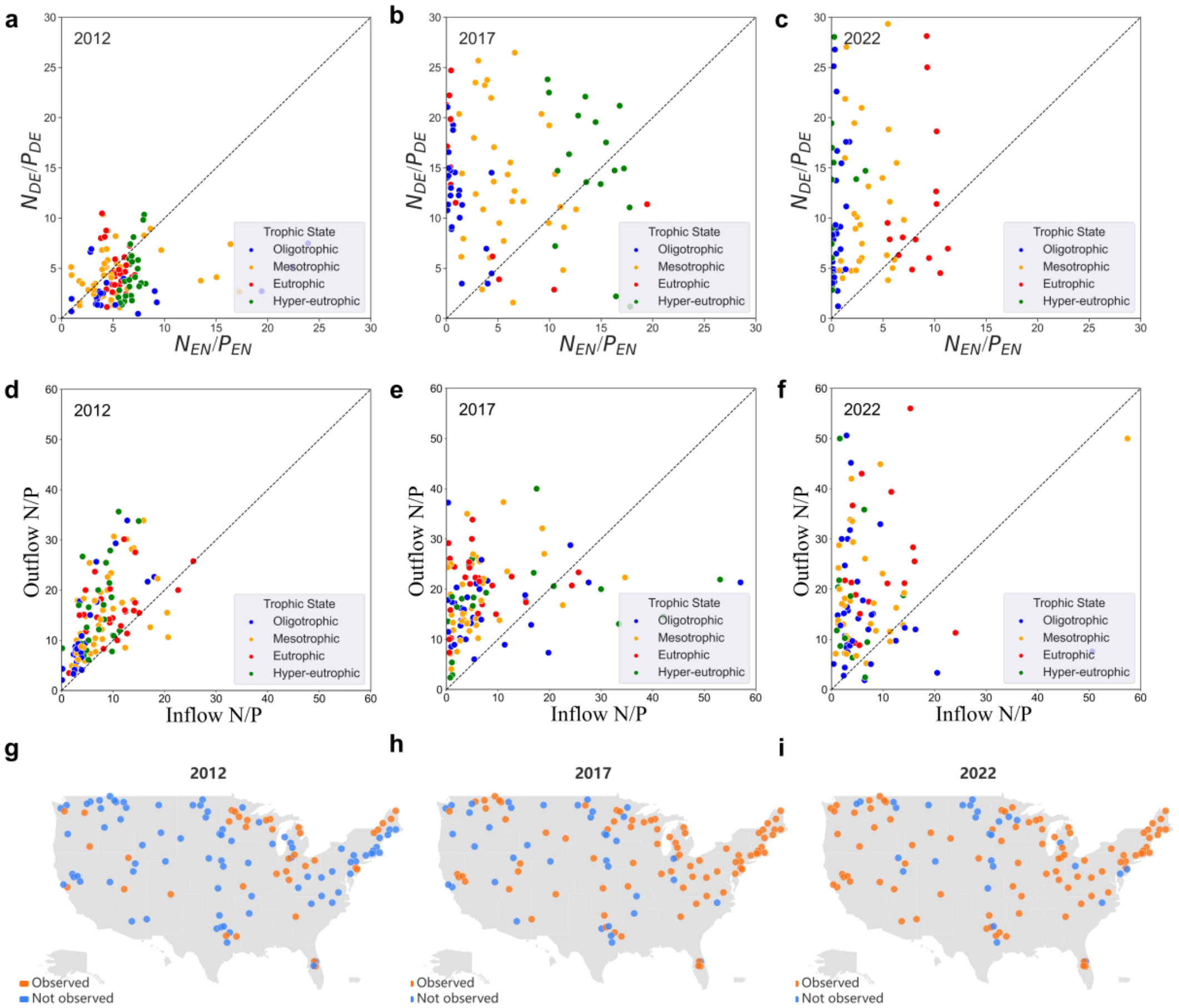

Fig. 5 Stoichiometry of nutrient cycling in 2012 and 2017. The N/P ratios of in-lake enrichment and depletion (a) in 2012, (b) 2017, and (c) 2022. These panels replicate the preferential retention pattern shown in Fig. 2a-c, and the corresponding statistical tests yielded identical results. The outflow N/P ratios exceeded inflow ratios (d) in 2012 ($t(120) = 17.77$, $p < 0.001$, Cohen's $d = 1.62$, 95% CI [0.76, 0.95]), (e) 2017 ($t(120) = 9.04$, $p < 0.001$, Cohen's $d = 0.82$, 95% CI [0.50, 0.78]) and (f) 2022 ($t(120) = 9.87$, $p < 0.001$, Cohen's $d = 0.90$, 95% CI [0.54, 0.80]). The equations 11 − 14 in the Methods section are the details for inflow and outflow derivation. The proportions of lakes in NMD-like paradoxical condition (g) count for 38% in 2012, (h) 69% in 2017, and (i) 76% in 2022. Source data are provided as a Source Data file.

27. Line 223 suggested edit: US lakes have been at the forefront …century, demonstrating a slow, yet steady, growth of eutrophication from 2012-2022
Line 225 Yet, there has been no significant
Line 226 remove s on literature
Line 228 remove It could be that…, and start sentence with Over
Line 233 weakens not weakened

【Response】Thanks for your edits. The contents with your edits have been revised as shown below. *Please see lines 229-241 for revisions in the text.*

U.S. lakes have been at the forefront of the new wave of eutrophication since 21st century, demonstrating a slow, yet steady, growth of eutrophication trend during 2012-2022 (Supplementary Text 9 & Supplementary Figs. S16 - S18). Yet, there has been no significant change in external loading of U.S. lakes (Supplementary Text 4). Based on the integrated analysis of this study and the existing literature, we propose a conceptual framework to systematically explain the nutrient evolution of U.S. lakes in the new wave of eutrophication since the early 21st century. Over the past two decades, the dominance of internal cycling has led to sustained net retention, reigniting eutrophication, indicated by the fact that the number of lakes in the USA with net retention of N and P continued to increase until 2017, and the net nutrient retention was primarily driven by internal cycling (Fig. 6). After the new wave of eutrophication reaches a certain stage, the inhibitory effect of nutrient on its net retention has exceeded the promotive effect from internal cycling, since the trend of net nutrient retention weakens with the progression of eutrophication. Most lakes have reached their net retention capacity, evidenced by the net retention of N and P began to decline during 2017-2022.

28. Line 236 insert trend after eutrophication

【Response】Thanks for your edit. A word “trend” has been inserted after the word “eutrophication”. *Please see lines 242-243 for revisions in the text.*

The seemingly slowly-increasing eutrophication trend clearly indicates that the lakes are facing a regime shift.

29. Line 244 Text- especially in colored box in Figure 6 too small to read

【Response】Thanks for your suggestion. The text in the box in Fig. 6 enlarged as suggested. *Please see the revised Fig. 6 for revisions.*

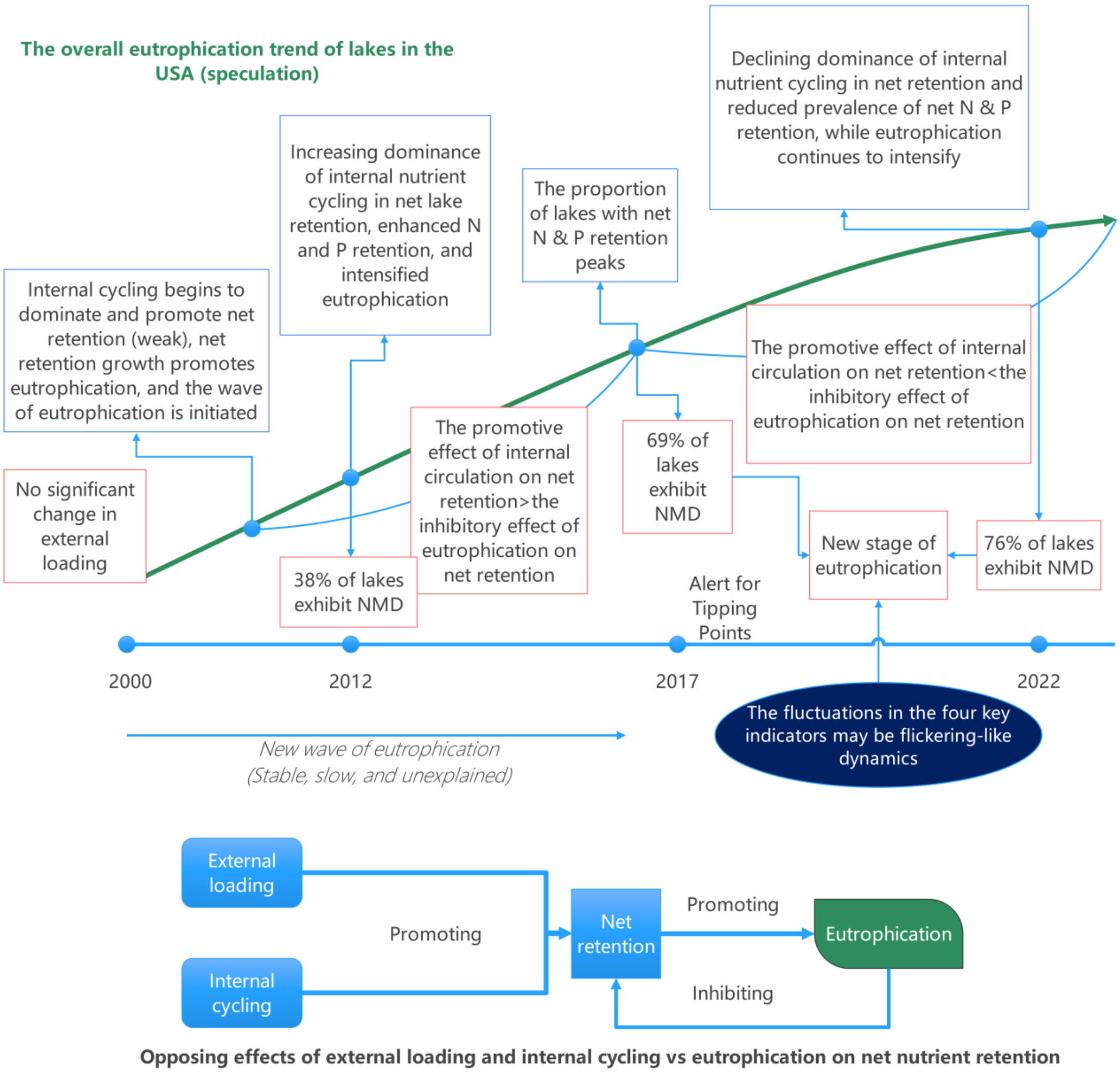

Fig. 6 The potential overall eutrophication trajectory of lakes in the USA. This conceptual diagram presents a hypothesized eutrophication trajectory of lakes in the USA based on the findings of this study. The lower section is a schematic representation included to facilitate understanding of the opposing influences of external loading and internal cycling, and eutrophication, on net nutrient retention.

30. Line 313 insert has before initiated

【Response】Thanks for your edit. A word "has" has been inserted before the word "initiated". *Please see lines 321-322 for revisions in the text.*

It could be that an increase in net retention of N and P in lakes has initiated the new wave of eutrophication.

<u>**Reviewer #2**</u>:

Review of "Eutrophication Dynamics: The Shift of Preferential Nutrient Retention and the Era of Nutrient Metabolic Disorder" by Zhou et al.

1. Line 83: It is also possible that the imposed P controls themselves shifted the N:P stoichiometry, which then pushed the system towards this regime shift (although it is questionable, and links directly to the points I mentioned in my comments below). In that case, both N and P controls would have been important at the same time.

【Response】We thank the reviewer for this constructive comment. We agree that both nitrogen and phosphorus controls can be important simultaneously, which is also one of the key conclusions of this study. However, regarding the specific possibility raised by the reviewer that "the imposed P controls themselves shifted the N:P stoichiometry," this hypothesis is not supported by our additional analyses.

Specifically, we examined temporal changes in the total nitrogen to total phosphorus (TN/TP) ratio for 121 lakes during the period 2012–2022. The results indicate that the longstanding P control measures implemented by U.S. did not lead to a significant change in the overall N:P stoichiometry over the study period (ANOVA, F = 0.8150, P = 0.4434). This suggests that the observed ecosystem regime shift cannot be simply attributed to a stoichiometric imbalance induced by P reduction.

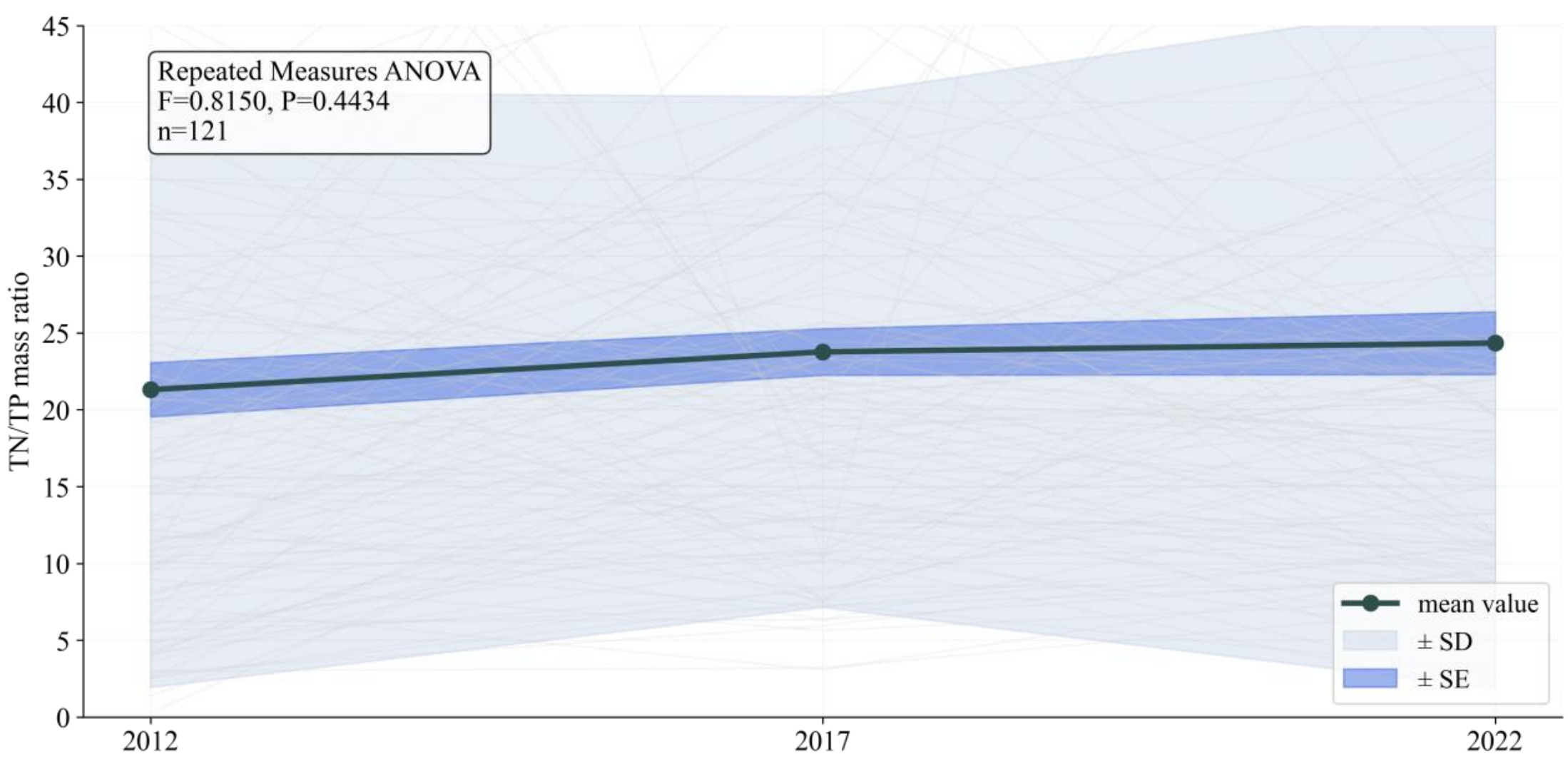

Figure S19 Temporal changes in the TN/TP ratio across 121 U.S. lakes from 2012 to 2022. Thin lines represent individual lakes, while solid circles indicate annual mean values with ±SE (dark shading) and ±SD (light shading). A repeated-measures ANOVA detected no significant temporal change in the TN/TP ratio over the study period ($F = 0.8150$, $P = 0.4434$, $n = 121$).

These findings support our decision to focus on nutrient retention rather than relying solely on the N:P ratio. Retention provides more direct insight into internal nutrient processing and lake-specific nutrient dynamics, which are not fully captured by bulk stoichiometric indicators. Therefore, while both N and P controls are important, our results indicate that nutrient retention is a more appropriate framework for understanding the mechanisms underlying the observed system response.

We have added additional discussion at in the section addressing regime shifts, to strengthen the evidence supporting our interpretation that internal nutrient dynamics push the system toward regime shift.

Please see lines 243-245 for revisions in the text.

This interpretation is supported by the repeated switching of four key indicators, indicative of flickering-like dynamics and the emergence of an associated NMD-like state, rather than by a P-control-induced shift in the TN/TP ratio (Supplementary Fig. S19).

2. Lines 111 to 119: It looks like the authors intended to refer to Figure 2d, because Figure 2a shows log-scaled enrichment and retention ratios for N and P, which are not the same as percentage changes in preferential retention. None of the panels in 2a, 2b or 2c actually present the percentage increase or decrease that the text is describing.

【Response】We appreciate your careful reading. We acknowledge that the wording in Lines 111 – 119 may not have been sufficiently clear, which may have also contributed to the confusion noted in the third comment. In fact, one lake shows preferential N retention ($P_{EN}/P_{DE} > N_{EN}/N_{DE}$) ,which is reflected in Fig. 2a - 2c by the data point lying above the dashed y = x line. One lake shows preferential N retention ($P_{EN}/P_{DE} < N_{EN}/N_{DE}$) ,which is reflected in Fig. 2a - 2c by the data point lying below the dashed y = x line.

Lines 112 to 117: This whole section needs to be rewritten with clearer statements and the correct figure references. The figure numbers do not match the text, and the caption itself is confusing. For example, it says "Lakes located beyond the black line show a pattern of preferential N retention", but there are two different black lines in the plot, the dotted 1:1 line and the solid axes. "Beyond" is ambiguous. The authors should explicitly state which region or quadrant they mean. In the same place, the authors also need to clearly explain how the enrichment-to-depletion ratio relates to nutrient retention and why it is inversely proportional, following Wu et al. Without this clarification, the reader will simply be confused.

【Response】We thank the reviewer for this constructive comment. We have fully revised the entire section as suggested. Specifically, we rewrote the text to improve clarity and precision, corrected and harmonized all figure references, and revised the figure caption to eliminate ambiguity. In addition, as requested by the reviewer, we have expanded the explanation of how the enrichment-to-depletion ratio relates to net nutrient retention and clarified the basis for its inverse relationship, placing this explanation immediately after the definitions of enrichment and depletion in the figure caption.

Please see lines 111-120 for revisions in the text.

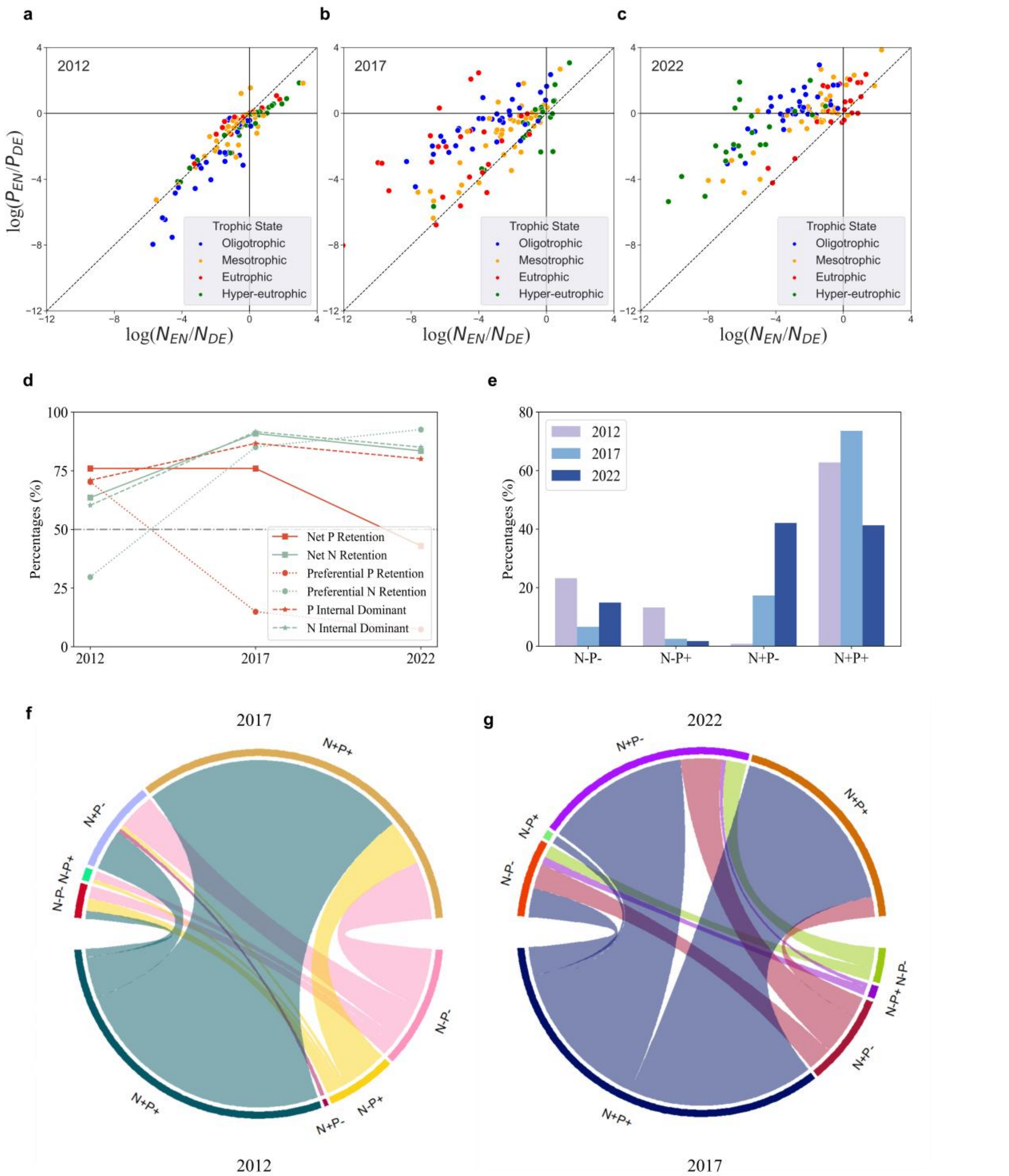

Fig. 2 Pattern of nutrient retention. (**a**) Pattern of net nutrient retention in 2012; and (**b**) in 2017; and (**c**) in 2022. P_{EN} and N_{EN} mean in-lake enrichment of P and N, respectively. P_{DE} and N_{DE} mean in-lake depletion of P and N, respectively. Lakes retain nutrients through in-lake depletion processes, while in-lake enrichment supplies nutrients in excess of those depleted, lakes can no longer be considered net nutrient sinks. Accordingly, $\log(N_{EN}/N_{DE}) < 0$ and $\log(N_{EN}/N_{DE}) \geq 0$ are defined as positive and negative net retention of N, respectively, and the same criteria are applicable for P[3]. Furthermore, preferential N retention is defined[3] as $P_{EN}/P_{DE} > N_{EN}/N_{DE}$, while preferential P retention is defined as $P_{EN}/P_{DE} < N_{EN}/N_{DE}$. Lakes located above (below) the black dashed y = x line show a pattern of preferential N (P) retention. (**d**) Changes in the proportion of different types of lakes from 2012 to 2022. (**e**) Percentages of four types of lakes in 2012 and 2017. N+ means positive net N retention while N- means negative net N retention. P+ means positive net P retention while P- means negative net P retention. (**f**) Conversion of four types of net nutrient retention from 2012 to 2017; and (**g**) from 2017 to 2022.

Among the studied lakes, most exhibited preferential P retention in 2012 (70.3% below the dashed line; Fig. 2a), consistent with previous findings[3]. From 2012 to 2022, this pattern progressively shifted toward preferential N retention. The proportion of lakes exhibiting preferential N retention increased to 85.1% in 2017 and further to 92.6% in 2022 (above the dashed line; Fig. 2b & 2c), accompanied by a sharp decline in preferential P retention from 14.9% in 2017 to 7.4% in 2022 (below the dashed line; Fig. 2b & 2c). Collectively, these results reveal a major shift in preferential nutrient retention across U.S. lakes between 2012 and 2022. This transition toward preferential N retention, largely unanticipated under historically P-dominated conditions, suggests that lakes experiencing ongoing eutrophication can undergo a substantive reorientation in nutrient retention, shifting from a long-standing preference for P toward increasing N retention.

3. Line 146: The claim that "the preference of N retention has suppressed the net P retention in most lakes" is not supported by the evidence. Even if N^+P^- lakes increased, that does not show that N retention caused a decline in P retention, nor does it demonstrate a suppression mechanism. The manuscript is inferring causality from a pattern, and the logic is pushed too far beyond what the data actually show. Statement related to Figure 3: The decline in net retention with increasing trophic level is a well-known feature of eutrophic systems. It simply means that more eutrophic lakes retain nutrients less effectively. This does not establish the causal link that the authors are implying, that preferential N retention is suppressing P retention. Needs explanation and justification.

【Response】 Thank you for this important comment. We agree that descriptive patterns alone do not justify causal inference. To address this concern, we conducted an additional analysis using a two-way fixed-effects Logit model to examine whether the observed pattern is consistent with a potential causal relationship between preferential N retention and net P retention.

After controlling for unobserved time-invariant heterogeneity across lakes and common temporal shocks, the model indicates a statistically significant negative association between preferential N retention and net P retention ($\beta_1 = -2.54$, $p < 0.001$), which is consistent with an inhibitory relationship. Standard multicollinearity diagnostics suggest no severe collinearity issues. The results remain robust when using a Probit specification, alternative subsamples, and placebo tests.

We note that the parallel trends assumption is only partially supported, which may imply that the magnitude of the estimated effect could be overstated. We therefore interpret the results cautiously and revise the manuscript to frame the findings as

providing supportive evidence rather than definitive proof of a causal suppression mechanism. Corresponding revisions have been made in main text and Supplementary Text 3 to clarify the scope and limitations of the inference.

Please see lines 151-154 for revisions in the main text.

By tracking the nutrient dynamics of 121 lakes in the USA, we find that accompanying the shift in dominance of the preferential nutrient retention from P to N, the number of the N+P- group lakes significantly increased while that of the N-P+ group lakes significantly decreased (Fig. 2e, 2f & 2g). This pattern is consistent with an inhibitory effect of preferential N retention on net P retention. To further test this interpretation, we conducted a causal inference analysis using a two-way fixed-effects Logit model, which provides additional support for a negative effect of preferential N retention on net P retention (Supplementary Text 3 & Supplementary Table 1).

Please see lines 267-300 for revisions in the SI.

Supplementary Text 3: Causal inference analysis of the relationship between preferential N retention and net P retention

To further examine whether the observed association between preferential N retention and net P retention is robust to a causal inference framework, we employed a fixed-effects Logit model with lake fixed effects and a post-period time fixed effect (equations 5 & 6). This approach allows us to control for unobserved, time-invariant heterogeneity across lakes (e.g., geomorphology, baseline nutrient cycling characteristics) as well as common temporal shocks affecting all lakes (e.g., regional climate variability or broad management changes). By accounting for both lake-specific and time-specific effects, the model reduces potential confounding arising from stable lake attributes and shared temporal trends, thereby providing a more rigorous assessment of the relationship between preferential N retention and net P retention.

$$\log\left(\frac{P_{i,t}}{1-P_{i,t}}\right)=\alpha_i+\gamma_t+\beta_1 \square X_{centered,i,t}+\beta_2 \square N_{centered,i,t}+\varepsilon_{i,t} \quad (5)$$

$$\gamma_t=\beta_0+\beta_3 \square year_{2017-2022,t} \quad (6)$$

Symbols	Explanations
t	Time index, taking values 2012, 2017, and 2022.
$P_{i,t}$	Binary indicator of net phosphorus (P) retention status for lake i in year t, where 1 denotes positive and 0 denotes negative.
$\log(\frac{P_{i,t}}{1-P_{i,t}})$	Log-odds of net P retention, representing the Logit transformation that maps the binary outcome probability to a continuous scale.

Symbols	Explanations
t	Time index, taking values 2012, 2017, and 2022.
α_i	Lake-specific fixed effect capturing time-invariant characteristics of lake i
γ_t	Common time trend term accounting for shared temporal influences across lakes, approximated by a post-2017 time indicator to control for broad changes affecting the 2017–2022 period
$X_{centered,i,t}$	Within-lake centered indicator of preferential nitrogen (N) retention for lake i in year t, where 1 indicates preferential N retention and 0 indicates preferential P retention.
$N_{centered,i,t}$	Within-lake centered indicator of net nitrogen (N) retention status for lake i in year t, where 1 denotes positive and 0 denotes negative
$year_{2017\_2022,t}$	Time dummy variable, equal to 1 for observations in 2017 and 2022, and 0 for observations in 2012.
β_0	Intercept term
β_1	Coefficient of primary interest, capturing the association between preferential N retention and the likelihood of net P retention
β_2	Coefficient associated with net N retention status, included as a control variable
β_3	Coefficient capturing the common post-2017 time trend.
$\varepsilon_{i,t}$	Unobserved error component implicitly accounted for in the maximum-likelihood estimation of the Logit model

The fixed-effects Logit analysis provides consistent evidence of a negative association between preferential N retention and net P retention. The estimated coefficient on preferential N retention is −2.54 (SE = 0.41), which is highly statistically significant ($z = -6.21$, $p < 0.001$), with the 95% confidence interval [−3.35, −1.74] excluding zero.

Model diagnostics indicate satisfactory fit and validity, with a McFadden pseudo R^2 exceeding conventional thresholds and no evidence of problematic multicollinearity. Although heteroskedasticity is detected, inference is robust to cluster-robust standard errors. Importantly, the negative effect of preferential N retention remains stable across alternative specifications, including Probit estimation and subsample analyses, and is further supported by placebo tests showing that the estimated effect lies far outside the distribution of coefficients obtained under randomized treatment assignments.

While the parallel trends assumption is not fully satisfied and thus warrants cautious interpretation, the convergence of results across multiple robustness and validity checks supports the conclusion that preferential N retention exerts an inhibitory effect on the probability of net P retention.

Table S1 Model diagnostics and robustness checks for the two-way fixed-effects Logit analysis

Category	Statistic	Value	Interpretation
Model fit	McFadden pseudo R^2	0.259	Indicates good model fit (>0.2)
	Log-likelihood	−100.89	—
	LR statistic	70.67	Model jointly significant
	LR test p-value	<0.001	Reject null of no explanatory power
Predictive ability	AUC	0.815	Strong discrimination ability
Heteroskedasticity	Test result	Significant	Heteroskedasticity detected; cluster-robust SE applied
Overdispersion	Dispersion parameter	1.017	No overdispersion (≈1)
Multicollinearity	VIF (X_centered)	2.46	No multicollinearity (<5)
	VIF (N_centered)	1.21	No multicollinearity (<5)
	VIF (year_2017_2022)	2.55	No multicollinearity (<5)
	VIF (constant)	6.09	Mild multicollinearity (acceptable)
Core result (Logit)	Coefficient on preferential N retention	−2.54	Negative and highly significant
	Standard error	0.41	High estimation precision
	z-value	−6.21	Significant at 1% level
	95% CI	[−3.35, −1.74]	Excludes zero
Robustness (Probit)	Coefficient on preferential N retention	−1.54	Same sign as Logit
	p-value	<0.001	Statistically significant
Subsample analysis	Coefficient on preferential N retention	−6.68	Same direction
	p-value	<0.001	Significant
Consistency check	Sign consistency	Yes	Across Logit, Probit, and subsamples
	Significance consistency	Yes (core variable)	Stable inference
Parallel trends	Test conclusion	Not fully satisfied	Results interpreted cautiously
Placebo test	Mean placebo coefficient	−0.01	Centered near zero
	Placebo SD	0.40	—
	True coefficient z-score	−6.32	Strong deviation from placebo
	False significance rate	4.7%	Low
	Placebo conclusion	Robust	Core result unlikely driven by chance

4. Line 163–170: The authors use the Wu et al. algorithm to calculate INCI, but Fig. 3 only shows percentages of lakes based on INCI categories. In Supplementary Text 4, they only show external loading and inflow statistics for N and P. I would like to actually see the INCI values themselves. These should be in the supplementary material so the reader gets a clearer picture of what the model is producing.

【Response】 Thank you for this helpful suggestion. We agree that presenting only the categorical distribution of INCI values in Fig. 3 does not fully convey the model outputs. We have now added the actual INCI values to the Supplementary Fig. 15. This figure directly presents the INCI values produced by the Wu et al. algorithm, addressing the reviewer' s request for explicit model outputs. Specifically, a new supplementary data (INCI.csv) has been included in *Data Availability*, which reports the calculated INCI values for each lake. We believe these additions provide a clearer and more transparent presentation of the model outputs.

Please see lines 173-175 for revisions in the text.

We find that, in the three surveyed years (2012, 2017, and 2022), the net retention of both N and P in the lakes in the USA was primarily driven by internal cycling rather than external loading (Fig. 3 red dashed line and green dashed line, & Supplementary Fig. S12 - S15)

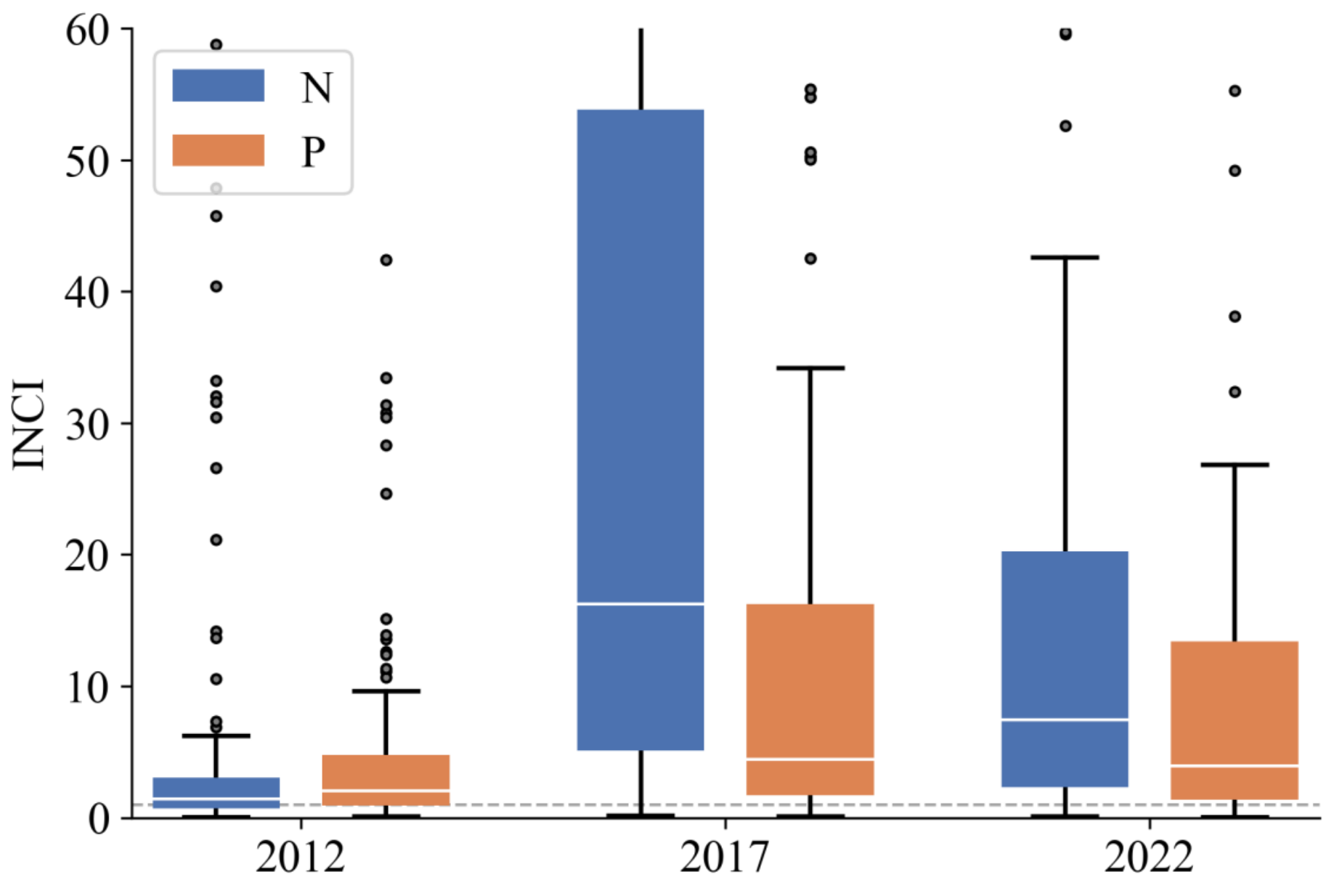

Supplementary Fig. 15 **Distribution of N_{INCI} and P_{INCI} values across 121 U.S. lakes**. Box-and-whisker plots showing the distributions of nitrogen (N, blue) and phosphorus (P, orange) - related INCI values across 121 U.S. lakes. The boxes indicate the interquartile range (25th - 75th percentiles), the horizontal lines within the boxes represent median values, whiskers extend to 1.5 times the interquartile range, and individual points denote outliers. The grey dashed horizontal line at y = 1 represents

the INCI classification threshold (internal dominant model: INCI > 1; external dominant model : INCI <1).

Please see lines 459-462 for revisions in the text.

Data availability

All data that support the findings in the current study are available within the Article and its Supplementary Information. Source data including USL dataset, the f_{NANI} & f_{NAPI} data, and INCI data are available at Figshare (https://figshare.com/s/481c9bef67a527de5ae8).

5. Line 175–176: I am not sure how the authors arrive at "This consistently suggests that the influence of internal cycling on net nutrient retention also has an upper limit". What is this upper limit? The sentence needs more explanation before making that conclusion.

【Response】 We thank you for this comment and agree that the previous wording required clearer explanation. We have therefore revised this part of the manuscript to avoid the use of the term "upper limit" and instead describe the observed patterns more directly. To avoid potential misunderstanding, we clarify our interpretation as follows.

Specifically, in oligotrophic lakes, the proportion of systems in which net nutrient retention is predominantly regulated by internal cycling increases with progressing eutrophication (Fig. 3a red dash line (P) and green dash line (N)), which is consistent with conclusions from recent studies. In contrast, in mesotrophic and more eutrophic lakes, this proportion exhibits a non-monotonic pattern (Fig. 3b-d red dash line (P) and green dash line (N)), increasing initially but subsequently declining.

Considering that overall eutrophication of U.S. lakes has gradually intensified from 2012 to 2022, we infer that, during the early stages of eutrophication, internal cycling becomes increasingly influential in controlling net nutrient retention, leading to a growing prevalence of internally regulated systems, as previously reported. However, once eutrophication progresses beyond this early stage, the influence of internal cycling on net nutrient retention does not continue to increase indefinitely, the role of external nutrient loading becomes increasingly important again, resulting in a decline in the proportion of lakes in which net nutrient retention is dominated by internal cycling (that is why we used "upper limit"). The manuscript has been revised accordingly to clarify this stage-dependent interpretation.

Please see lines 178-182 for revisions in the text.

The number of lakes at a given trophic level in which their net N and P retention was predominantly regulated by internal cycling generally showed an initial increase followed by a decrease from 2012 to 2022 (Fig. 3). Furthermore, while the proportion

of lakes in which net N and P retention was predominantly regulated by internal cycling increased with trophic level in oligotrophic systems, this relationship became non-monotonic in mesotrophic and more eutrophic lakes, increasing initially but declining at higher trophic levels (Fig. 3). This non-monotonic response may suggest a weakening of internal cycling dominance at advanced stages of eutrophication.

6. Supplementary Text 5, Line 371 – 372: To rule out interannual variability, the authors need to present a proper continuous time series for at least a decade or more.

Supplementary Text 5, Line 364 – 365: The statement that "USEPA reports show 10-year nutrient input data remain largely stable" does not rule out extreme events or short-term spikes, which could be important. This again points to the need for a long-term dataset.

【Response】 We agree with the reviewer that, given the limited temporal resolution of our dataset, interannual variability cannot be formally ruled out in a strict statistical sense. We have therefore revised the manuscript to avoid the wording "rule out" and now state that interannual variability alone is "unlikely to fully explain" the observed patterns.

We also clarify in the Supplementary Information that our analysis is intended as a comparison of alternative mechanisms based on a weight-of-evidence approach, rather than as a definitive diagnosis. The relevant sections in *Supplementary Text 5* have been substantially revised to reflect this framing (now it is *Supplementary Text 6: Evaluating alternative explanations for indicator reversals aside from flickering-like dynamcis*).

To support this revised interpretation, we provide several complementary lines of evidence in *Supplementary Text 6*, indicating that simple interannual variability is an insufficient explanation for the repeated reversals observed in a subset of lakes.

Please see lines 397 – 468 in the Supplementary Information for details of these revisions.

Supplementary Text 6: Evaluating alternative explanations for indicator reversals aside from flickering-like dynamcis

Diagnosing flickering in a strict dynamical-systems sense requires long, continuous, and high-frequency time series, which are not available for most U.S. lakes. Accordingly, the purpose of this section is not to formally confirm flickering, but to evaluate whether alternative explanations—particularly externally driven interannual variability—are sufficient to account for the repeated reversals observed in a small subset of lakes. Based on the multiple lines of evidence summarized

below, we argue that simple interannual variability alone is unlikely to fully explain the observed patterns.

-Interannual variability:

Interannual variability in nutrient inputs and concentrations has been proposed as a potential explanation for apparent reversals in nutrient retention behavior, especially in the context of long-term phosphorus control efforts in the United States. While such variability undoubtedly exists, several considerations suggest that it is insufficient, on its own, to explain the repeated switching observed in our categorical retention indicators.

First, long-term national and regional assessments indicate that mean nutrient concentrations and external nutrient loads in U.S. lakes have remained broadly stable over the past two decades, with no evidence of coherent interannual oscillations or systematic directional shifts at the continental scale. In the absence of large or coordinated external perturbations, it therefore appears unlikely that interannual variability alone would repeatedly drive binary retention indicators across sign thresholds.

Second, this study deliberately employs categorical (binary) indicators of nutrient rather than raw numerical estimates. While this approach intentionally sacrifices some quantitative resolution, it substantially reduces uncertainty by limiting sensitivity to measurement noise and parameter variability. Our uncertainty and sensitivity analyses demonstrate that moderate perturbations in input variables do not readily induce shifts in indicator classification, making reversals even less likely. Because a reversal requires two consecutive shifts, the probability of such back-and-forth switching is further reduced. Consequently, typical year-to-year fluctuations, even if present, are unlikely to propagate into repeated reversals of categorical retention states.

Taken together, these two lines of evidence indicate that pronounced interannual variability is not apparent in the available data, and that moderate fluctuations, where they occur, are unlikely to translate into repeated changes in binary retention indicators. On this basis, interannual variability alone appears insufficient to explain the observed patterns.

Third, we examined the occurrence of extreme climatic or hydrological events during the three sampling years and found no indication that the lakes exhibiting repeated reversals experienced major disturbances coincident with the observation periods. This further reduces the plausibility that short-term spikes or episodic external forcing underlie the observed switching behavior.

Fourth, this fails to interpret the emergence of a nutrient metabolic disorder-like state. Logically, if interannual variability of N and P data were solely responsible, one would expect concurrent occurrences of nutrient retention reversal and shift in N/P mass ratios in nutrient inflows and outflows, which is inconsistent with the phenomenon derived from the datasets. The nutrient

metabolic disorder-like state suggests much more complex interactions of the factors beyond mere annual fluctuations.

Finally, we acknowledge that interannual variability and noise-induced transitions can produce superficially similar observational patterns, particularly when systems operate near critical thresholds. Distinguishing between these mechanisms would require long, high-frequency time series, which are not available in the present study. Nevertheless, when considered alongside multiple lines of evidence for enhanced internal nutrient cycling intensity (INCI), and our finding that net retention of both nitrogen and phosphorus in U.S. lakes is primarily governed by internal cycling rather than external loading, the observed behavior appears more consistent with internally mediated, noise-amplified dynamics than with externally forced interannual variability.

7. If they want to justify flickering or tipping-point behaviour, they need to show high-frequency switching, noise-driven exits and returns to the attractor. None of this can be shown with only three snapshots. Three years of data are not enough to call it flickering.

【Response】We fully agree with the reviewer that a rigorous demonstration of flickering or tipping-point dynamics requires long, high-frequency time series capable of resolving rapid switching, noise-driven departures from, and returns to, attractors. With only three temporally sparse observations, such dynamics cannot be conclusively demonstrated, and we acknowledge that the available data are insufficient to formally diagnose flickering in the strict dynamical-systems sense.

However, comprehensive, long-term, high-frequency nutrient datasets for a large number of U.S. lakes remain extremely scarce. Within this data-limited context, and based on the multiple independent lines of evidence summarized in *Supplementary Text 7: The reasons that ascribe the reversals to flickering-like dynamics*, we therefore present flickering not as a definitive diagnosis, but as a plausible and forward-looking hypothesis.

We have revised the manuscript and SI accordingly to weaken deterministic language and to replace *flickering* with *flickering-like dynamics* throughout the text and SI. This revision is intended to clearly distinguish between a formal diagnosis of flickering, which would require long and high-frequency time series, and the use of flickering as a conceptual framework to interpret the observed instability in nutrient retention states (flickering-like dynamics). We hope that this more cautious formulation appropriately reflects the limitations of the available data and the exploratory nature of the hypothesis, while still emphasizing the relevance of flickering-like behavior as an important and understudied possibility in large-scale lake nutrient dynamics.

8. Supplementary Text 5, Line 398 – 400: Again, I cannot see the actual INCI results anywhere in the manuscript.

【Response】 Thank you again for this helpful suggestion which you have raised in *Question **4***. We have now added the actual INCI values to the Supplementary Fig.15. Specifically, a new supplementary data (INCI.csv) has been included in *Data Availability*, which reports the calculated INCI values for each lake. We have also added a reference to this new supplementary text in *Lines 173 -175* of the revised manuscript to guide readers to the detailed INCI results.

*Please see details in Answer **4**.*

9. Figure 4: Needs improvement. The points and colours are barely visible.
【Response】 Thanks for your revision suggestion and edit.

Please see the revised Fig. 4 for revisions.

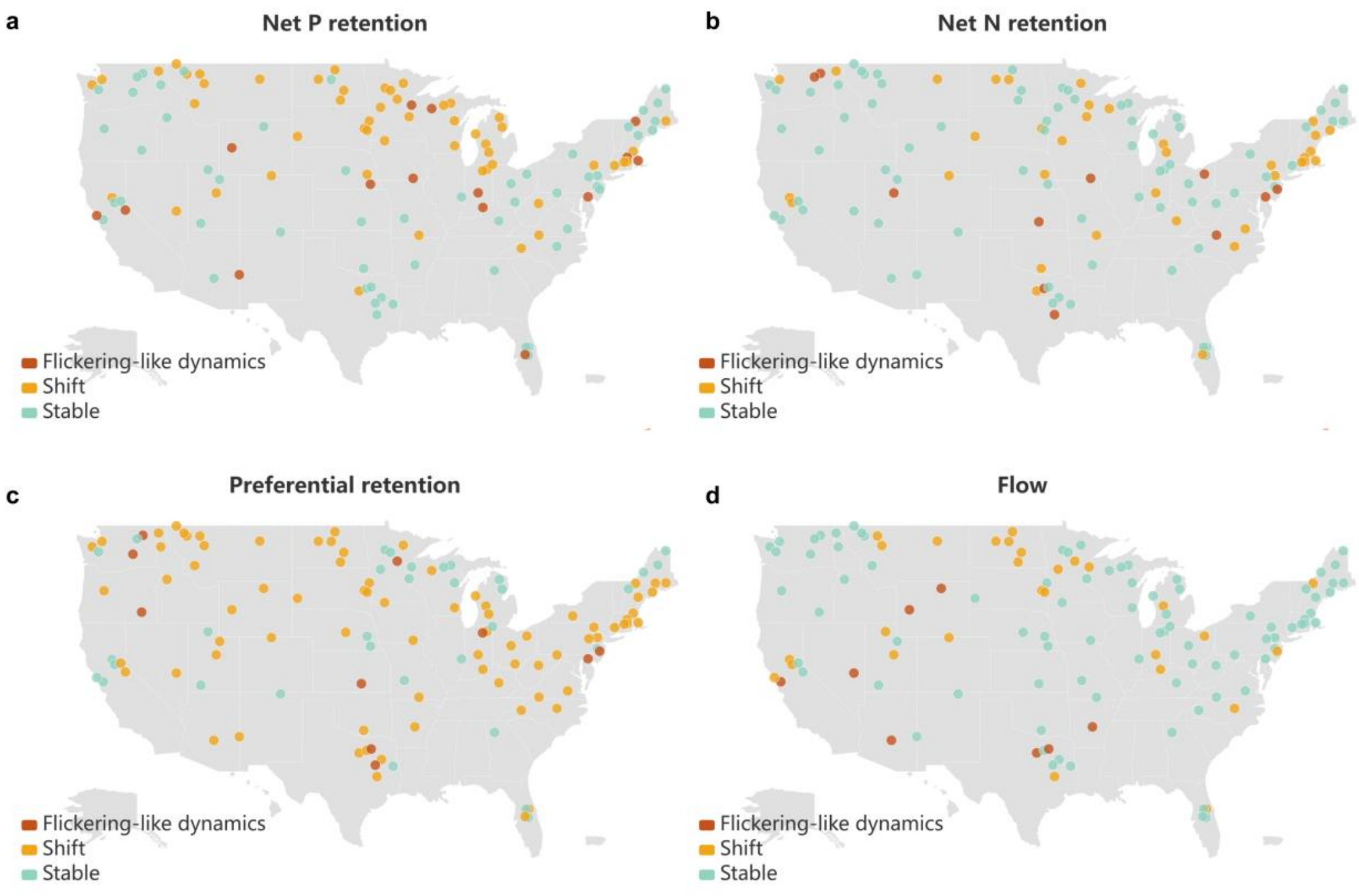

Fig. 4 Spatial distribution of the flickering-like dynamics of key metrics. The types can be categorized based on the number of shifts as: 2 shifts (flickering-like dynamics), 1 shift (shift), and no shift (stable). Four graphs show flickering-like pattern of (a) net P retention switching between positive and negative, (b) net N retention switching between positive and negative, (c) preferential nutrient retention switching between N and P, (d) flow N/P switching between inflow N/P over outflow N/P and outflow N/P over inflow N/P. Source data are provided as a Source Data file.

10. Line 200–215: Fig. 5a shows P retention higher than N in 2012, which matches the model's outflow N/P being above the 1:1 line, meaning more N is released than P. But in 2017 and 2022 (Fig. 5b and 5c), when NDE/PDE is higher (points above the 1:1 line), meaning N should be retained more than P, I would expect to see the same trend in the inflow–outflow plots. Instead, the outflow plots still show more N above the 1:1 line, meaning more N release than P, which is the opposite. I am not convinced how the authors conclude this is "anomalous". The model and the observations are not aligned. And if the authors think this reflects ongoing eutrophication, they should show evidence for that.

【Response】 Thanks for your revision suggestion. We agree that, under canonical nutrient cycling assumptions, preferential nitrogen retention would be expected to coincide with proportionally reduced nitrogen export, and that an apparent inconsistency between retention and export therefore warrants careful interpretation. Our intention in highlighting this pattern was not to imply a model inconsistency, but rather to emphasize a system-wide decoupling between nutrient retention and net export that emerges coherently across the majority of the 121 studied lakes after 2017.

Importantly, in 2012 the model reproduces nutrient retention and export relationships that are fully consistent with theoretical expectations and previous studies, with preferential phosphorus retention accompanied by elevated outflow N/P ratios. This agreement provides confidence that the model captures canonical nutrient processing under non-anomalous conditions. In addition, data completeness and model robustness are described in detail in the Methods section. Together, these considerations suggest that the observed post-2017 pattern is unlikely to arise from data limitations or model artefacts.

Furthermore, we explicitly link this decoupling to flickering-like dynamics identified in the preceding analysis, which theory predicts to occur near critical transitions in ecosystem functioning. Consistent with these expectations, the post-2017 pattern is now described as exhibiting anomalous characteristics consistent with a nutrient metabolic disorder–like (NMD-like) regime, rather than being asserted as a definitively established anomalous state.

Specifically, we have revised the relevant text to emphasize:

(i) the agreement between model results and established theory in 2012;

(ii) the system-wide and temporally coherent emergence of the paradoxical retention–export pattern after 2017;

(iii) the removal of speculative language attributing this pattern directly to ongoing eutrophication;

(iv) the interpretation of this pattern as a regime-level transition consistent with, rather than definitive proof of, an NMD-like state.

We hope these revisions address the reviewer's concern and clarify why we interpret the observed retention–export paradox as a meaningful signal of altered nutrient processing, rather than an internal inconsistency or artefact of the modeling framework.

Please see lines 197-198, lines 206-221 for revisions in the text.

The occurrence of flickering-like dynamics indicates that a critical point in nutrient cycling is approaching [22], at which qualitative changes in nutrient processing are theoretically expected.

Consistent with the theoretical expectation associated with flickering dynamics, our results further reveal that between 2012 and 2017 the nutrient dynamics of lakes across the USA underwent a pronounced and system-wide shift, exhibiting anomalous characteristics consistent with a nutrient metabolic disorder-like (NMD-like) pattern (Fig. 5g - 5i). In 2012, P was more preferentially retained than N (Fig. 5a), and the model-derived outflow N/P ratios exceeded inflow ratios (Fig. 5d), consistent with theoretical expectations and previous studies3. However, most lakes exhibit preferential retention of N after 2017 (Fig. 5b & 5c), while paradoxically maintaining substantially elevated outflow N/P ratios relative to inflow (Fig. 5e & 5f). The emergence of this counterintuitive and widespread pattern across the majority of lakes suggests a potential transition to a distinct nutrient processing regime. The defining features of this regime conceptually resemble metabolic disorder observed in humans (Supplementary Text 8, Supplementary Table S2). Previous studies have primarily focused on changes in absolute nutrient quantities, which may not fully capture imbalances between N and P. Such imbalances may therefore have been less readily detected.

11. Line 248 - 249: I understand Extra Trees performs better than the other ML methods for f_NAPI, but with R^2 = 0.53 and MSE = 6.92, it is not strong enough to claim the final model has "lower uncertainty". This is also evident in figure S18(b) with overestimation of f_NAPI at lower actual value. The unexplained variance is high and feeding this into the Bayesian model risks introducing systematic bias, since the ML-based external loads (f_NAPI) are not well constrained.

【Response】We appreciate the reviewer's concern regarding the moderate predictive performance and potential overestimation of f_{NAPI}. On the test dataset, f_{NAPI} predictions show a mean positive bias of 0.47, with 54.17% of samples being overestimated and 45.83% underestimated, indicating a modest overall tendency toward overprediction.

Importantly, this potential bias was explicitly considered in our modeling framework. As detailed in Supplementary Text 10: Uncertainty and sensitivity, the Bayesian hierarchical model substantially reduces the propagation of uncertainty from external nutrient load estimates into the final inference. In this framework, f_{NAPI} acts as an upstream forcing variable whose uncertainty is partially absorbed at hierarchical levels, rather than directly determining model outcomes.

To more directly address this concern, and to provide a concrete example of the robustness of our main conclusions, we conducted a conservative sensitivity analysis by uniformly reducing f_{NAPI} values in 2017 and 2022 by 0.47, corresponding to the mean positive prediction bias identified in model evaluation. Even under this substantial and directionally consistent perturbation, the key conclusions remained effectively unchanged.

Please see lines 253-258 for revisions in the main Text .

Based on the data-driven principles, we selected the Random Forest (R^2 =0.71) with the smallest prediction error for f_{NANI} and the Extra Tree (R^2 =0.53) for f_{NAPI}, respectively (Supplementary Fig. S20 & Supplementary Tables S3 & S4). We conducted additional uncertainty and sensitivity analyses to assess the influence of f_{NAPI} uncertainty on the main conclusions (Supplementary Text 10, Supplementary Figs. S21 & S22).

Please see lines 730-766 for revisions in the Supplementary Text 10: Uncertainty and sensitivity.

Supplementary Text 10: Uncertainty and sensitivity

4. Sensitivity of model inference to uncertainty in f_{NAPI}

In this study, f_{NAPI} was estimated using multiple state-of-the-art machine learning algorithms, among which Extra Trees yielded the best predictive performance. Nevertheless, model evaluation indicated a moderate predictive power, with a mean positive prediction bias of 0.47 on the test dataset, where 54.17% of samples were overestimated and 45.83% underestimated.

Rather than assuming f_{NAPI} to be precisely constrained, we explicitly accounted for this

uncertainty within a Bayesian hierarchical modeling framework. Compared to traditional deterministic or single-level statistical models, Bayesian hierarchical models are less sensitive to upstream uncertainty in forcing variables, as uncertainty can be partially absorbed at higher hierarchical levels and propagated probabilistically through the model structure. In addition, our inference relies primarily on binary response metrics, further reducing sensitivity to point-wise prediction errors in continuous input variables such as f_{NAPI}.

To directly assess the influence of potential systematic bias in f_{NAPI} on the final inference, we conducted a conservative sensitivity analysis by uniformly reducing f_{NAPI} values in 2017 and 2022 by 0.47, corresponding to the mean positive prediction bias identified during model evaluation. Even under this substantial modification, the key inferred patterns—including preferential nutrient retention, net retention, internal dominant model, remained effectively unchanged (Supplementary Table S10).

Table S10. Sensitivity of key model outcomes to systematic bias in f_{NAPI}

Key results	Main Text	Bias-corrected f_{NAPI} (−0.47)
2017		
Lakes showing preferential P retention (%)	14.88	17.36
Lakes showing preferential N retention (%)	85.12	82.64
N−P− (%)	6.61	6.61
N−P+ (%)	2.48	2.48
N+P− (%)	17.36	18.18
N+P+ (%)	73.55	72.73
2022		
Lakes showing preferential P retention (%)	7.44	5.79
Lakes showing preferential N retention (%)	92.56	94.21
N−P− (%)	14.88	14.88
N−P+ (%)	1.65	1.65
N+P− (%)	42.15	42.98
N+P+ (%)	41.32	40.50

We additionally reproduced all figures directly supporting the main conclusions using the bias-corrected f_{NAPI} values; the resulting patterns are visually consistent with those presented in the main

text, with only marginal differences in detail (Supplementary Fig. S21 & S22).

These results provide a concrete example supporting the methodological advantages discussed in Section 1 and the substantial reduction in uncertainty afforded by percentage-based categorical inference described in Section 3.

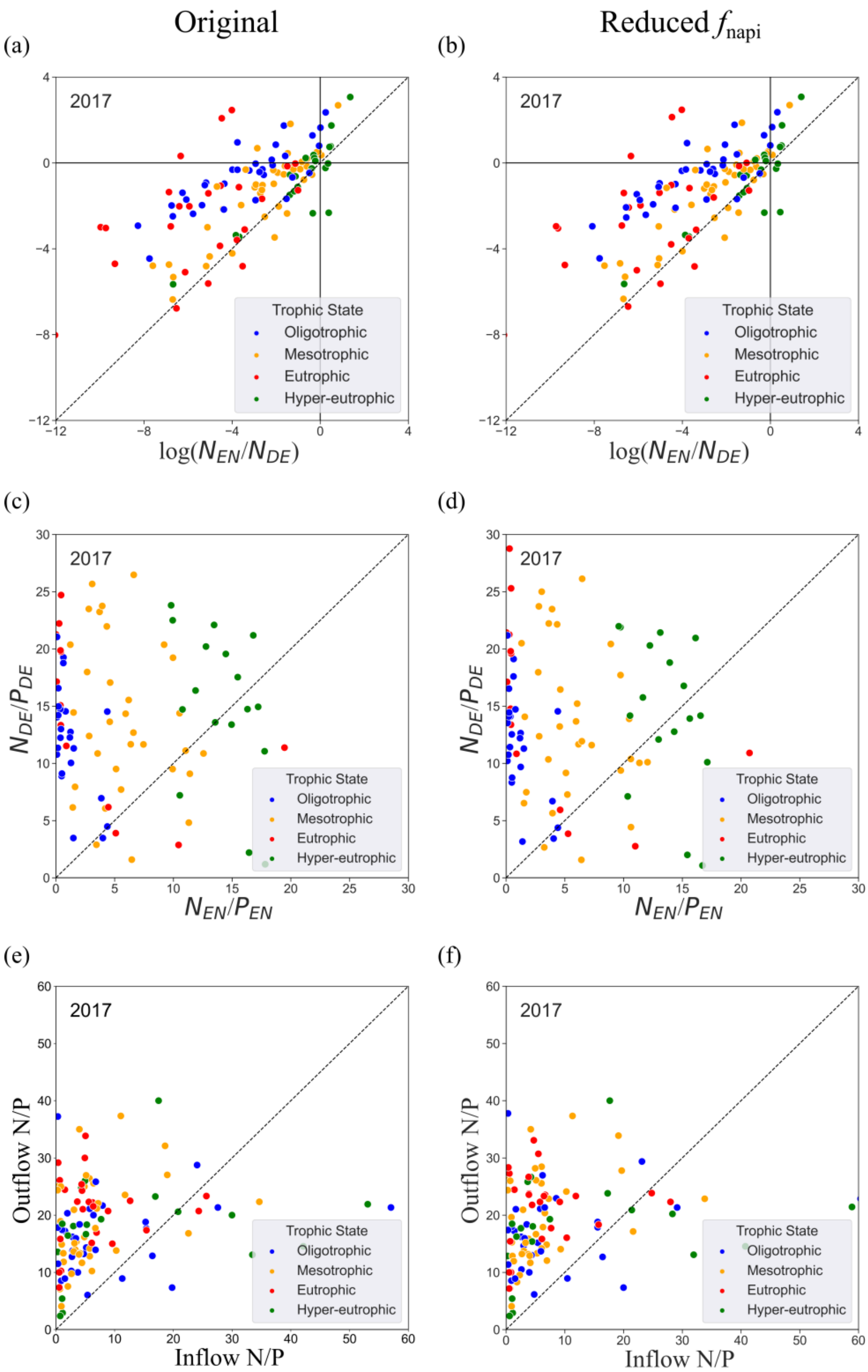

Figure S21 Sensitivity of model outputs to systematic bias correction in f_{NAPI} for 2017. Comparison of model outputs for 2017 using the original f_{NAPI} estimates (left panels, **(a), (c), (e)**) and bias-corrected f_{NAPI} values reduced uniformly by 0.47 (right panels, **(b), (d), (f)**),

corresponding to the mean positive prediction bias identified in model evaluation.

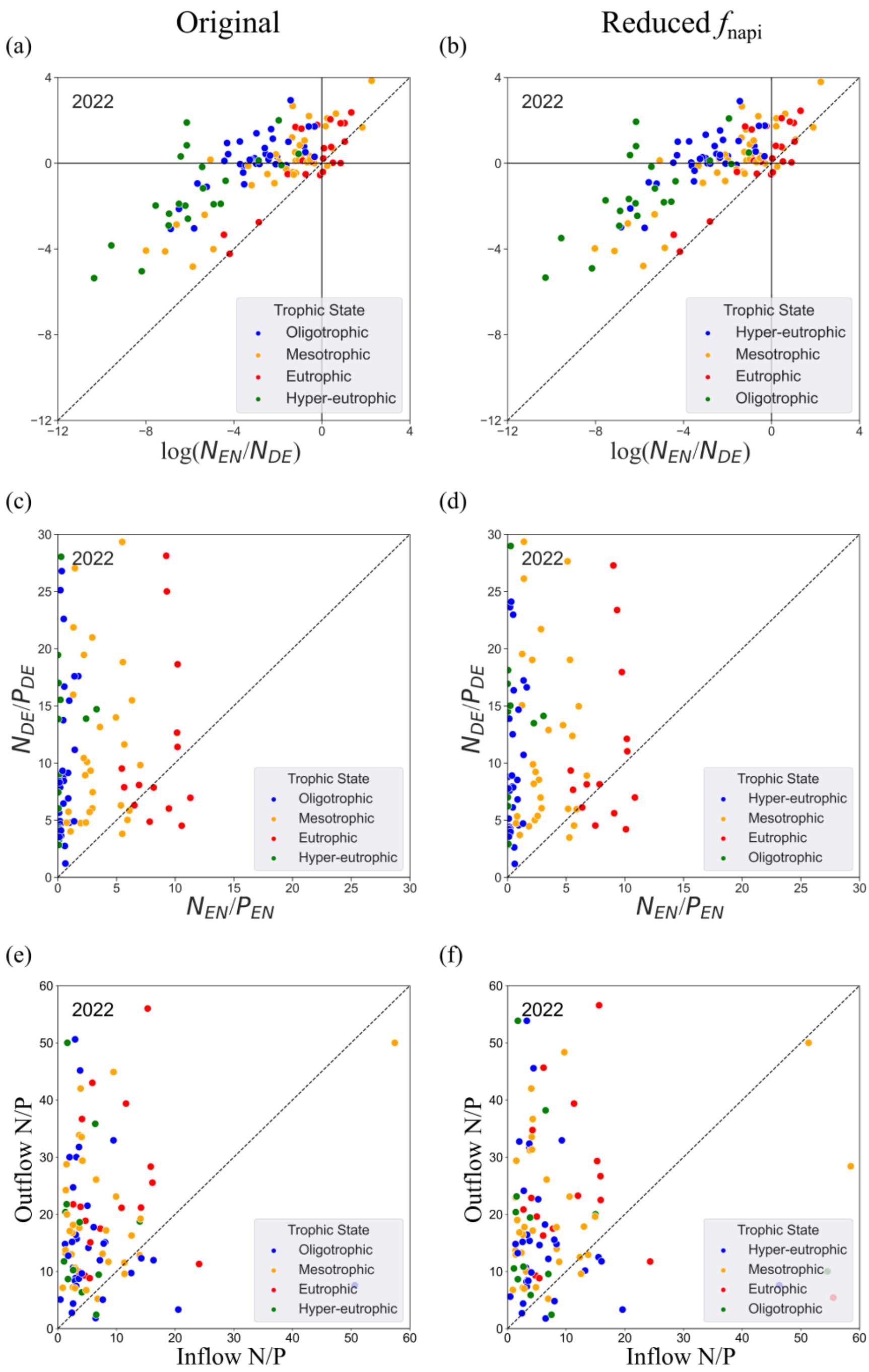

Figure S22 Sensitivity of model outputs to systematic bias correction in f_{NAPI} for 2022. Comparison of model outputs for 2022 using the original f_{NAPI} estimates (left panels, **(a), (c), (e)**) and bias-corrected f_{NAPI} values reduced uniformly by 0.47 (right panels, **(b), (d), (f)**), corresponding to the mean positive prediction bias identified in model evaluation.

12.Overall: The study tackles an important problem, and the dataset is valuable. The attempt to use a Bayesian nutrient-budget model alongside ML is interesting, and the focus on internal cycling is a genuinely useful angle. But the manuscript also stretches its conclusions well beyond what the data can support. Three snapshot years cannot justify claims about flickering, regime shifts, suppression mechanisms, or systems “adjusting nutrient preferences”. Several key definitions are unclear, figure references don’t line up, and important variables like INCI are not clear to me. The ML models feeding the Bayesian core have modest skill, yet the manuscript repeatedly claims “extremely low uncertainty”, which is not consistent with the model performance. In a few places, the narrative simply over-interprets patterns as causation. The idea has potential, but the conclusions need to be far more cautious and grounded in what the data can actually demonstrate.

【Response】We thank the reviewer for this thoughtful overall assessment and for recognizing the value of the dataset and the integrative Bayesian–ML framework. We fully acknowledge the concerns. In response, we have conducted additional analyses and strengthened the supporting evidence, and have systematically revised the Abstract, Results, and Discussion to soften the strength of conclusions, clarify key definitions, correct figure references, and ensure that all interpretations are firmly grounded in what the data can directly support. Claims related to flickering, regime shifts, internal cycling, and uncertainty have been consistently reframed in a more cautious and evidence-aligned manner. We believe these revisions address the reviewer’s overarching concerns and significantly improve the balance between novelty and interpretative rigor.

Eutrophication Dynamics: The Shift of Preferential Nutrient Retention and the Era of Nutrient Metabolic Disorder

Ran Zhou[1], Josep Peñuelas[2], Jordi Sardans[2], Yindong Tong[3], Jinlong Zhang[1], John A. Dearing[4], Meng Liu[1], Zisong Chen[1], Xinyi Wu[1], Shu Tao[1], Xilong Wang[1]*

Abstract

The relative retention of nitrogen (N) and phosphorus (P) in lakes and their roles in nutrient cycling remain debated. Although the United States has implemented long-term P control measures in lakes, inexplicable eutrophication exacerbation still reoccurred over the past two decades. Here we geographically track and analyze the nutrient retention changes of 121 lakes in the USA during 2012-2022 using a combination of machine learning and Bayesian hierarchical methods. We find that the preferential retention of P in 70.3% lakes in 2012 shifted to that of N in 85.1% lakes in 2017, and 92.6% lakes in 2022. Four key lake indicators exhibit flickering-like dynamics, which may help identify the nutrient metabolic disorder-like state in the aquatic ecosystem. Overall, our results show that eutrophication in U.S. lakes during 2012-2022 is advancing on the basis of preferential and net nutrient retention shift from P to N, flickering-like dynamics, and associated occurrence of the nutrient metabolic disorder-like state that are all predominantly driven by internal cycling instead of external loading. These

[1]College of Urban and Environmental Sciences, Peking University, Beijing, China.
[2]CSIC, Global Ecology Unit CREAF-CSIC-UAB, Bellaterra, Spain.
[3]School of Environmental Science and Engineering, Tianjin University, Tianjin, China.
[4]Palaeoecological Laboratory, Geography and Environment, University of Southampton, Southampton SO17 1BJ, UK.

patterns are consistent with the possibility of a regime shift rather than simple eutrophication recurrence, and provide insights relevant to lake management and water quality improvement strategies.

Reviewer #2 (Remarks on code availability):

13. I was not able to access the code because the repository says ‘files are restricted to users with access’ and requires a username and email. That would break reviewer anonymity, so I could not check the code.

【Response】We thank the reviewer for this comment. In accordance with the requirements of the Nature Communications editorial office, the source data have been deposited on Figshare. We have checked the link and confirmed that it is accessible.

Please see lines 456-462 for revisions in the main Text .

Code availability

The key codes to reproduce the study are available at Figshare (https://figshare.com/s/7c5e3bb1bd6e408adc15).

Data availability

All data that support the findings in the current study are available within the Article and its Supplementary Information. Source data including USL dataset, the f_{NANI} & f_{NAPI} data, and INCI data are available at Figshare (https://figshare.com/s/481c9bef67a527de5ae8).

Responses (manuscript ID: NCOMMS-25-70712A) to reviewers and editor/author checklist:

We sincerely thank all reviewers for their constructive comments and thoughtful revision suggestions on our manuscript. Their comments have been instrumental in refining our work and enhancing its overall quality. We have diligently revised the manuscript and supplied comprehensive point-to-point responses to adequately address all concerns raised.

The comments are in green color, and the revised text that appears in the main text and Supplementary Information is in blue color.

Editor/checklist:

Please note that we do not allow punctuation in the title, and it must be 15 words or fewer. Furthermore, please revise the title (1) to make it more accessible to a general audience and (2) to take Reviewer #2's concerns into account, ensuring that the title does not make an overly strong or causal claim. For example, one possible title is: Preferential nitrogen retention characterizes current eutrophication in United States lakes. You are also welcome to suggest an alternative title.

[Response] We sincerely thank you for the constructive feedback and the suggested title. We agree with the reviewer that the suggested title is more accessible and appropriately avoids overly strong causal claims. We have adopted the title you recommended as shown below: "Preferential nitrogen retention characterizes current eutrophication in United States lakes".

Preferential nitrogen retention characterizes current eutrophication in United States lakes

The final paragraph of the Introduction contains detailed methods. Please move this text to the start of the Results or, where appropriate, to the Methods, to keep the Introduction focused on background and study rationale.

[Response] We have removed the detailed methodological descriptions from the final paragraph of the Introduction as suggested. Only a brief overview of the approach and its rationale was retained to keep the Introduction section focus on background and study rationale. The detailed methods have been fully elaborated in the Methods section. Please see the revision as shown below (Lines 89 - 92 in the main text).

This framework leverages the high predictive accuracy of machine learning for estimating net anthropogenic nutrient inputs (f_{NANI} and f_{NAPI})[36,37], and the strong ability of Bayesian hierarchical models to handle uncertainties and internal nutrient cycling processes[38,39].

Reviewer #1:

Thank you for the revisions. My comments below are minor editorial corrections:

[Response] Thank you so much for your positive comments and revision suggestions. We have carefully revised the manuscript closely following your constructive comments and revision suggestions, which highly improved the quality and clarity of our manuscript.

Detailed edits:

1.line 68 change to have been polluted, not pollution

[Response] Thanks for your edit. The sentence has been revised as shown below (Lines 65 - 66 in the main text).

The lakes in the USA have been polluted and since many have been remediated, they are at the forefront of the development history of lake pollution remediation in the world.

2. lines 81-82 change to screened, compiled, analyzed

[Response] Thanks for your revision suggestions. We have carefully read and adhered to the formatting guidelines of NC journal. It is stated in the checklist that "Please use the present tense when describing the current work in the Introduction." The tense usage in the Introduction complies with this guideline (Lines 77 - 79 in the main text).

3.line 87 remove whether

[Response] Thanks for your edit. The sentence has been revised as shown below (Lines 83 - 84 in the main text).

have substantial changes in net N and P retention occurred?

4.line 245 remove This reminds that

[Response] Thanks for your edit. The sentence has been revised as shown below (Lines 203 - 205 in the main text).

One should not regard it as an ordinary wave of eutrophication, thus seriously underestimating its potential ecological risks.

5.line 350 remove Moreover

[Response] Thanks for your edit. The sentence has been revised as shown below (Lines 306 - 307 in the main text).

Net anthropogenic nitrogen input (NANI [kg N km^{-2} yr^{-1}]) and phosphorus input (NAPI [kg P km^{-2} yr^{-1}]) were used in in-lake nutrient budget model.

Reviewer #2:

Thank you for the careful revision and detailed response. The manuscript has improved substantially compared with the previous version, and the authors have made a genuine effort to address the reviewers' concerns. The topic is interesting and potentially important for the field, particularly given the broad implications for understanding nutrient dynamics and eutrophication management in lakes. In my view, the revision has clearly strengthened the manuscript in several useful ways. The presentation is improved, a number of earlier ambiguities have been clarified, and the additional analyses and source data deposition have made the study more transparent. I also appreciate that the authors have moderated some of the earlier language.

[Response] We are deeply grateful to the reviewer for the positive evaluation and for recognizing the improvements made in this revision.

1. That said, I think the manuscript would still benefit from one further tightening of the interpretation in a few key places. In particular, the language around flickering-like dynamics, critical transitions, and regime shift should be kept consistently cautious throughout. With only three time points, the data may support the idea of patterns consistent with instability, but not a firm diagnosis of flickering or a regime shift in the stronger sense. I would therefore recommend revising these statements so that they are framed explicitly as a plausible interpretation or hypothesis supported by the observed patterns, rather than as a conclusion that has already been demonstrated. That would make the paper more rigorous and, frankly, harder to shoot down. A similar point

applies to the discussion of the relationship between preferential N retention and suppression of net P retention. The revised analyses do improve this part, but the manuscript should still avoid language that sounds more causal than the evidence allows. I suggest that the authors consistently use wording such as associated with, consistent with, or may contribute to, unless they can provide stronger evidence for causality. This is not just semantics; it matters because the current formulation risks overstating what the analysis can actually establish. A slightly more careful framing would preserve the novelty of the result while keeping the interpretation on solid ground.

[Response] We thank the reviewer for this insightful and constructive suggestion. We fully agree that the interpretations regarding flickering-like dynamics, critical transitions, regime shifts, and the relationship between preferential N retention and net P retention must remain appropriately cautious.

In response, we have systematically revised the relevant sections throughout the manuscript by replacing definitive language with more measured phrasing. We now consistently use expressions such as “is consistent with”，“patterns consistent with”，“may”，“suggests that… may” to clearly frame these as plausible interpretations supported by the observed data rather than firm conclusions.

As a response to the editor’s comment, the title of this manuscript has been revised as shown below. It is exactly the one that the editor recommended in the checklist.

Preferential nitrogen retention characterizes current eutrophication in United States lakes

The sentence has been revised as shown below (Lines 200 - 201 in the main text).

The seemingly slowly-increasing eutrophication trend is consistent with the lakes facing conditions that may precede a regime shift.

The sentence has been revised as shown below (Lines 168 - 170 in the main text).

The occurrence of flickering-like dynamics suggests patterns consistent with approaching a critical point in nutrient cycling.

The sentence has been revised as shown below (Lines 206 - 207 in the main text).

the NMD-like state may pose a substantial risk of inflicting severe ecological and socioeconomic consequence if not mitigated in a timely manner.

The sentence has been revised as shown below (Lines 276 - 277 in the main text).

This hitherto undetected phenomenon is consistent with the possibility that the new eutrophication wave of lakes is undergoing a regime shift.

2. On reproducibility, the provision of source datasets and Stan model files is helpful and addresses the earlier access issue. However, if the authors wish to claim that the study can be reproduced from the deposited materials, I would encourage them to provide the fuller computational workflow as well, including the scripts used for preprocessing, model execution, and generation of derived outputs and figures, or otherwise moderate the wording of the code availability statement accordingly.

[Response] We thank the reviewer for this valuable feedback on reproducibility. We have now addressed this concern by providing the full computational workflow, including all scripts. The code availability statement has also been updated accordingly to accurately reflect the complete reproducibility of the study. Source data files for each graph and chart are also publicly available.

The sentence has been revised as shown below (Lines 410 - 417 in the main text).

Data availability

The input data including the USL dataset, the f_{NANI} & f_{NAPI} data, and INCI data, have been deposited in the Figshare database (https://doi.org/10.6084/m9.figshare.31259455). Source Data files used to derive the reported outputs and figures are available at Figshare (https://doi.org/10.6084/m9.figshare.32311506).

Code availability

The key codes to reproduce the study are available at Figshare (https://doi.org/10.6084/m9.figshare.32297847).

3. Overall, I find the manuscript improved and potentially publishable, but I believe one further round of tightening would strengthen it considerably. The main concern now is a more disciplined alignment between the strength of the claims and the actual strength of the evidence.

[Response] We sincerely thank the reviewer for the positive assessment and for the constructive final suggestion. We fully agree that a tighter alignment between the strength of the claims and the supporting evidence is essential. We believe the manuscript is now substantially strengthened and it meets the reviewer's expectations for publication.

4. I reviewed the deposited materials on Figshare, but in my view they do not yet provide a fully reproducible workflow for the study. The repository appears to include the Stan model files and processed datasets, which is helpful, but it does not seem to contain the full computational pipeline needed to reproduce the results end to end. In particular, I could not identify a clear README, execution instructions, preprocessing scripts, machine-learning workflow for generating fNANI/fNAPI, the scripts used to run the Bayesian models, or the code used to derive the reported outputs and figures. As such, the deposit is useful for inspection of model structure and input data, but not sufficient for full reproducibility in practice. I was therefore not able to verify the results by running the code directly. In its current form, I would not regard the code as a readily usable community resource.

[Response] Thanks for your edit. As responded to your comment 2 above, we have now addressed this concern by providing the full computational workflow. The code availability statement has been updated accordingly to accurately reflect the complete reproducibility of the study. Source data files for each graph and chart are also publicly available.
